# New Brassinosteroid Analogs with 23,24-Dinorcholan Side Chain, and Benzoate Function at C-22: Synthesis, Assessment of Bioactivity on Plant Growth, and Molecular Docking Study

**DOI:** 10.3390/ijms25010419

**Published:** 2023-12-28

**Authors:** Vanessa Aitken, Katy Diaz, Mauricio Soto, Andrés F. Olea, Mauricio A. Cuellar, Maria Nuñez, Luis Espinoza-Catalán

**Affiliations:** 1 Departamento de Química, Universidad Técnica Federico Santa María, Avenida España 1680, Valparaíso 2340000, Chile; vanessa.aitken.13@sansano.usm.cl (V.A.); katy.diaz@usm.cl (K.D.); mauricio.sotoc@usm.cl (M.S.); maria.nunezg@sansano.usm.cl (M.N.); 2 Grupo QBAB, Instituto de Ciencias Aplicadas, Facultad de Ingeniería, Universidad Autónoma de Chile, El Llano Subercaseaux 2801, Santiago 8900000, Chile; 3 Facultad de Farmacia, Escuela de Química y Farmacia, Universidad de Valparaíso, Av. Gran Bretaña 1093, Valparaíso 2340000, Chile; mauricio.cuellar@uv.cl; 4 Centro de Investigación Farmacopea Chilena (CIFAR), Universidad de Valparaíso, Valparaíso 2340000, Chile

**Keywords:** brassinosteroid analogs, synthesis, plant growth, molecular docking

## Abstract

The synthesis and biological evaluation of brassinosteroids (BRs) analogs with chemical modification in the side alkyl chain is a matter of current interest. Recently, a series of BR analogs with phenyl or benzoate groups in the alkyl chain have been reported. The effect of substitution in the aromatic ring on the biological activities of these new analogs has been evaluated, and the results suggest that the bioactivity is enhanced by substitution with an F atom. In this context, we have synthesized, characterized, and evaluated a series of new analogs of 23,24-bisnorcholenic type in which the benzoate group at the C-22 position is substituted with an F atom at “*ortho* or *para*” positions. Plant growth-promoting activities were evaluated by using the rice lamina inclination test and bean second internode biotest. The results obtained with both bioassays indicate that the compound with an F atom in the para position on the aromatic ring is the most active BR analog and in some cases is even more active than brassinolide. The docking study confirmed that compounds with an F atom adopt an orientation similar to that predicted for brassinolide, and the F atom in the “*para*” position generates an extra hydrogen bond in the predicted binding position.

## 1. Introduction

Brassinosteroids (BRs) are an important group of polyhydroxylated naturally occurring steroidal phytohormones found in the plant kingdom in extremely low amounts [1]. This phytohormone group regulates plant growth and development by producing an array of physiological changes and eliciting very important functions, such as plant growth regulation and cell division and differentiation in young tissues of growing plants [2,3,4,5,6]. They also play an important role in molecular and physiological responses under abiotic stress such as drought, salinity, high temperature, low temperature, and heavy metal stresses [7,8,9].

Due to the low concentrations in which these compounds are found, much effort has been dedicated to synthesize these compounds or their structural analogs using natural and abundant sterols [10]. One of the main difficulties found in the synthesis of BRs is the replication of the side alkyl chain due to the presence of at least three chiral centers. Thus, in the quest for new active analogs, various BRs analogs have been obtained by incorporating important structural modifications in their side chain [11,12,13,14,15,16,17,18,19,20,21,22,23,24,25,26,27,28,29,30,31,32,33,34,35,36].

**Figure 1:**
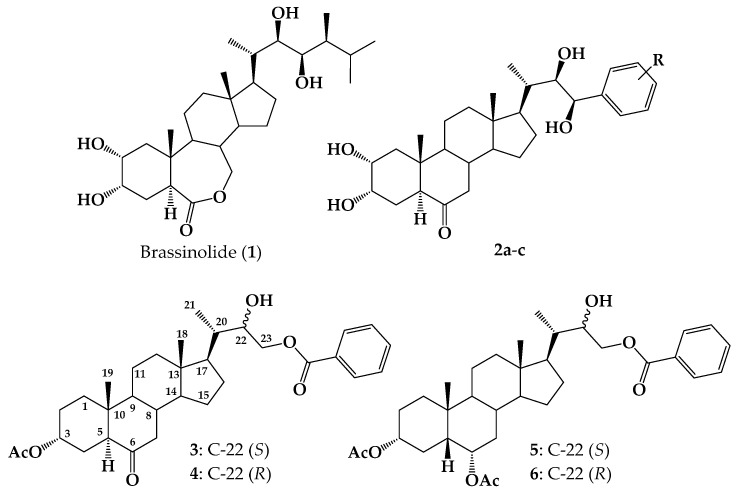
Structure of brassinolide (**1**), arylbrassinosteroids **2a**–**c,** and brassinosteroid analogs 24-Norcholane with benzoate function at C-23 (compounds **3**–**6**).

Recently, the synthesis of BRs analogs carrying phenyl groups, with no or small nonpolar substituents, in the side alkyl chain has been reported [37,38]. A comparison of experimental activity on plants (ethylene production or inhibitory effect on *Arabidopsis* root growth) with a molecular structure indicates that the most active analogs are those with no substitution or substituted with chlorine or fluorine atoms in “*ortho*, *meta* and *para*” positions of the aromatic ring (compounds **2a** (*o*-F), **2b** (*p*-F), and **2c** (*m*-Cl), Figure 1). From this new series of BRs analogs, compound **2a**, with a F atom in the ortho position, is the most active and promising compound. These results are confirmed by molecular docking into BRI 1 studies [37,38]. In that direction, we have reported the synthesis of new BRs analogs of 24-Norcholanes type, with benzoate functions at the C-23 position of the side chain (compounds **3** and **4**, Figure 1).

The bioactivity of BRs analogs **3** and **4** was assessed by using the rice lamina inclination test (RLIT) and root elongation in *Arabidopsis thaliana*. Both compounds show similar biological activity at 1 × 10^−8^, 1 × 10^−7^, and 1 × 10^−6^ M concentrations, with **4** being slightly more active in the RLIT test. Interestingly, compound **3** was found to be more active than brassinolide (**1**) (Figure 1) at all tested concentrations [39]. On the other hand, compounds **5** and **6** were also evaluated in RLIT, and the **5**/**6** diastereoisomer mixture (ratio 1:0/0.44) is significantly more active than brassinolide at 1 × 10^−8^ M concentration [40]. These results suggest that the incorporation of a benzoate group at the C-23 position of the side chain induces an increase in the biological activity of these compounds.

It is well established that BRs perception occurs in a two-step mechanism, namely binding to the BRI1 (Brassinosteroid Insensitive 1) receptor and induced heterodimerization with a second receptor kinase, BAK1 (BRI1-Associated Receptor Kinase 1) [41,42]. The formation of this ternary complex results in full BRI1 activation, initiating a signaling cascade that regulates a series of physiological processes [43,44]. Thus, minor modifications to the BR structure may produce important changes in binding to this receptor [37,45]. These effects can be evaluated via molecular docking into the BRI1–BAK1 crystallized complex (PDB: 4m7e BRI-1), which provides information about poses and binding energies adopted by BRs analogs in the interaction with this receptor.

Thus, with the aim of evaluating the effect of the nature and position of a benzoate ring substituent on biological activity, herein, we report the synthesis of three new analogs of 23,24-bisnorcholenic type in which the benzoate group at C-22 position is substituted with a fluorine atom at “*ortho* or *para*” positions. Growth-promoting activities were evaluated by using RLIT and the bean second internode (BSI) biotest. Finally, experimental results are compared with those predicted by a study of molecular docking into the BRI1–BAK1 crystallized complex. The discussion of these results could be of great help in the understanding of bioactivity structural requirements associated with the BRs side chain structure.

## 2. Results and Discussion

**Figure 2:**
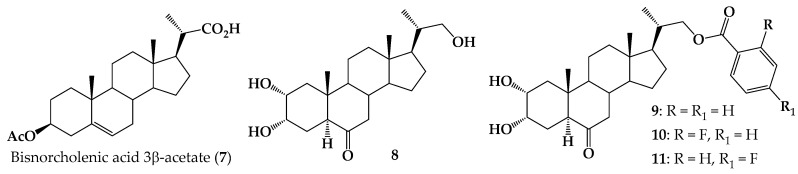
Structure of bisnorcholenic acid 3β-acetate (**7**), BR analog **8**, and new analogs 23,24-dinorcholenic **9**–**11** (pregnanes) with benzoate function at C-22.

BR analogs **8**–**11** were synthesized from a commercially starting material, namely (20S)-3β-acetoxy-5-pregnen-20-carboxylic acid (**7**) (Steraloid Inc. New York, NY, USA, bisnor-cholenic acid 3β-acetate) (Figure 2).

### 2.1. Chemical Synthesis

The synthetic route used to obtain BRs analogs **8**–**11** is described in Scheme 1 and Scheme 2. In Scheme 1 is shown a series of reactions, which, starting from (**7**) and following reported procedures [46,47], gives compound **17**. Experimental details and reaction yields are given in the Experimental Section. Melting points, IR, and ^1^H NMR spectroscopic data obtained for these compounds were consistent with those reported [47]. Additionally, to complete the available spectroscopic information, ^13^C, ^13^C DEPT-135, 2D HSQC, and 2D HMBC NMR experiments were carried out (Supplementary Material Figures S1–S24).

**Scheme 2:**
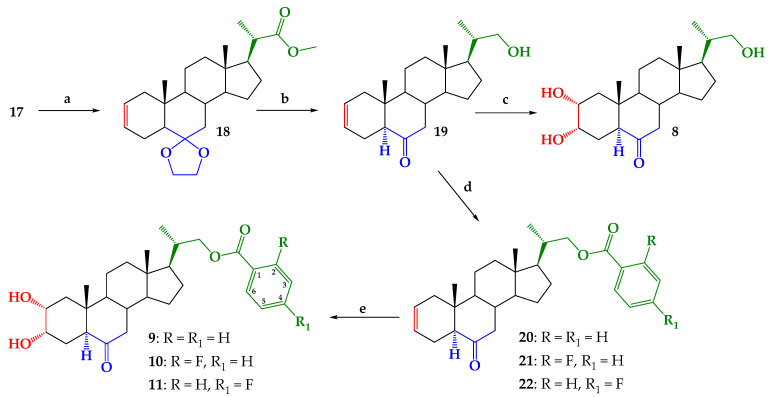
Synthesis of compounds **18**–**22**, analog **8**, and new BRs analogs **9**–**11**. Conditions: (**a**) HOCH_2_CH_2_OH/TsOH/C_6_H_6_, reflux, 2.5 h, 99.0% yield; (**b**) (1) LiAlH_4_ (2M)/THF, r.t. 90 min, (2) HCl (5% *w*/*w*), r.t. 1 h, 91.5% yield; (**c**) DHQD-CLB, CH_3_SO_2_NH_2_, K_2_CO_3_, K_3_[Fe(CN)_6_], OsO_4_, t-BuOH/H_2_O (1:1 *v*/*v*), r.t. 36 h, 90% yield; (**d**) p-PhCOCl/CH_2_Cl_2_/py, DMAP, r.t. 2h, 85.4%, 65.0%, and 72.5% yields for **20**, **21,** and **22**, respectively; (**e**) DHQD-CLB, CH_3_SO_2_NH_2_, K_2_CO_3_, K_3_[Fe(CN)_6_], OsO_4_, t-BuOH/H_2_O (1:1 *v*/*v*), r.t. 36 h, 90.0%, 82.5%, and 84.9% yield for **9**, **10,** and **11,** respectively.

In Scheme 2 is shown a series of reactions, which, starting from **17,** allow the obtention of new BRs analogs **8**–**11** and compounds **18**–**22**.

**Figure 3:**
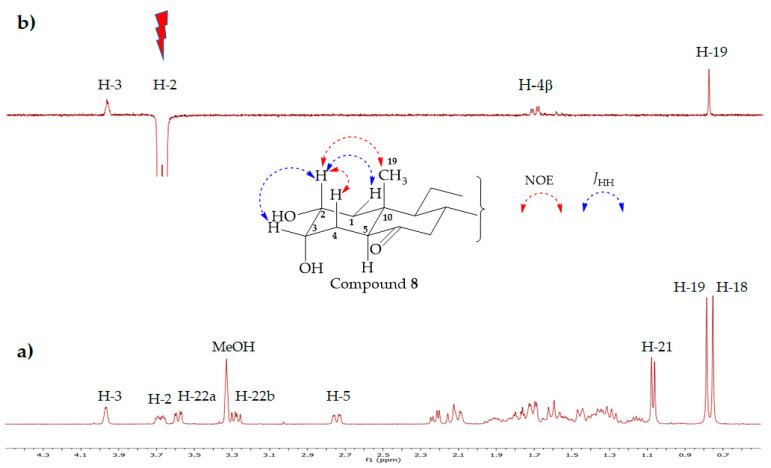
(**a**) ^1^H NMR (MeOD) spectrum of compound **8**. (**b**) Selective 1D NOE spectrum, showing the selective excitation of H-2 and the NOE effects observed with H-4β and CH_3_-19. The other signal observed corresponds to the scalar coupling of H-2 with H-3.

Initially, the keto group of **17** was protected with ethylene glycol/TsOH/C_6_H_6_, following a procedure that has been described for steroids of similar structure [48]. The expected dioxolane derivative **18** was obtained with a 99.0% yield (Scheme 2), and the presence of a dioxolane ring was established via ^1^H and ^13^C NMR spectroscopy. Signals observed at δ_H_ = 3.91–3.80 ppm (3H, m) and δ_H_ = 3.73–3.68 ppm (1H, m) were assigned to four hydrogens of the oxolane ring, whereas signals observed at δ_C_ = 109.70, 65.38, and 63.90 ppm were assigned to C-6 and O-CH_2_CH_2_-O- (dioxolane moiety) (Supplementary Material Figures S25–S29). A subsequent reduction of **18** leads to hydroxy-ketone **19** with a 91.5% yield. Further, 1D and 2D NMR spectra are shown in Figures S30–S34 (Supplementary Material) [48]. Sharpless dihydroxylation of the double bond on C-2 in compound **19** produces triol **8** with a 70.9% yield. It is widely known that this reaction is stereo-controlled by A-ring conformation and the presence of a methyl group on C-10. The attack of a reagent (OsO_4_) is always from the bottom side of the molecule [37]. Analog **8** has been previously prepared, but its structure was only characterized by IR spectroscopic data [49]. Herein, the complete structural determination of **8** was established by using ^1^H and ^13^C NMR spectroscopic techniques. So, the signals observed at δ_H_ = 3.95 ppm (1H, d, *J* = 2.8 Hz) and 3.66 ppm (1H, ddd, *J* = 11.7, 4.7, and 3.1 Hz) were assigned to carbynolic hydrogens H-3 and H-2, respectively. On the other hand, the signals observed at d_C_ = 69.58 and 69.23 ppm were assigned to the carbynolic carbons C-3 and C-2, respectively. Additionally, these signals were correlated with 2D HSQC and 2D HMBC spectra (see Supplementary Material Figures S35–S40). The spatial orientation of hydroxyl groups 2α,3α-diol was established through selective 1D Noesy experiments, where the signal of H-2 (δ_H_ = 3.66 ppm) showed spatial correlations with H-4β and CH_3_-19, indicating that these H atoms have the same spatial β-orientation (Figure 3).

Benzoylation of a primary alcohol in C-22 of hydroxy-ketone **19** with the corresponding benzoyl chlorides [16,50] produces compounds **20**–**22** with 85.4%, 65.0%, and 72.5%, yields, respectively. Compounds **20**–**22** were fully characterized using 1D and 2D NMR spectroscopic techniques (see Figures S41–S55, Supplementary Material). Finally, new BRs analogs **9**–**11** were obtained by the sharpless dihydroxylation of olefins **20**–**22**, with 90.0%, 82.5%, and 84.9% yields, respectively. Compounds **9**–**11** were fully characterized using IR, 1D, 2D NMR, and HRMS spectroscopic techniques (see Figures S56–S70, Supplementary Material).

### 2.2. Biological Activity

BRs play important roles in plant growth, development, and responses to abiotic stresses. It is well established that these effects are due mainly to the regulation of cell expansion, cell division, and cell elongation. Thus, BRs activity can be detected and quantified through a series of different plant responses, and, consequently, various bioassays have been proposed. It has been shown that different BRs activities are obtained by using different biological activity tests; this means that the results obtained by using different methods are not equivalent and cannot be compared [40]. This is a very important factor to be considered for a comparison of BRs activities and the proposal of structure–activity relationships of synthetic BRs analogs [51,52]. Herein, we used the rice lamina inclination test (RLIT), one of the most widely used methods due to its high sensitivity and specificity, and the bean second internode bioassay.

#### 2.2.1. Bioactivity in the Rice Lamina Inclination Test (RLIT) of Brassinosteroid Analogs

The activity of BRs analogs **9**–**11** was assessed by using RLIT and using brassinolide as a positive control. Measurements were performed at two different concentrations, namely 1 × 10^−7^ M and 1 × 10^−6^ M (see Table 1). The results are given as the bending angle between laminae and sheaths.

The values of bending angles obtained for **1**, at both tested concentrations, are in line with those reported in a previous work [17] and indicate that bioactivity of **1** increases with increasing concentration. It can also be observed that analogs **10** and **11**, with a benzoate group at C-22 and a fluorine atom in the *ortho* and *para* positions, respectively, are more active than analog **8** (no benzoate group) and analog **9** (no F atom in the benzoate group) at both tested concentrations (Table 1). Interestingly, at 1 × 10^−7^ M, analog **10** is the most active compound, and its activity is even comparable with that shown by brassinolide. However, at 1 × 10^−6^, M the brassinolide activity is 3 to 9 times higher than that exhibited by BRs analogs **8**–**11**. Thus, a comparison of bending angles measured for **9** with those obtained for **10** and **11** shows that substitution by an F atom in the benzoate group increases plant growth elongation, and this effect depends on the position of the F atom.

#### 2.2.2. Bioactivity in Bean Second Internode Bioassay of Brassinosteroid Analogs

**Figure 4:**
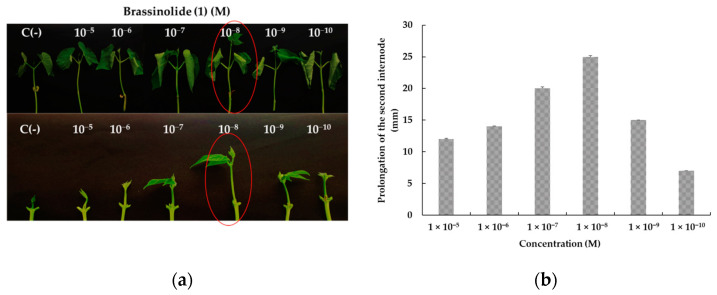
Dose–response curve of compound **1** used as positive control 4 days after application at different concentrations: (**a**) visual representation; (**b**) effect of compound **1** on the prolongation of the second internode of the bean. Error bars represent S.D.

This test has been used in both gibberellins and BRs. For gibberellins, only elongation can be detected, while for BRs curvature, swelling and division of the internode are easily measured [46,47,53,54,55,56,57,58]. The effect on plant tissues depends not only on the structural conformation of the phytohormones but also on the concentration applied, so a dose–response curve of **1** (1 × 10^−5^–1 × 10^−10^ M, Figure 4a,b) was obtained to determine the optimal concentration at which tested molecules should be applied.

The data show that brassinolide activity in the bean second internode bioassay is maximum at 1 × 10^−9^ M. Therefore, the effect of BRs analogs was measured at this concentration using **1** as a positive control.

**Figure 5:**
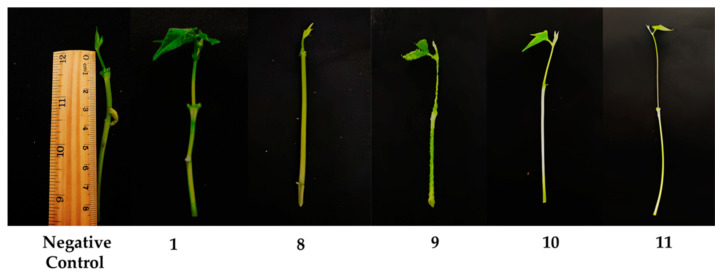
Effect of BRs analogs **8**–**11** and positive control (**1**) on the elongation bean second internode bioassay at a concentration of 1 × 10^−8^ M.

The values obtained from the elongation test of the second internode of the bean are shown in Figure 5 and summarized in Table 2.

Data in Table 2 show that the activity of benzoylated analogs **9** and **10** measured by using the bean second internode bioassay is like that found for brassinolide, whereas analog **11** (F atom in *para* position) exhibits a surprisingly high activity in elongation. Finally, analog **8** (no benzoate function in the side chain) is inactive at the same concentration.

Therefore, the results obtained with both bioassays indicate that compound **11** with fluorine atom para-substituted on the aromatic ring is the most active BRs analog and in some cases is even more active than brassinolide.

### 2.3. Molecular Docking Studies

To gain a deeper understanding of how plant activity of BRs analogs is influenced by their molecular structure, a molecular docking study was carried out. This process was performed by placing these compounds within the active site of the BRI1–BAK1 crystallized complex (PDB: 4m7e) using AutoDock Vina.

To assess the effectiveness of the analysis, a redocking of brassinolide into the crystallographic structure of BRI1-BAK1 was performed, and the best-resulting pose was compared with the original ligand’s pose. The outcomes reveal a remarkable resemblance between the obtained pose with the lowest energy and the crystallographic pose. Therefore, these parameters were deemed appropriate and were used for docking of synthetic ligands. The results indicate that the poses of each ligand within the binding site exhibit a strong alignment with brassinolide, and calculated binding energies for these complexes ranged from −11.9 to −13.2 kcal/mol, which suggests a favorable inclination toward the creation of BRI1-ligand-BAK1 complexes (see Table S1, Supplementary Material).

**Figure 6:**
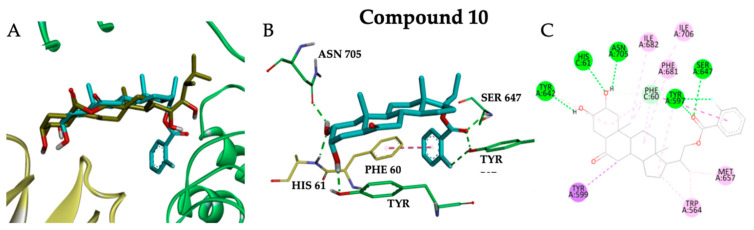
Binding modes of compound **10** into BRI1–BAK1 heterodimer. Binding modes of **10** are represented as turquoise and red sticks. (**A**) Poses of **10** and brassinolide (brown sticks) within the BRI1-BAK1 binding site; (**B**) pose of **10** and its interaction within the BRI1-BAK1 binding site; (**C**) a 2D view of binding interactions of **10** within the binding site. Hydrogen bonds are represented in green segmented lines. π-π stacking is represented in dark pink segmented lines. Hydrophobic interactions are represented in pink segmented lines.

As can be seen in Figure 6A, compound **10** adopts an orientation similar to that found for brassinolide. In the predicted binding position of **10** (Figure **6**), six hydrogen bonds were identified. Two of these bonds are formed by the carbonyl group of ArCO, interacting with Tyr 597 and Ser 647 at distances of 2.25 Å and 2.27 Å, respectively. The others are created by interactions between the hydroxyl group at position C-2 and His 61 (at 2.04 Å) and Asn 705 (at 2.09 Å). Additionally, the hydroxyl group at C-3 forms a hydrogen bond with Tyr 642 at 2.13 Å. Another hydrogen bond was observed between the fluorine atom and Tyr 597, with a 2.80 Å distance. Furthermore, the Phe 60 residue engages in π-π stacking with the aromatic ring of compound **10**. Finally, **10** participates in van der Waals interactions with Trp 564, Met 657, Phe 681, Ile 706, Ile 682, Phe 60, Tyr 597, Tyr 642, and Tyr 599 (Figure S76, Table S2, Supplementary Material).

On the other hand, compound **11** adopts a similar orientation to that of compound **10** and brassinolide (Figure S76, Supplementary Material). At the predicted binding position of **11**, six hydrogen bonds were identified, five of which are consistent with those identified in compound **10** (Tyr 597 at 2.25 Å, Ser 647 at 2.33 Å, His 61 at 1.96 Å, Asn 705 at 2.57 Å, and Tyr 642 at 2.21 Å). However, the fluorine atom of the benzoate group in the “*para*” position generates a hydrogen bond with Ser 48 at 2.88 Å. Furthermore, the Phe 60 residue participates in π-π stacking with the aromatic ring of compound **11**. On the other hand, **11** generates van der Waals interactions with Trp 564, Met 657, Phe 681, Ile 706, Ile 682, Phe 60, Tyr 597, Tyr 642, and Tyr 599 (Figure S76, Table S2, Supplementary Material).

It is important to highlight that compound **8**, which has the shortest side chain, exhibits low affinity with the BRI1-BAK1 active site, consistent with the results obtained in the biological activity study. Conversely, our results suggest that analogs with a benzoate function at C-22 show improved affinity with the BRI1-BAK1 active site, and the presence of a fluorine atom in the “ortho and para” position strengthens this interaction, as observed in Figure 4C and Figure S76 of Supplementary Material.

## 3. Materials and Methods

### 3.1. General Chemical

All reagents were purchased from commercial suppliers and used without further purification. Melting points were measured on an SMP3 apparatus (Stuart-Scientific, now Merck KGaA, Darmstadt, Germany) and are uncorrected. ^1^H-, ^13^C-, ^13^C DEPT-135, gs 2D HSQC, and gs 2D HMBC NMR spectra were recorded in CDCl_3_ solutions and were referenced to the residual peaks of CHCl_3_ at δ = 7.26 ppm and δ = 77.00 ppm for ^1^H and ^13^C, respectively, on an Avance Neo 400 Digital NMR spectrometer (Bruker, Rheinstetten, Germany) operating at 400.1 MHz for ^1^H and 100.6 MHz for ^13^C. Chemical shifts are reported in δ ppm, and coupling constants (*J*) are given in Hz; multiplicities are reported as follows: singlet (s), doublet (d), broad doublet (bd), doublet of doublets (dd), doublet of triplets (dt), triplet (t), broad triplet (bt), quartet (q), doublet of quartet (dq), doublet of double doublets (ddd), triplet of triplets (tt), and multiplet (m). IR spectra were recorded as KBr disks in an FT-IR 6700 spectrometer (Nicolet, Thermo Scientific, San Jose, CA, USA), and frequencies are reported in cm^−1^. High-resolution mass spectra (HRMS-ESI) were recorded in a Bruker Daltonik (Bruker, Bremen, Germany). The analysis for the reaction products was performed with the following relevant parameters: dry temperature, 180 °C; nebulizer 0.4 Bar; dry gas, 4 L/min; and spray voltage, 4.5 kV at positive mode. Accurate mass measurements were performed at a resolving power: 140,000 FWHM at range *m*/*z* 50–1300. For analytical TLC, silica gel 60 in a 0.25 mm layer was used, and TLC spots were detected by heating after spraying with 25% H_2_SO_4_ in H_2_O. Chromatographic separations were carried out by using a conventional column on silica gel 60 (230–400 mesh) using EtOAc-hexane gradients of increasing polarity. All organic extracts were dried over anhydrous magnesium sulfate and evaporated under reduced pressure below 40 °C.

### 3.2. Synthesis

#### 3.2.1. Methyl (20S)-3β-Acetoxypregn-5-ene-20-carboxylate (**12**)

To a solution of bisnorcholenic acid 3β-acetate (**7**) (3.02 g, 7.77 mmol) in 30 mL of ether, 180 mL of ethereal CH_2_N_2_ was added. The reaction mixture was kept under constant stirring and room temperature for 6 h. The end of the reaction was verified via TLC, and the mixture was then concentrated to dryness under reduced pressure. The solid obtained was recrystallized from ether/CH_2_Cl_2_ (1:1). Compound **12** (3.12 g, 99.8% yield) was obtained as a colorless solid (m.p. = 147.9–152.8 °C (144–146 °C [47]). IR_νmax_ (cm^−1^): 3060 (C=C-H); 2938 (CH_3_-); 2898 (CH_2_-); 2848 (CH_2_-); 1734 (C=O); 1715 (C=O); 1466 (CH_2_-); 1386 (CH_3_-); 1262 and 1065 (C-O). ^1^H NMR (400.1 MHz, CDCl_3_): δ (ppm) = 5.36 (1H, bd, *J* = 5.1 Hz, H-6); 4.63–4.55 (1H, m, H-3); 3.64 (3H, s, COCH_3_); 2.42 (1H, dq, *J* = 10.8 and 6.9 Hz, H-20); 2.02 (3H, s, CH_3_CO); 1.19 (3H, d, *J* = 6.7 Hz, H-21); 1.01 (3H, s, H-19); 0.68 (3H, s, H-18). ^13^C NMR (100.6 MHz, CDCl_3_): δ (ppm) = 177.30 (C-22); 170.51 (COCH_3_); 139.62 (C-5); 122.47 (C-6); 73.90 (C-3); 56.22 (C-14); 51.31 (OCH_3_); 52.84 (C-17); 49.93 (C-9); 42.46 (C-13); 42.38 (C-20); 39.47 (C-12); 38.08 (C-4); 36.96 (C-1); 36.56 (C-10); 31.84 (C-7); 31.79 (C-8); 27.73 (C-2); 27.12 (C-16); 24.31 (C-15); 21.42 (CH_3_CO); 20.93 (C-11); 19.29 (C-19); 17.11 (C-21); 12.00 (C-18). IR and ^1^H NMR spectroscopic data were consistent with those reported [36,46,47].

#### 3.2.2. Methyl (20S)-3β-Hydroxy-pregn-5-ene-20-carboxylate (**13**)

Compound **12** (3.07 g, 7.64 mmol) and sodium hydrogen carbonate (2.35 g, 27.98 mmol) were dissolved in a mixture of methanol (300 mL) and water (25 mL) and then heated at reflux and constant stirring for 2 h. The end of the reaction was verified via TLC and then taken to dryness under reduced pressure. The reaction crude was diluted with a solution of HCl (pH = 4; 70.0 mL) until a white solid was formed. The aqueous phase was extracted with AcOEt (2 × 30 mL), washed with water (2 × 50 mL), and dried with Mg_2_SO_4_. Then, the solvent was evaporated in vacuo to obtain pure compound **13** (2.73 g, 7.57 mmol, 99.1% yield), which was obtained as a colorless solid (m.p. = 142.3–144.1 °C (141–142 °C [36,47]). IR_νmax_ (cm^−1^): 3497–3417 (O-H); 2938 (CH_3_-); 2896 and 2849 (CH_2_-); 1735 and 1716 (COO); 1459 (CH_2_-); 1382 (CH_3_-); 1274 and 1055 (C-O). ^1^H NMR (400.1 MHz, CDCl_3_): δ (ppm) = 5.34–5.33 (1H, bd, *J* = 5.3 Hz, H-6); 3.64 (3H, s, COCH_3_); 3.55–3.48 (1H, m, H-3); 2.42 (1H, dq, *J* = 10.4 and 6.8 Hz, H-20); 2.31–2.19 (2H, m, H-4); 2.00–1.19 (2H, m, H-7 and H-12), 1.86–1.82 (2H, m, H-2 and H-1), 1.18 (3H, d, *J* = 6.9 Hz, H-21); 1.00 (3H, s, H-19); 1.00–0.91 (1H, m, H-9); 0.69 (3H, s, H-18). ^13^C NMR (100.6 MHz, CDCl_3_): δ (ppm) = 177.36 (COCH_3_); 140.71 (C-5); 121.51 (C-6); 71.70 (C-3); 56.29 (C-14); 52.83 (C-17); 51.31 (OCH_3_); 50.01 (C-9); 42.44 (C-20); 42.37 (C-13); 42.22 (C-4); 39.50 (C-12); 37.20 (C-1); 36.45 (C-10); 31.85 (C-8); 31.77 (C-7); 31.58 (C-2); 27.12 (C-16); 24.29 (C-15); 20.98 (C-11); 19.35 (C-19); 17.08 (C-21); 11.98 (C-18).

#### 3.2.3. Methyl (20S)-3β-(4-Toluennsulfonyloxy)-pregn-5-ene-20-carboxylate (**14**)

A solution of **13** (1.04 g; 2.88 mmol) in pyridine (15 mL), was treated with an excess of tosyl chloride (1.07 g; 5.61 mmol) and a catalytic amount of DMAP (0.06 g; 0.49 mmol). The mixture was stirred at 45 °C overnight (15 h). The work up of the reaction was with CH_2_Cl_2_ and 5% HCl until the aqueous layer had pH = 6, and then the aqueous phase was extracted with AcOEt (2 × 30 mL), dried with Mg_2_SO_4_, and the solvent evaporated in vacuo. The crude obtained was recrystallized in CH_2_Cl_2_ and ether. Compound **14** (1.18 g, 2.30 mmol, 79.9% yield) was obtained as a colorless solid (m.p. = 127.3–128.1 °C (140–142 °C [47]). IR_νmax_ (cm^−1^): 2944 (C=C-H); 2902 (CH_3_-); 2852 (CH_2_-); 1734 (C=O); 1458 (CH_2_-); 1363 (CH_3_-); 1257 and 1053 (C-O).^1^H NMR (400.1 MHz, CDCl_3_): δ (ppm) = 7.78 (2H, bd, *J* = 8.3 Hz, H-2′ and H-6′); 7.31 (2H, bd, *J* = 8.3 Hz, H-3’ and H-5′); 5.28 (1H, bd, *J* = 5.3 Hz, H-6); 4.34–4.26 (1H, m, H-3); 3.62 (3H, s, COCH_3_); 2.43 (3H, s, Ar-CH_3_); 2.45–2.36 (2H, m, H-4 and H-20); 2.25 (1H, ddd, *J* = 13.2, 5.4 and 1.7 Hz, H-4); 1.96–1.89 (2H, m, H-1 and H-12); 1.81–1.77 (2H, m, H-2 and H-7); 1.73–1.63 (2H, m, H-16 and H-2), 1.60–1.51 (2H, m, H-15 and H-17), 1.16 (3H, d, *J* = 6.9 Hz, H-21); 0.95 (3H, s, H-19); 0.89 (1H, ddd, *J* = 11.2; 11.2 and 5.4 Hz, H-9); 0.66 (3H, s, H-18). ^13^C NMR (100.6 MHz, CDCl_3_): δ (ppm) = 177.25 (COCH_3_); 144.37 (C1′); 138.77 (C-5); 134.58 (C4′); 129.69 (C3′ and C5′); 127.57 (C2′ and C6′); 123.30 (C-6); 82.22 (C-3); 56.14 (C-14); 52.75 (C-17); 51.28 (OCH_3_); 49.75 (C-9); 42.36 (C-20); 42.30 (C-13); 39.34 (C-12); 38.77 (C-4); 36.79 (C-7); 36.26 (C-10); 31.67 (C-1); 31.66 (C-8); 28.53 (C-2); 27.04 (C-16); 24.21 (C-15); 21.58 (Ar-CH_3_); 20.84 (C-11); 19.07 (C-19); 17.04 (C-21); 11.92 (C-18).

#### 3.2.4. Methyl (20S)-6β-Hydroxy-3α,5-cyclo-5α-pregnane-20-carboxylate (**15**)

Tosylate **14** (1.01 g, 1.96 mmol) and potassium acetate (2.26 g, 23.03 mmol) were dissolved in a mixture of acetone/water (40 mL/10 mL) and heated at reflux for 6 h. The end of the reaction was verified via TLC, and the mixture was taken to dryness under reduced pressure. The product was extracted with CH_2_Cl_2_ (30 mL) and water (3 × 20 mL) and dried with MgSO_4_, and the solvent was evaporated in vacuo. The crude product was chromatographed on a column of silica gel (Hexane–ethyl acetate, 85:15) [47]. Compound **15** (0.47 g, 1.31 mmol, 66.6% yield) was obtained as a colorless solid (m.p. = 125.8–127.0 °C). IR_νmax_ (cm^−1^): 3489–3442 (O-H); 2934 (CH_3_-); 2869 (CH_2_-); 2851 (CH_2_-); 1739 (C=O); 1458 (CH_2_-); 1372 (CH_3_-); 1253 and 1057 (C-O). ^1^H NMR (400.1 MHz, CDCl_3_): δ (ppm) = 3.64 (3H, s, COCH_3_); 3.25 (1H, dd, *J* = 2.8 Hz, H-6); 2.47–2.39 (1H, m, H-20); 1.92 (1H, dt, *J* = 12.3 and 3.3 Hz, H-12); 1.18 (3H, d, *J* = 6.9, H-21); 1.05 (3H, s, H-19); 0.90–0.81 (2H, m, H-1 and H-9); 0.73 (3H, s, H-18); 0.52 (1H, dd, *J* = 8.4 and 4.2 Hz, H-4*_gem_*); 0.29 (1H, dd, *J* = 8.4 and 4.8 Hz, H-4*_trans/gem_*). ^13^C NMR (100.6 MHz, CDCl_3_): δ (ppm)= 177.41 (COCH_3_); 73.65 (C-6); 56.02 (C-14); 53.01 (C-17); 51.29 (OCH_3_); 47.58 (C-9); 42.86 (C-10); 42.79 (C-13); 42.41 (C-20); 39.91 (C-12); 38.84 (C-5); 37.04 (C-7); 33.18 (C-1); 29.86 (C-8); 27.19 (C-16); 24.97 (C-2); 24.22 (C-3); 24.22 (C-15); 22.60 (C-11); 20.18 (C-19); 17.09 (C-21); 12.33 (C-18); 11.56 (C-4).

#### 3.2.5. Methyl (20S)-6-Oxo-3α,5-cyclo-5α-pregnane-20-carboxylate (**16**)

Compound **15** (0.46 g, 1.28 mmol) dissolved in a mixture of acetone/CH_2_Cl_2_ (60 mL/12 mL) was treated with the Jones Reagent (1.5 mL) [47] at 5 °C until the orange color was permanent. The reaction mixture was slowly stirred for 2 h and the end of the reaction was verified via TLC. The excess reagent was destroyed by the addition of methanol. The solvent was evaporated under reduced pressure. Another 30 mL of CH_2_Cl_2_ was added, and the organic layer was washed with a saturated solution of NaCl (3 × 10 mL) and water (2 × 10 mL). The organic layer was dried over MgSO_4_ and filtered; then, the solvent was evaporated under reduced pressure, and the crude was chromatographed on silica gel with EtOAc/cyclohexane (85:15). Compound **16** (0.37 g, 1.04 mmol, 81.25% yield) was obtained as a colorless solid (m.p. = 111.0–112.9 °C (112.8–113.0 °C) [1]). IR_νmax_ (cm^−1^): 2945 (CH_3_-); 2871 (CH_2_-); 1736 (C=O); 1687 (C=O); 1458 (CH_2_-); 1368 (CH_3_-); 1257 and 1052 (C-O). ^1^H NMR (400.1 MHz, CDCl_3_): δ (ppm) = 3.64 (3H, s, COCH_3_); 2.47–2.37 (2H, m, H-20 and H-7); 1.97 (1H, dt, *J* = 12.5 and 3.3 Hz, H-12); 1.19 (3H, d, *J* = 6.7 Hz, H-21); 0.99 (3H, s, H-19); 1.03–0.85 (1H, m, H-1); 0.72 (3H, s, H-18); 0.72–0.70 (1H, m, H-4). ^13^C NMR (100.6 MHz, CDCl_3_): δ (ppm) = 209.35 (C-6); 177.15 (COCH_3_); 56.49 (C-14); 52.74 (C-17); 51.84 (OCH_3_); 46.67 (C-10); 46.25 (C-5); 45.97 (C-9); 44.64 (C-7); 42.72 (C-13); 42.32 (C-20); 39.42 (C-12); 35.31 (C-3); 34.71 (C-8); 33.42 (C-1); 27.03 (C-16); 25.84 (C-2); 24.04 (C-15); 22.74 (C-11); 19.63 (C-19); 17.06 (C-21); 12.15 (C-18); 11.63 (C-4).

#### 3.2.6. Methyl (20S)-6-Oxo-5α-pregn-2-ene-20-carboxylate (**17**)

To a solution of cyclopropane derivative **16** (0.31 g; 0.87 mmol) in *N*,*N*-dimethylacetamide (10 mL) was added pyridinium tosylate (0.031 g; 0.12 mmol) and lithium bromide (0.031 g; 0.36 mmol). The reaction mixture was heated at 160 °C under nitrogen for 3 h. Then, additional portions of pyridinium tosylate (0.062 g, 0.25 mmol) and lithium bromide (0.062 mg, 7.14 mmol) were added to the reaction mixture, and heating continued at 160 °C for another 90 min. After cooling, the mixture was poured into water (10 mL), and the product was extracted with ether (25 mL) and water (3 × 10mL). The organic phase was dried with MgSO_4_ and the solvent evaporated in vacuo. The crude product (0.4099 g) was chromatographed on a column of silica gel (Hexane–ethyl acetate, 9:1). Compound **17** (0.2634 g, 0.74 mmol, 85.1% yield) was obtained as a colorless solid (m.p. = 138.5–139.9 °C (149–150 °C [44]). IR_νmax_ (cm^−1^): 3023 (C=C-H); 2969 and 2903 (CH_3_-); 2871 and 2841 (CH_2_-); 1729 (C=O); 1706 (C=O); 1656 (C=C); 1448 (CH_2_-); 1367 (CH_3_-); 1279 and 1023 (C-O). ^1^H NMR (400.1 MHz, CDCl_3_): δ (ppm) = 5.69–5.65 (1H, m, H-3); 5.58–5.53 (1H, m, H-2); 3.63 (3H, s, COCH_3_); 2.46–2.38 (1H, m, H-20); 2.36–2.31 (2H, m, H-7 and H-9); 2.28–2.20 (1H, m, H-1); 1.19 (3H, d, *J* = 6.8 Hz, H-21); 1.16–1.05 (1H, m, H-15); 0.70 (3H, s, H-19); 0.68 (3H, s, H-18). ^13^C NMR (100.6 MHz, CDCl_3_): δ (ppm) = 211.70 (C-6); 177.15 (COCH_3_); 124.92 (C-3); 124.42 (C-2); 56.26 (C-14); 53.77 (C-9); 53.27 (C-5); 52.76 (C-17); 51.35 (OCH_3_); 46.85 (C-7); 42.82 (C-13); 42.30 (C-20); 39.96 (C-10); 39.28 (C-12); 39.21 (C-4); 37.59 (C-8); 26.92 (C-16); 23.95 (C-15); 21.66 (C-1); 21.01 (C-11); 17.06 (C-21); 13.46 (C-19); 12.07 (C-18).

#### 3.2.7. Methyl (20S)-6,6-Ethylenedioxy-5α-pregn-2-ene-20-carboxylate (**18**)

Ethylene glycol (1.68 mL, 30.0 mmol) and TsOH (0.064 g, 0.34 mmol) were added sequentially to a solution of **17** (0.5172 g, 1.4466 mmol) in C_6_H_6_ (50 mL). The solution was refluxed for 2.5 h, using a Dean–Stark apparatus. The solution was cooled, quenched with saturated aqueous NaHCO_3_ (70 mL), and extracted with AcOEt (3 × 30 mL). The combined organic extracts were dried (MgSO_4_) and evaporated the solvent in vacuo [48]**.** Compound **18** (0.5747 g, 99.0%) was obtained as a colorless solid (m.p. = 149.8–152.5 °C. IR_νmax_ (cm^−1^): 3020 (C=C-H); 2938 (CH_3_-); 2872 (CH_2_-); 1737 (C=O); 1458 (CH_2_-); 1380 (CH_3_-); 1276 and 1042 (C-O). ^1^H NMR (400.1 MHz, CDCl_3_): δ (ppm) = 5.60–5.57 (1H, m, H-3); 5.48–5.44 (1H, m, H-2); 3.91–3.80 (3H, m, dioxolane); 3.73–3.68 (1H, m, dioxolane); 3.57 (3H, s, COCH_3_); 2.39–2.31 (1H, m, H-20); 1.70 (1H, dd *J* = 12.8 and 3.5 Hz, H-7α); 1.12 (3H, d, *J* = 6.8 Hz, H-21); 0.95 (1H, dd, *J* = 12.8 and 12.8 Hz, H-7β); 0.80 (3H, s, H-19); 0.72 (1H, td, *J* = 11.7 and 4.4 Hz, H-9); 0.63 (3H, s, H-18). ^13^C NMR (100.6 MHz, CDCl_3_): δ (ppm) = 177.02 (COCH_3_); 125.49 (C-3); 124.54 (C-2); 109.70 (C-6); 65.38 (dioxolane); 63.90 (dioxolane); 55.36 (C-14); 53.22 (C-9); 52.74 (C-17); 51.10 (OCH_3_); 47.87 (C-5); 42.42 (C-13); 42.25 (C-20); 41.04 (C-1); 40.97 (C-7); 39.37 (C-12); 35.70 (C-10); 33.16 (C-8); 26.87 (C-16); 24.03 (C-15); 21.23 (C-4); 20.64 (C-11); 16.91 (C-21); 13.39 (C-19); 11.96 (C-18).

#### 3.2.8. 22-Hydroxy-5α-cholan-2-ene-23,24-dinor-6-one (**19**)

To a solution of ester **18** (1.0 g, 2.48 mmol) in THF (65 mL) was added a stirred suspension of LiAlH_4_ (22 mL, 2M) in THF. The mixture was stirred at room temperature for 90 min. Subsequently, it was treated with cold AcOEt (50 mL) and HCl solution (5%) (30 mL) and stirred for 1 h. The organic layer was extracted with cold AcOEt and washed with water (3 × 30 mL). The organic layer was dried (Na_2_SO_4_) and evaporated. The residue was chromatographed on SiO_2_ with hexane–EtOAc (7:3) to give compound **19** (0.75 g, 2.27 mmol, 91.5% yield), which was obtained as a colorless solid (m.p. = 169.3–172.5 °C) [48]. IR_νmax_ (cm^−1^): 3510 (O-H); 3022 (C=C-H); 2964 and 3940 (CH_3_-); 3905 and 2829 (CH_2_-); 1693 (C=O); 1659 (C=C); 1457 (CH_2_-); 1379 (CH_3_-); 1252 and 1055 (C-O). ^1^H NMR (400.1 MHz, CDCl_3_): δ (ppm) = 5.70–5.66 (1H, m, H-3); 5.58–5.54 (1H, m, H-2); 3.63 (1H, dd, *J* = 10.5 and 3.2 Hz, H-22a); 3.37 (1H, dd, *J* = 10.5 and 6.8 Hz, H-22b); 2.37–2.33 (2H, m, H-7 and H-5); 2.29–2.20 (1H, m, H-4); 1.87–1.77 (1H, m, H-16); 1.76–1.69 (1H, m, H-8); 1.05 (3H, d, *J* = 6.6 Hz, H-21); 0.70 (3H, s, H-19); 0.69 (3H, s, H-18). ^13^C NMR (100.6 MHz, CDCl_3_): δ (ppm) = 211.96 (C-6); 124.91 (C-3); 124.47 (C-2); 67.80 (C-22); 56.44 (C-14); 53.80 (C-5); 53.33 (C-9); 52.31 (C-17); 46.94 (C-7); 42.86 (C-13); 39.99 (C-10); 39.31 (C-12); 39.29 (C-1); 38.59 (C-20); 37.68 (C-8); 27.48 (C-16); 24.01 (C-15); 21.68 (C-4); 21.06 (C-11); 16.70 (C-21); 13.47 (C-19); 11.97 (C-18).

#### 3.2.9. 2α,3α,22-Thrihydroxy-5α-cholan-23,24-dinor-6-one (**8**)

Olefin **19** (0.50 g, 1.51 mmol); DHQD-CLB (0.18 g, 0.39 mmol); CH_3_SO_2_NH_2_ (0.26 g, 2.73 mmol); K_2_CO_3_ (1.15 g, 8.32 mmol); K_3_[Fe(CN)_6_] (2.89 g, 8.78 mmol); and OsO_4_ (0.82 mL, 0.16 mmol) were mixed in *t*-butanol (20 mL). The reaction mixture was stirred at room temperature for 36 h. A saturated solution of sodium sulfite (Na_2_SO_3_) was then added. After an additional 30 min of stirring, the reaction mixture was diluted with ethyl acetate (30 mL) and extracted with water (2 × 20 mL). The combined organic fractions were dried over anhydrous magnesium sulfate and evaporated under reduced pressure. Column chromatography on silica gel with Hexane/EtOAc/MeOH mixtures of increasing polarity (6:4:0 → 4.8:4.8:0.4) gave the desired compound **8** (0.39 g, 1.07 mmol, 70.86% yield), which was obtained as a colorless solid (m.p. = 216.7–221.2 °C). IR_νmax_ (cm^−1^): 3472 (O-H); 2977 and 2949 (CH_3_-); 2902 and 2864 (CH_2_-); 1719 (C=O); 1584 (C=C); 1470 (CH_2_-); 1393 (CH_3_-); 1106 and 1055 (C-O). ^1^H NMR (400.1 MHz, MeOD): δ (ppm) = 3.95 (1H, d, *J* = 2.8 Hz, H-3); 3.66 (1H, ddd, *J* = 11.7; 4.7 and 3.1 Hz, H-2); 3.57 (1H, dd, *J* = 10.7 and 3.1 Hz, H-22a); 3.26 (1H, dd, *J* = 10.7 and 7.1 Hz, H-22b); 2.73 (1H, dd, *J* = 12.2 and 3.4 Hz, H-5); 2.21 (1H, dd, *J* = 13.1 and 4.8 Hz, H-7α); 2.14–2.07 (2H, m, H-7, and H-12); 1.94–1.86 (1H, m, H-16); 1.18–1.10 (1H, m, H-15); 1.05 (3H, d, *J* = 6.5 Hz, H-21); 0.77 (3H, s, H-19); 0.73 (3H, s, H-18). ^13^C NMR (100.6 MHz, MeOD): δ (ppm) = 215.11 (C-6); 69.58 (C-3); 69.23 (C-2); 67.96 (C-22); 57.73 (C-14); 55.19 (C-9); 53.88 (C-17); 52.18 (C-5); 47.63 (C-7); 44.30 (C-13); 43.72 (C-10); 41.10 (C-1); 40.76 (C-12); 40.24 (C-20); 39.27 (C-8); 28.73 (C-16); 27.95 (C-4); 25.19 (C-15); 22.43 (C-11); 17.43 (C-21); 13.98 (C-19); 12.56 (C-18). HRMS-ESI (positive mode): *m*/*z* calculated for C_22_H_36_O_4_: 365.2686 [M + H]^+^ and found 365.2692 [M + H]^+^ (mass error ∆m = 1.5441 ppm) (see Figure S71, Supplementary Material).

#### 3.2.10. General Procedure for Synthesis of 5α-Cholan-6-oxo-2-ene-23,24-dinor-22-(substituted)-benzoate-22-yl (**20**–**22**)

General procedure: Compound **19** was dissolved in DCM (20 mL) and pyridine (0.3 mL). Later, DMAP (15 mg) and *p*-PhCOCl were added with slow stirring at room temperature. The end of the reaction was verified via TLC (3 h), the solvent volume was reduced to about 10 mL, and then EtOAc (20 mL) was added. The organic layer was washed with 5% KHSO_4_ (2 × 5 mL) and water (2 × 10 mL), dried over Na_2_SO_4_, and filtered. The solvent was evaporated under reduced pressure. The crude was redissolved in DCM (5 mL) and chromatographed on silica gel with PE/EtOAc mixtures of increasing polarity (9:1 → 4:6).

#### 3.2.11. 5α-Cholan-6-oxo-2-ene-23,24-dinor-22-benzoate-22-yl (**20**)

Compound **20** was synthesized according to the general procedure described in Section 3.2.10 and using the following amounts: compound **19** (0.500 g, 1.51 mmol), DCM (70 mL), Py (1.2 mL), DMAP (50 mg), and PhCOCl (0.83 mL, 3.04 mmol). Compound **20** (0.56 g, 1.29 mmol, 85.4% yield) was obtained as a colorless solid (m.p. = 133.2–134.1 °C). IR_νmax_ (cm^−1^): 3023 (C=C-H); 2942 and 2904 (CH_3_-); 2870 (CH_2_-); 1713 (C=O); 1656 (C=C); 1601 (C=C Ar); 1451 (CH_2_-); 1388 (CH_3_-); 1110 and 1070 (C-O). ^1^H NMR (400.1 MHz, CDCl_3_): δ (ppm) = 8.04 (2H, d, *J* = 8.4 Hz, H-2′ and H-6′); 7.56 (1H, tt, *J* = 7.6 and 1.5 Hz, H-4′); 7.44 (2H, bt, *J* = 7.6 Hz, H-3′ and H-5′); 5.71–5.66 (1H, m, H-3); 5.59–5.55 (1H, m, H-2); 4.31 (1H, dd, *J* = 10.7 and 3.5 Hz, H-22a); 4.06 (1H, dd, *J* = 10.7 and 7.1 Hz, H-22b); 2.38–2.33 (2H, m, H-7 and H-5); 2.30–2.21 (1H, m, H-4); 1.96–1.85 (2H, m, H-16, and H-20); 1.80–1.71 (1H, m, H-8); 1.66–1.55 (2H, m, H-11, and H-15); 1.19–1.08 (1H, m, H-15); 1.13 (3H, d, *J* = 6.7 Hz, H-21); 0.73 (3H, s, H-18); 0.72 (3H, s, H-19). ^13^C NMR (100.6 MHz, CDCl_3_): δ (ppm) = 211.82 (C-6); 166.68 (ArCO_2_-); 134.81 (C-4′); 130.49 (C-1′); 129.48 (C-2′); 128.33 (C-3′); 124.95 (C-3); 124.45 (C-2); 69.83 (C-22); 56.41 (C-14); 53.80 (C-5); 53.33 (C-9); 52.82 (C-17); 46.91 (C-7); 42.99 (C-13); 39.97 (C-10); 39.32 (C-1); 39.32 (C-12); 37.66 (C-8); 35.95 (C-20); 27.53 (C-16); 24.02 (C-15); 21.66 (C-4); 21.08 (C-11); 17.32 (C-21); 13.48 (C-19); 11.99 (C-18).

#### 3.2.12. 5α-Cholan-6-oxo-2-ene-23,24-dinor-22-(2-Fluoro) benzoate-22-yl (**21**)

Compound **21** was synthesized according to the general procedure described in Section 3.2.10 and using the following amounts: compound **19** (0.30 g; 0.90 mmol), CH_2_Cl_2_(25 mL), pyridine (1.0 mL), DMAP (3 mg), and 2-F-PhCOCl (0.4 mL; 3.28 mmol). Compound **21** (0.27 g, 0.59 mmol, 65.0%) was obtained as a colorless solid (p.f. = 124.4–125.6 °C). IR_νmax_ (cm^−1^): 3072 (C=C-H); 2944 (CH_3_-); 2902 y 2869 (CH_2_-); 1723 (C=O); 1710 (C=C); 1612 (C=C); 1392 (CH_3_-); 1304 and 1134 (C-O); 763 (C=C-H). ^1^H NMR (400.1 MHz, CDCl_3_): δ (ppm) = 7.94 (1H, dd, *J* = 7.7 and 1.5 Hz, H-6′); 7.55–7.49 (1H, m, H-4′); 7.21 (1H, t, *J* = 7.7 Hz, H-5′); 7.14 (1H, dd, *J* = 10.5 and 8.9 Hz, H-3′); 5.70–5.67 (1H, m, H-3); 5.59–5.55 (1H, m, H-2); 4.33 (1H, dd, *J* = 10.7 and 3.2 Hz, H-22a); 4.08 (1H, dd, *J* =10.7 and 6.9 Hz, H-22b); 2.38–2,33 (2H, m, H-5, and H-7α); 1.31 (3H, d, *J* = 6.4 Hz, H-21); 0.72 (3H, s, H-18); 0.71 (3H, s, H-19). ^13^C NMR (100.6 MHz, CDCl_3_): δ (ppm) = 211.89 (C-6); 164.71 (d, ^3^*J*_CF_ = 3.4 Hz, ArCO_2_-); 163.98 (d, ^1^*J*_CF_ = 259.4 Hz, C-2′); 134.33 (d, ^3^*J*_CF_ = 8.9 Hz, C-4′); 132.09 (C-6′); 124.95 (C-3); 124.47 (C-2); 123.91 (d, ^4^*J*_CF_ = 3.7 Hz, C-5′); 118.96 (d, ^2^*J*_CF_ = 9.7 Hz, C-1′); 116.97 (d, ^2^*J*_CF_ = 22.5 Hz, C-3′); 70.29 (C-22); 56.41 (C-14); 53.81 (C-5); 53.32 (C-9); 52.57 (C-17); 46.92 (C-7); 42.94 (C-13); 39.98 (C-10); 39.32 (C-1); 39.29 (C-12); 37.67 (C-8); 35.83 (C-20); 27.48 (C-16); 24.00 (C-15); 21.69 (C-4); 21.07 (C-11); 17.24 (C-21); 13.48 (C-19); 11.98 (C-18).

#### 3.2.13. 5α-Cholan-6-oxo-2-ene-23,24-dinor-22-(4-Fluoro)-benzoate-22-yl (**22**)

Compound **22** was synthesized according to the general procedure described in Section 3.2.10 and using the following amounts: compound **19** (0.30 g, 0.91 mmol), DCM (45 mL), Py (0.75 mL), DMAP (50 mg), and 4-F-PhCOCl (0.74 mL, 6.26 mmol). Compound **22** (0.30 g, 0.66 mmol, 72.5% yield) was obtained as a colorless solid (m.p. = 140.3–141.6 °C). IR_νmax_ (cm^−1^): 3026 (C=C-H); 2942 (CH_3_-); 2903 and 2870 (CH_2_-); 1712 (C=O); 1603 (C=C); 1388 (CH_3_-); 1111 and 1090 (C-O). ^1^H NMR (400.1 MHz, CDCl_3_): δ (ppm) = 8.05 (2H, dd, *J* = 8.8 and 5.4 Hz, H-2′ and H-6′); 7.11 (2H, t, *J* = 8.7 Hz, H-3′ and H-5′); 5.71–5.66 (1H, m, H-3); 5.59–5.55 (1H, m, H-2); 4.30 (1H, dd, *J* = 10.7 and 3.4 Hz, H-22a); 4.05 (1H, dd, *J* = 10.7 and 7.2 Hz, H-22b); 2.38–2.33 (2H, m, H-7 and H-5); 2.30–2.21 (1H, m, H-4); 1.94–1.83 (2H, m, H-16 and H-20); 1.80–1.71 (1H, m, H-8); 1.66–1.55 (2H, m, H-11 and H-15); 1.19–1.08 (1H, m, H-15); 1.12 (3H, d, *J* = 6.6 Hz, H-21); 0.73 (3H, s, H-18); 0.71 (3H, s, H-19). ^13^C NMR (100.6 MHz, CDCl_3_): δ (ppm) = 211.80 (C-6); 165.72 (ArCO_2_-); 165.67 (d, ^1^*J*_CF_ = 253.7 Hz, C-4′); 131.99 (d, ^3^*J*_CF_ = 9.1 Hz, C-2′ and C-6′); 126.71 (d, ^4^*J*_CF_ = 2.7 Hz, C-1′); 124.95 (C-3); 124.44 (C-2); 115.47 (d, ^2^*J*_CF_ = 21.9 Hz, C-3′ and C-5′); 69.97 (C-22); 56.40 (C-14); 53.80 (C-5); 53.32 (C-9); 52.81 (C-17); 46.90 (C-7); 42.99 (C-13); 39.97 (C-10); 39.32 (C-1); 39.32 (C-12); 37.65 (C-8); 35.93 (C-20); 27.53 (C-16); 24.01 (C-15); 21.69 (C-4); 21.07 (C-11); 17.30 (C-21); 13.48 (C-19); 11.98 (C-18).

#### 3.2.14. General Procedure for Synthesis of 2α,3α-Dihydroxy-5α-cholan-6-oxo-23,24-dinor-22-(4-substituted)-benzoate-22-yl (9–11) by Sharpless Dihydroxylation [37,50]

Osmium tetroxide (OsO_4_) in *t*-butanol (1 g per 20 mL) was added to a mixture of olefin (**20**–**22**), hydroquinidine 4-chloro-benzoate (DHQD-CLB), methanesulfonamide (CH_3_SO_2_NH_2_), potassium carbonate (K_2_CO_3_), and potassium ferricyanide (K_3_[Fe(CN)_6_]) dissolved in a mixture of *t*-butanol and water (1:1 *v*/*v*). The reaction mixture was stirred at room temperature for 36 h. A saturated solution of sodium sulfite (Na_2_SO_3_) was then added. After additional stirring for 30 min, the reaction mixture was diluted with ethyl acetate (30 mL) and extracted with water (2 × 20 mL). The combined organic fractions were dried over anhydrous magnesium sulfate and evaporated under reduced pressure. Column chromatography on silica gel with Hexane/EtOAc/MeOH mixtures of increasing polarity (6:4:0 → 4.8:4.8:0.4) gave the desired product.

#### 3.2.15. 2α,3α-Dihydroxy-5α-cholan-6-oxo-23,24-dinor-22-benzoate-22-yl (**9**)

Compound **9** was synthesized according to the general procedure described in Section 3.2.14 and using the following amounts: olefin **20** (0.20 g, 0.46 mmol); DHQD-CLB (0.06 g, 0.13 mmol); CH_3_SO_2_NH_2_ (0.099 g, 1.04 mmol); K_2_CO_3_ (0.36 g, 2.61 mmol); K_3_[Fe(CN)_6_] (0.88 g, 2.67 mmol); OsO_4_ (0.25 mL, 0.05 mmol). Compound **9** (0.194 g, 0.414 mmol, 90% yield) was obtained as a colorless solid (m.p. = 177.7–181.6 °C). IR_νmax_ (cm^−1^): 3384 (O-H); 2942 (CH_3_-); 2866 and 2825 (CH_2_-); 1716 (C=O); 1601 (C=C); 1451 (CH_2_-); 1388 (CH_3_-); 1112 and 1070 (C-O). ^1^H NMR (400.1 MHz, CDCl_3_): δ (ppm) = 8.02 (2H, d, *J* = 8.5 Hz, H-2′ and H-6′); 7.54 (1H, bt, *J* = 7.4 Hz, H-4′); 7.42 (2H, t, *J* = 7.7 Hz, H-3′ and H-5′); 4.29 (1H, dd, *J* = 10.7 and 3.4 Hz, H-22a); 4.05–4.01 (2H, m, H-2 and H-22b); 3.72 (1H, dt, *J* = 10.7 and 3.8 Hz, H-3); 2.78 (2H, bs, 2xOH); 2.66 (1H, dd, *J* = 12.6 and 2.9 Hz, H-5); 2.27 (1H, dd, *J* = 13.1 and 4.5 Hz, H-7α); 1.15–1.04 (1H, m, H-15); 1.10 (3H, d, *J* = 6.6 Hz, H-21); 0.73 (3H, s, H-19); 0.69 (3H, s, H-18). ^13^C NMR (100.6 MHz, CDCl_3_): δ (ppm) = 212.21 (C-6); 166.71 (ArCO_2_-); 132.81 (C-4′); 130.32 (C-1′); 129.41 (C-2′ and C-6′); 128.29 (C-3 and C-5′); 69.77 (C-22); 68.24 (C-3); 68.14 (C-2); 56.22 (C-14); 53.53 (C-9); 52.68 (C-17); 50.66 (C-5); 46.59 (C-7); 43.03 (C-13); 42.45 (C-10); 40.01 (C-1); 39.14 (C-12); 37.56 (C-8); 35.83 (C-20); 27.44 (C-16); 26.24 (C-4); 23.94 (C-15); 21.08 (C-11); 17.24 (C-21); 13.46 (C-19); 12.00 (C-18). HRMS-ESI (positive mode): *m*/*z* calculated for C_29_H_40_O_5_: 469.2946 [M + H]^+^ and found 469.2936 [M + H]^+^ (mass error ∆m = 2.6657 ppm) (see Figure S72, Supplementary Material).

#### 3.2.16. 2α,3α-Dihydroxy-5α-cholan-6-oxo-23,24-dinor-22-(2-Fluoro)-benzoate-22-yl (**10**)

Compound **10** was synthesized according to the general procedure described in Section 3.2.14 and using the following amounts: olefin **21** (0.30 g, 0.66 mmol); DHQD-CLB (0.065 g, 0.17 mmol); CH_3_SO_2_NH_2_ (0.14 g, 1.47 mmol); K_2_CO_3_ (0.52 g, 3.76 mmol); K_3_[Fe(CN)_6_] (1.27 g, 3.86 mmol); and OsO_4_ (0.36 mL, 0.07 mmol). Compound **10** (0.26 g, 0.53 mmol, 82.5% yield) was obtained as a colorless solid (m.p. = 222.4–224.4 °C). IR_νmax_ (cm^−1^): 3414 (O-H); 2945 (CH_3_-); 2891 and 2863 (CH_2_-); 1732 (C=O); 1705 (C=O); 1612 (C=C); 1460 (CH_2_-); 1383 (CH_3_-); 1131 and 1080 (C-O); 765 (C=C-H). ^1^H-NMR (400.1 MHz, CDCl_3_): δ (ppm) = 7.96 (1H, dd, *J* = 7.6 and 1.8 Hz, H-6′); 7.57–7.51 (1H, m, H-4′); 7.23 (1H, dd, *J* = 7.6 and 1.0 Hz, H-5′); 7,16 (1H, ddd, *J* = 10.9; 8.3 and 1.0 Hz, H-3′); 4.36 (1H, dd, *J* = 10.7 and 3.0 Hz, H-22a); 4.11–4.07 (2H, m, H-2 and H-22b); 3.83–3.77 (1H, m, H-3); 2.70 (1H, dd, *J* = 12.7 and 2.3 Hz, H-5); 2.32 (1H, dd, *J* = 13.4 and 4.6 Hz, H-7α); 1.49 (3H, d, *J* = 6.5 Hz, H-21); 0.78 (3H, s, H-19); 0.73 (3H, s, H-18). ^13^C-RMN (100.6 MHz, CDCl_3_): δ (ppm) = 211.95 (C-6); 164.73 (d, ^3^*J*_CF_ = 3.4 Hz, ArCO_2_-); 161.93 (d, ^1^*J*_CF_ = 259.6 C-2′); 134.36 (d, ^3^*J*_CF_ = 8.6 Hz, C-4′); 132.08 (C-6′); 123.93 (d, ^4^*J*_CF_ = 4.0 Hz, C-5′); 118.93 (d, ^2^*J*_CF_ = 10.4 Hz, C-1′); 116.98 (d, ^2^*J*_CF_ = 22.3 Hz, C-3′); 70.25 (C-22); 68.40 (C-3); 68.26 (C-2); 56.31 (C-14); 53.64 (C-9); 52.53 (C-17); 50.72 (C-17); 46.67 (C-7); 43.06 (C-13); 42.52 (C-10); 40.17 (C-1); 39.20 (C-12); 37.62 (C-8); 35.80 (C-20); 27.46 (C-16); 26.30 (C-4); 24.00 (C-15); 21.17 (C-11); 17.22 (C-21); 13.54 (C-19); 12.05 (C-18). HRMS-ESI (positive mode): *m*/*z* calculated for C_29_H_39_FO_5_: 487.2854 [M + H]^+^ and found 487.2855 [M + H]^+^ (mass error ∆m = 0.1457 ppm) (see Figure S73, Supplementary Material).

#### 3.2.17. 2α,3α-Dihydroxy-5α-cholan-6-oxo-23,24-dinor-22-(4-Fluoro)-benzoate-22-yl (**11**)

Compound **11** was synthesized according to the general procedure described in Section 3.2.14 and using the following amounts: olefin **22** (0.30 g, 0.66 mmol); DHQD-CLB (0.08 g, 0.17 mmol); CH_3_SO_2_NH_2_ (0.14 g, 1.47 mmol); K_2_CO_3_ (0.52 g, 3.76 mmol); K_3_[Fe(CN)_6_] (1.27 g, 3.86 mmol); and OsO_4_ (0.36 mL, 0.07 mmol). Compound 2α,3α-glycol **11** (0.27 g, 0.56 mmol, 84.9% yield) was obtained as a colorless solid (m.p. = 186.5–187.2 °C). IR_νmax_ (cm^−1^): 3518 and 3407 (O-H); 2969 and 2942 (CH_3_-); 2894 and 2849 (CH_2_-); 1712 (C=O); 1604 (C=C); 1389 (CH_3_-); 1109 and 1059 (C-O). ^1^H NMR (400.1 MHz, CDCl_3_): δ (ppm) = 8.05 (2H, dd, *J* = 9.0 and 5.5 Hz, H-2′ and H-6′); 7.11 (2H, dd, *J* = 9.0 and 8.5 Hz, H-3′ and H-5′); 4.30 (1H, dd, *J* = 10.7 and 3.3 Hz, H-22a); 4.06–4.02 (2H, m, H-2 and H-22b); 3.77 (1H, dt, *J* = 11.5 and 3.9 Hz, H-3); 2.68 (1H, dd, *J* = 12.5 and 3.1 Hz, H-5); 2.30 (1H, dd, *J* = 13.2 and 4.5 Hz, H-7α); 1.18–1.07 (1H, m, H-15); 1.12 (3H, d, *J* = 6.6 Hz, H-21); 0.76 (3H, s, H-19); 0.71 (3H, s, H-18). ^13^C NMR (100.6 MHz, CDCl_3_): δ (ppm) = 211.92 (C-6); 165.77 (ArCO_2_-); 165.70 (d, ^1^*J*_CF_ = 273.7 Hz, C-4′); 132.01 (d, ^3^*J*_CF_ = 9.2 Hz, C-2′ and C6′); 126.69 (d, ^4^*J*_CF_ = 2.8 Hz, C-1′); 115.05 (d, ^2^*J*_CF_ = 22.1 Hz, C-3′ and C5′); 69.96 (C-22); 68.36 (C-3); 68.25 (C-2); 56.32 (C-14); 53.64 (C-9); 52.77 (C-17); 50.69 (C-5); 46.68 (C-7); 43.13 (C-13); 42.53 (C-10); 40.16 (C-1); 39.23 (C-12); 37.63 (C-8); 35.93 (C-20); 27.54 (C-16); 26.27 (C-4); 24.02 (C-15); 21.16 (C-11); 17.30 (C-21); 13.54 (C-19); 12.07 (C-18). HRMS-ESI (positive mode): *m*/*z* calculated for C_29_H_39_FO_5_: 487.2854 [M + H]^+^ and found 487.2851 [M + H]^+^ (mass error ∆m = −2.1117 ppm) (see Figure S74, Supplementary Material).

### 3.3. Biological Activity

#### 3.3.1. Bioactivity in the Rice Lamina Inclination Test (RLIT) of Brassinosteroids Analogs

To evaluate the bioactivity of BR analogs (**9**–**11**), we used RLIT [59] and followed the modified procedure previously described by Díaz et al. [17]. Seeds of a local rice cultivar (*Oryza sativa* L.) of the *Zafiro* variety (provided by INIA-QUILAMAPU, CHILE) were sterilized and then synchronized by soaking in sterile distilled water for 24 h, sown and grown for about 10 days in pots with a substrate, and maintained at 22 °C under a 16 h light/8 h dark photoperiod and 50–60% relative humidity in a plant growth chamber. Uniformly growing rice plants were selected to cut an approximately 8 cm segment containing the second internode of the rice lamina. These segments were then placed in a Petri dish containing sterile distilled water (60 mL) and the respective solutions of each treatment to reach the desired concentration (1 × 10^−7^ and 1 × 10^−6^ M) of each BR analog and brassinolide (APExBIO) used as a positive control. The negative control contained water and the same amount of dimethyl sulfoxide (DMSO) added to BR analogs. They were then left to incubate for 72 h at 25 °C in the dark to finally measure the angle of inclination of the unrolled sheet between the leaf and the sheath (see Figure S75, Supplementary Material). Each treatment consisted of 10 independent replicates; with these data, significant differences between the positive control and the treatments were determined. Mean values with at least a significant difference (*p* < 0.05; Student’s *t*-test) were considered. Images were taken with a Leica EZ4HD stereo microscope with camera software (LAS EZ 3.4 DVD 272, Leica Microsystems, Wertzlar, Germany).

#### 3.3.2. Bean Second Internode Bioassay

The bean second internode test for compounds **8**–**11** was carried out using the procedure reported by Slavikova et al., 2008 [60], with some modifications. Bean seeds (*Phaseolus vulgaris* L., cv. Pinto) were sown and germinated for three days, and then the uniformly germinated seeds were transplanted into pots containing perlite, vermiculite, and a substrate. The pots were kept in a plant growth chamber at 22 °C, with 48 W/m^2^ light and a 16 h/8 h light/dark photoperiod. When the second internode of bean plants was 1–2 mm long (approx. after 7 days), they were treated with each of the tested compounds dissolved in DMSO and water at a final concentration of 1 × 10^−8^ M via application on a small scar generated once the bract was removed from the base of the second internode. At the time of application, a 5 µL drop of each solution was mixed with a 2 µL drop of TWEEN^®^ 20 (AMRESCO^®^) for adhesion.

Control plants were treated with water and TWEEN 20^®^ only. Measurements of the second internode length were made after 5 days. The difference between the length of the second internode of treated and control plants was used as a measure of activity.

### 3.4. Molecular Docking

The crystallographic structure of the protein Brassinosteroid Insensitive 1 (BRI1), in complex with BRI1-Associated Receptor Kinase 1 (BAK1) and the natural ligand (brassinolide) (PDB ID: 4M7E), resolved at 3.60 Å, was retrieved from the Protein Data Bank (http://www.rcsb.org/). The structure was optimized using pdb2pqr.py (Version 3.6.0), implemented on the web server PDB2PQR (http://server.poissonboltzmann.org/pdb2pqr, accessed on 7 August 2023), and utilized the AMBER force field. The protonation state of ionizable groups at pH 8 was assigned using PROPKA 4.5 [61,62]. For each of the analyzed compounds, a molecular model was generated through their SMILES string using UCSF Chimera [63]. Energy minimization of the models was performed by employing Chimera’s default conditions with MMTK and Antechamber parameters [64]. AutoDock Vina 1.1.2 was employed as the docking algorithm, utilizing a grid box of dimensions 20 × 20 × 20 Å, with the center of brassinolide in the crystal structure serving as the center of the docking box. For each analyzed molecular model, five docking runs were conducted, generating ten poses each, with an exhaustiveness of eight. The maximum energy difference between modes was limited to 2 kcal/mol. The docking analyses were inspected based on binding affinity values (kcal/mol) as well as hydrogen, hydrophobic, and electrostatic bonding interactions. Following docking, a comparison was made between the docked ligand and re-docked brassinolide poses. The visualization of the docked poses for their analysis was performed using UCSF Chimera [63] and Discovery Studio Visualizer (BIOVIA, San Diego, CA, USA) [65].

## Figures and Tables

**Scheme 1:**
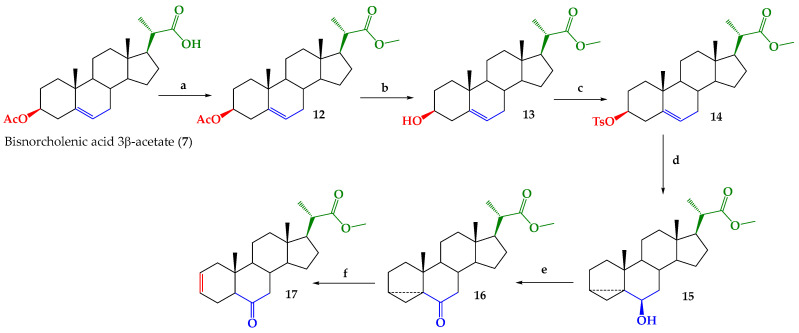
Synthesis of compounds **12**–**17**. Conditions: (**a**) CH_2_N_2_/ether, r.t. 6 h, 99.8%; (**b**) NaHCO_3_/MeOH/H_2_O, reflux, 2 h, 99.1%; (**c**) TsCl/pyridine/DMAP, 45 °C, 15 h, 79.9%; (**d**) KAcO/(CH_3_)_2_CO/H_2_O, reflux, 6 h, 66.6%; (**e**) Jones (CH_3_)_2_CO/CH_2_Cl_2_/, 5 °C, 2 h, 81.3%; (**f**) LiBr/PPTS/CH_3_CON(CH_3_)_2_, 160 °C, 3 h, 85.1%.

**Table 1:** Effect of BRs analogs with 23,24-dinor-5α-Cholane type side chains on lamina inclination of rice seedlings. Brassinolide (**1**) was used as positive control.

Bending Angle between Lamina and Sheaths (Degrees ± Standard Error) ^1^		
Compounds	1 × 10^−7^ M	1 × 10^−6^ M
1	46 ± 8.0 ^a^	89 ± 4.9 ^a^
8	26 ± 6.1 ^c^	16 ± 4.1 ^d^
9	21 ± 2.0 ^c^	10 ± 2.6 ^e^
10	42 ± 0.0 ^a^	24 ± 2.7 ^c^
11	33 ± 2.0 ^b^	36 ± 2.0 ^b^

^1^ These values represent the mean ± standard deviation of two independent experiments with at least ten replicates each. Average angle of negative control: 3 ± 2.6. Letters represent experiments with a significant difference between positive control (**1**) and analog treatments at the 0.05 significance level (Student’s *t*-test).

**Table 2:** Activity in the bean second internode bioassay of brassinolide and BRs **8**–**11** at 1 × 10^−8^ M.

Compounds	Elongation of the Second Internode, mm ± SD
**1**	20.2 ± 0.65 ^b^
**8**	8.5 ± 0.49 ^c^
**9**	20.8 ± 0.26 ^b^
**10**	17.5 ± 0.52 ^b^
**11**	85.0 ± 4.00 ^a^

Letters indicate the level of significance according to Student’s *t*-test; *p* < 0.05.

## Data Availability

Data is contained within the article.

## Supplementary Materials

The following supporting information can be downloaded at: https://www.mdpi.com/article/10.3390/ijms25010419/s1.
