# Supplementary material for: New Brassinosteroid Analogs with 23,24-Dinorcholan Side Chain, and Benzoate Function at C-22: Synthesis, Assessment of Bioactivity on Plant Growth, and Molecular Docking Study
Source: Int J Mol Sci. 2023 Dec 28;25(1):419. doi: 10.3390/ijms25010419 (PMC10778888; doi:10.3390/ijms25010419)
Supplement: Supplementary file 1 [file ijms-25-00419-s001.zip › ijms-2771675-supplementary.pdf]

International Journal of *Molecular Sciences*

MDPI

Article

New Brassinosteroid Analogs with 23,24-Dinorcholan Side Chain, and Benzoate Function at C-22. Synthesis, Assessment of Bioactivity on Plant Growth, and Molecular Docking Study

Vanessa Aitken [1], Katy Diaz [1], Mauricio Soto [1], Andrés F. Olea [2,*], Mauricio A. Cuellar [3,4], Maria Nuñez [1] and Luis Espinoza-Catalán [1,*]

[1] Departamento de Química, Universidad Técnica Federico Santa María, Avenida España 1680, Valparaíso 2340000, Chile; vanessa.aitken.13@sansano.usm.cl (V.A.); katy.diaz@usm.cl (K.D.); mauricio.sotoc@usm.cl (M.S.)

[2] Grupo QBAB, Instituto de Ciencias Aplicadas, Facultad de Ingeniería, Universidad Autónoma de Chile, El Llano Subercaseaux 2801, Santiago, CP 8900000, Chile

[3] Acultad de Farmacia, Escuela de Química y Farmacia, Universidad de Valparaíso, Av. Gran Bretaña 1093, Valparaíso 2340000, Chile; mauricio.cuellar@uv.cl

[4] Centro de Investigación Farmacopea Chilena (CIFAR), Universidad de Valparaíso, Valparaíso 2340000, Chile

* Correspondence: andres.olea@uautonoma.cl (A.F.O.); maria.nunezg@usm.cl (M.N.); luis.espinozac@usm.cl (L.E.-C.); Tel.: +56-32-2654425 (L.E.-C.)

Citation: Aitken, V.; Diaz, K.; Soto, M.; Olea, A.F.; Cuellar, M.A.; Nuñez, M.; Espinoza-Catalán, L. New Brassinosteroid Analogues with 23,24-Dinorcholan Side Chain, and Benzoate Function at C-22. Synthesis, Assessment of Bioactivity on Plant Growth, and Molecular Docking Study. *Int. J. Mol. Sci.* **2024**, *25*, x. https://doi.org/10.3390/xxxxx

Academic Editor: Keimei Oh

Received: 29 November 2023
Revised: 23 December 2023
Accepted: 27 December 2023
Published: date

Copyright: © 2023 by the authors. Submitted for possible open access publication under the terms and conditions of the Creative Commons Attribution (CC BY) license (https://creativecommons.org/licenses/by/4.0/).

Index **pag.**

Figure S1. ^{1}H NMR spectrum compound **12**……………………………………… ……..……...............…………..1
Figure S2. ^{13}C NMR spectrum compound **12**…………………………………… ………….......……..………….2
Figure S3. ^{1}H NMR spectrum compound **13**…………………………….…………………………………..……..…..…3
Figure S4. ^{13}C NMR spectrum compound **13**…………………….…………………………………...……..………4
Figure S5. ^{1}H NMR spectrum compound **14**………….…………………….………………………….…..……......…5
Figure S6. ^{13}C NMR spectrum compound **14**……………………..…………..………………..………………..6
Figure S7. ^{13}C DEPT-135 NMR spectrum compound **14**.…………………………………….……………..……7
Figure S8. 2D HSQC spectrum compound **14**…………….…………...……...…8
Figure S9. 2D HMBC spectrum compound **14**…………………………..……...…9
Figure S10. ^{1}H NMR spectrum compound **15**…………………..…….….…..……..10
Figure S11. ^{13}C NMR spectrum compound **15**…………………………..…...11
Figure S12. ^{13}C DEPT-135 NMR spectrum compound **15**………..………...……..……12
Figure S13. 2D HSQC ed spectrum compound **15**…………………...………...…13
Figure S14. 2D HMBC spectrum compound **15**…………………………………………..……………………14
Figure S15. ^{1}H NMR spectrum compound **16**……………………………………..…………………………15
Figure S16. ^{13}C NMR spectrum compound **16**……………………………………………………….......……16
Figure S17. ^{13}C DEPT-135 NMR spectrum compound **16**…………..………………………………….....…… 17
Figure S18. 2D HSQC ed spectrum compound **16**…………………………………………………..………..18
Figure S19. 2D HMBC spectrum compound **16**……………………………………………………..…...…..…19
Figure S20. ^{1}H NMR spectrum compound **17**…………………………………………………………...…20
Figure S21. ^{13}C NMR spectrum compound **17**…………………………………………………..………..…..21
Figure S22. ^{13}C DEPT-135 NMR spectrum compound **17**…………………………………………...….22
Figure S23. 2D HSQC spectrum compound **17**……..…………………………………………………………23
Figure S24. 2D HMBC spectrum compound **17**……………………………………………………………....…24
Figure S25. ^{1}H NMR spectrum compound **18**…………………………………………………..……....….25
Figure S26. ^{13}C NMR spectrum compound **18**……………………………………….………..………..26
Figure S27. ^{13}C DEPT-135 NMR spectrum compound **18**………………..……………………………….....27
Figure S28. 2D HSQC ed spectrum compound **18**……………………………………..……..…………..28
Figure S29. 2D HMBC spectrum compound **18**……………….…………………………………………...…29
Figure S30. ^{1}H NMR spectrum compound **19**………………………………...…………………………..…..30
Figure S31. ^{13}C NMR spectrum compound **19**…………………………………………………………….……31
Figure S32. ^{13}C DEPT-135 NMR spectrum compound **19**……………………………………………...….32
Figure S33. 2D HSQC ed spectrum compound **19**…………………………………………………….…...33
Figure S34. 2D HMBC spectrum compound **19**……………………………………………………...…..34
Figure S35. ^{1}H NMR spectrum compound **8**………………………….………………………….……..35

Figure S36. ^{13}C NMR spectrum compound **8**……………………………………………………………..36
Figure S37. ^{13}C DEPT-135 NMR spectrum compound **8**…………………………………………………..37
Figure S38. 2D HSQC ed spectrum compound **8**…………………………………………………………38
Figure S39. 2D HMBC spectrum compound **8**……………………………………………………………..39
Figure S40. 1D selective NOESY compound **8**…………………………………………………………..40
Figure S41. ^{1}H NMR spectrum compound **20**…………………………………………………………..41
Figure S42. ^{13}C NMR spectrum compound **20**………………………………………………………..42
Figure S43. ^{13}C DEPT-135 NMR spectrum compound **20**…………………………………………….43
Figure S44. 2D HSQC ed spectrum compound **20**……………………………………………………….44
Figure S45. 2D HMBC spectrum compound **20**…………………………………………………………..45
Figure S46. ^{1}H NMR spectrum compound **21**…………………………………………………………..46
Figure S47. ^{13}C NMR spectrum compound **21**…………………………………………………………..47
Figure S48. ^{13}C DEPT-135 NMR spectrum compound **21**……………………………………………….48
Figure S49. 2D HSQC ed spectrum compound **21**………………………………………………………..49
Figure S50. 2D HMBC spectrum compound **21**…………………………………………………………..50
Figure S51. ^{1}H NMR spectrum compound **22**…………………………………………………………...51
Figure S52. ^{13}C NMR spectrum compound **22**…………………………………………………………52
Figure S53. ^{13}C DEPT-135 NMR spectrum compound **22**……………………………………………...53
Figure S54. 2D HSQC ed spectrum compound **22**…………………………………………………………54
Figure S55. 2D HMBC ed spectrum compound **22**…………………………………………………………55
Figure S56. ^{1}H NMR spectrum compound **9**…………………………………………………………....56
Figure S57. ^{13}C NMR spectrum compound **9**…………………………………………………………57
Figure S58. ^{13}C DEPT-135 NMR spectrum compound **9**……………………………………………….58
Figure S59. 2D HSQC ed spectrum compound **9**…………………………………………………………59
Figure S60. 2D HMBC ed spectrum compound **9**…………………………………………………………60
Figure S61. ^{1}H NMR spectrum compound **10**…………………………………………………………....61
Figure S62. ^{13}C NMR spectrum compound **10**..……………………………………………………….62
Figure S63. ^{13}C DEPT-135 NMR spectrum compound **10**……………………………………………63
Figure S64. 2D HSQC ed spectrum compound **10**……..…………………………………………………64
Figure S65. 2D HMBC ed spectrum compound **10**…………………………………………………….65
Figure S66. ^{1}H NMR spectrum compound **11**…………………………………………………………..66
Figure S67. ^{13}C NMR spectrum compound **11**…………………………………………………………67
Figure S68. ^{13}C DEPT-135 NMR spectrum compound **11**……………………………………………..68
Figure S69. 2D HSQC ed spectrum compound **11**…………………………………………………………69
Figure S70. 2D HMBC ed spectrum compound **11**…………………………………………………………70
Figure S71. HRSM spectra of compounds **8**………..…………………………………………………….71
Figure S72. HRSM spectra of compounds **9**……………………………………………………………71

Figure S73. HRSM spectra of compounds **10**……….………..…………………………………….……….…….72

Figure S74. HRSM spectra of compounds **11**……………………………………………………….….….……………72

Figure S75. Effect of brassinolide and BRs analogs on the Rice Lamina Inclination **1**, **8-11**…..……………..…..…….73

Figure S76. Predicted binding mode of compound **1**, **8-11** ……………………………………………….………………..…75

Table S1. Pose analysis of docked brassinolide and synthetic analogs (**8-11**)…….……………….………………..76

Table S2. Docked ligands-heterodimer protein of **1** and BRs analogs (**8-11**) …………………………………77

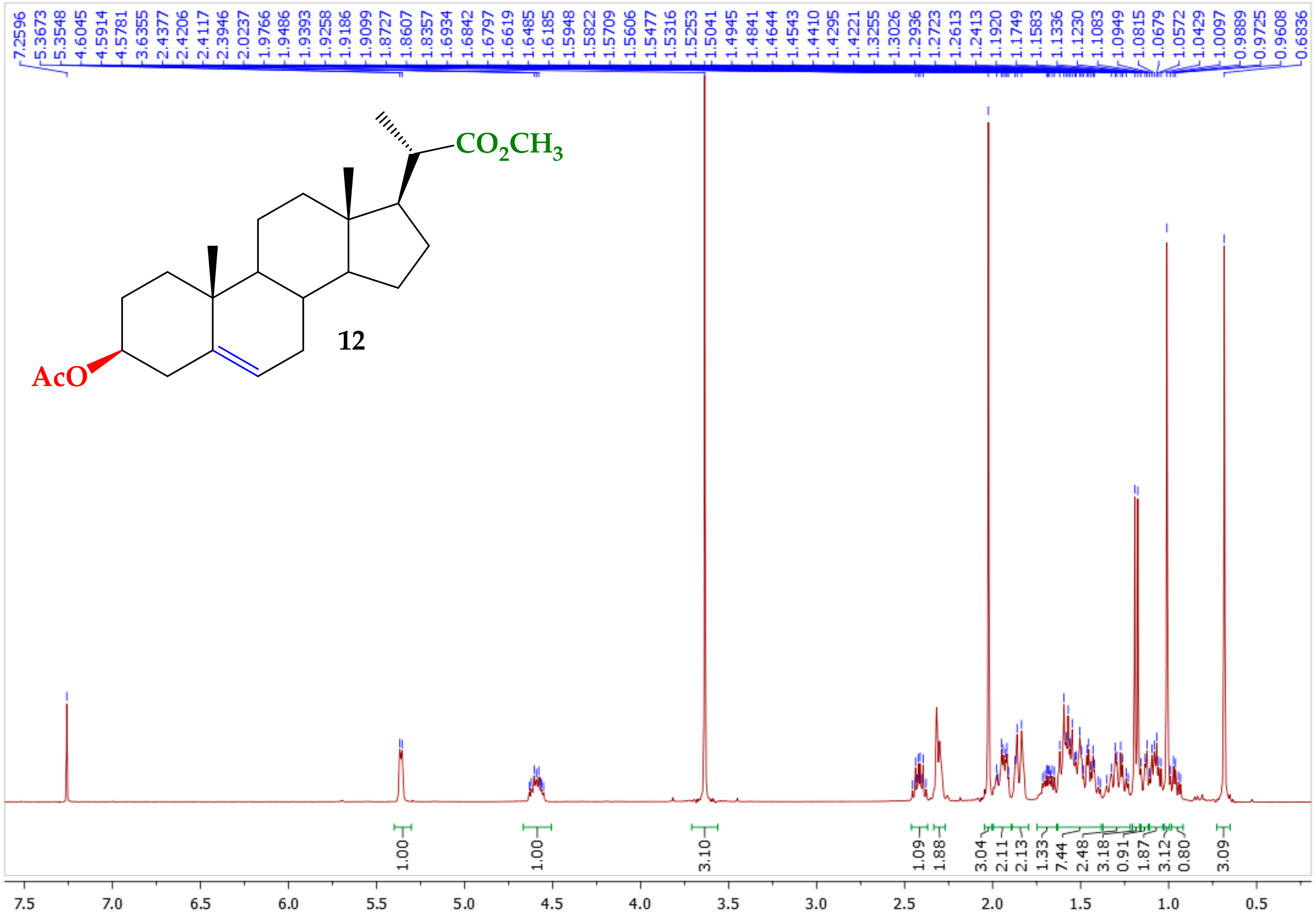

Figure S1. ^{1}H NMR spectrum of Methyl (20S)-3β-Acetoxypregn-5-ene-20-carboxylate (**12**)

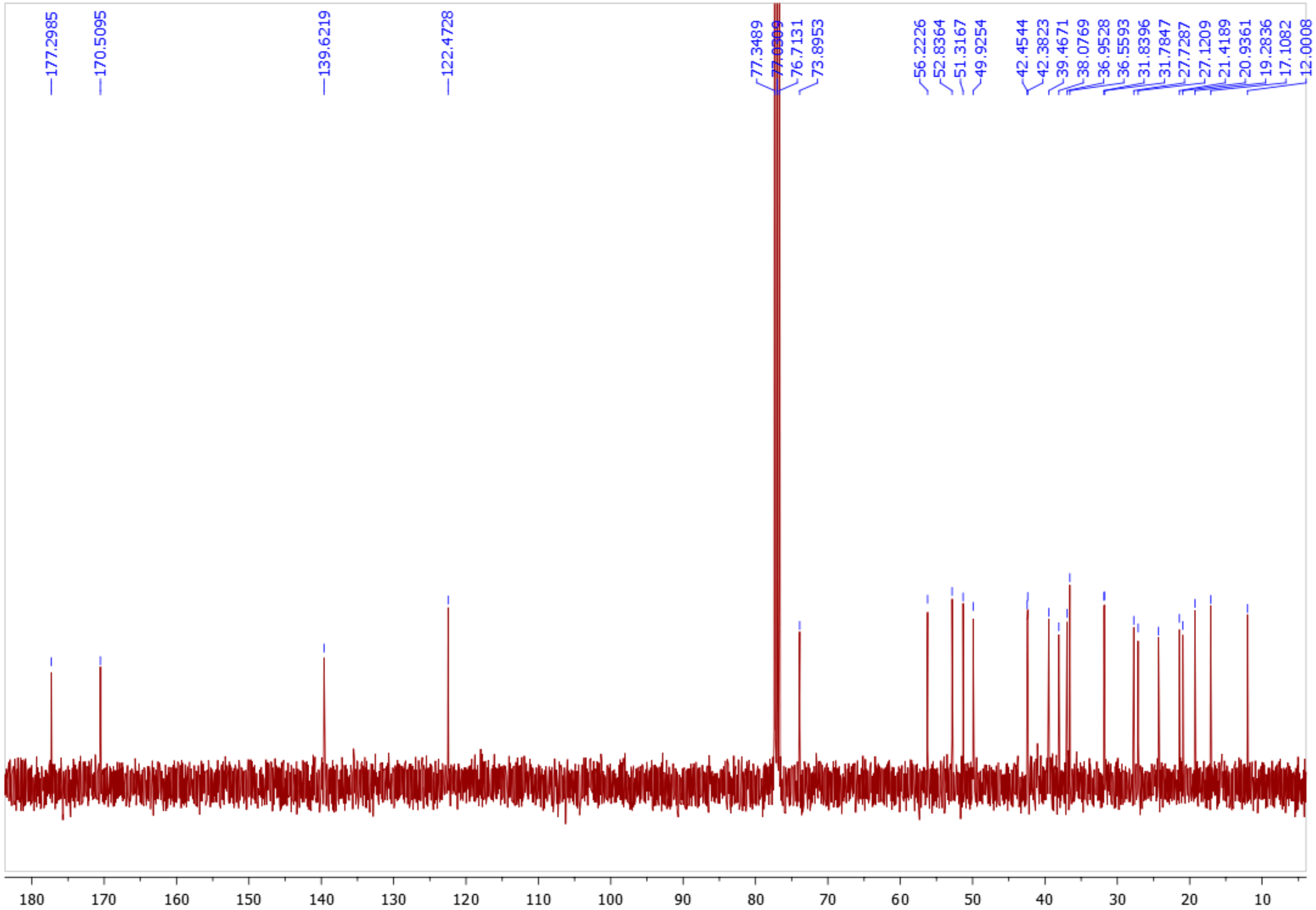

Figure S2. ^{13}C NMR spectrum of Methyl (20S)-3β-Acetoxypregn-5-ene-20-carboxylate (**12**)

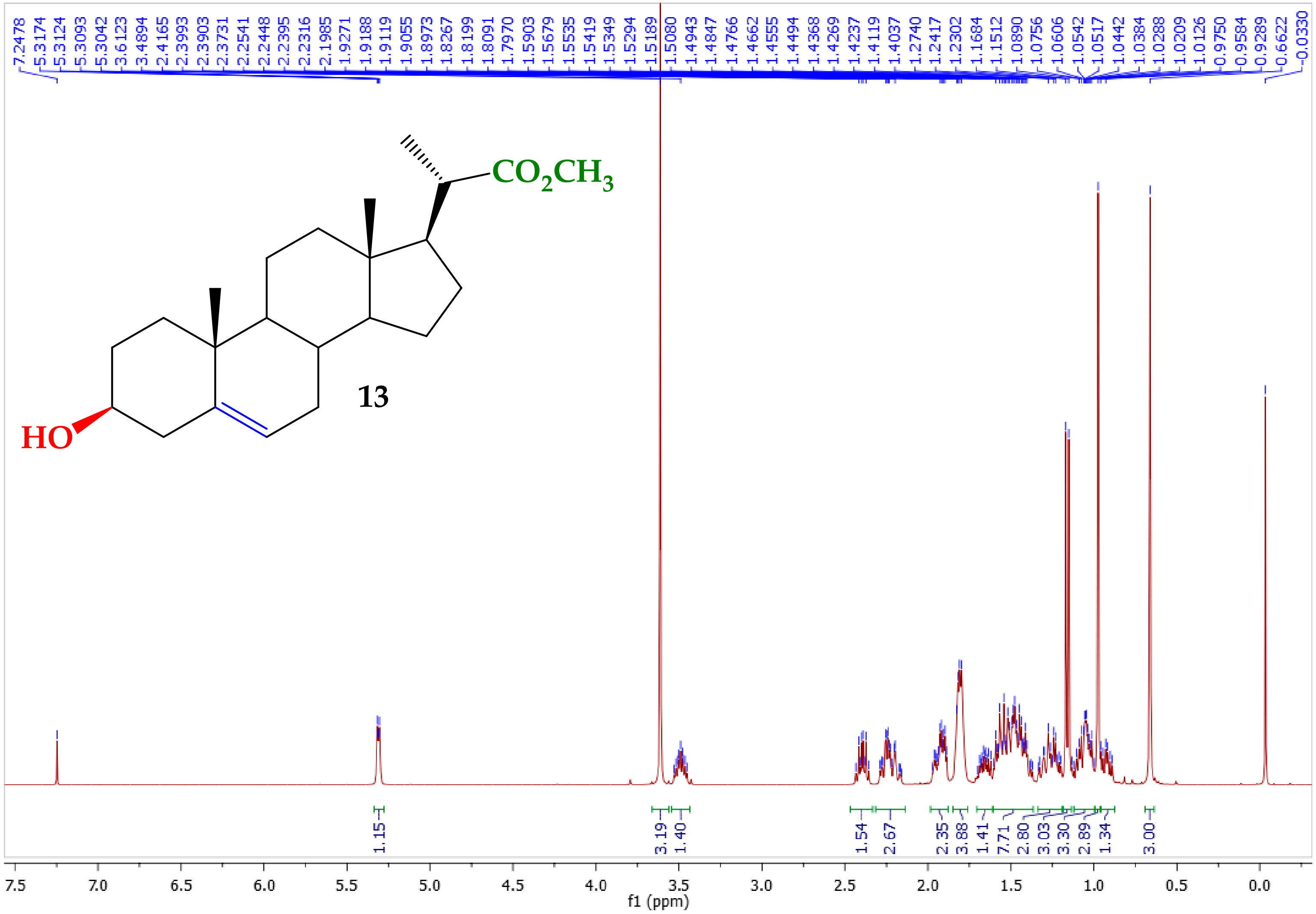

Figure S3. ^{1}H NMR spectrum of Methyl (20S)-3β-hydroxy-pregn-5-ene-20-carboxylate (**13**)

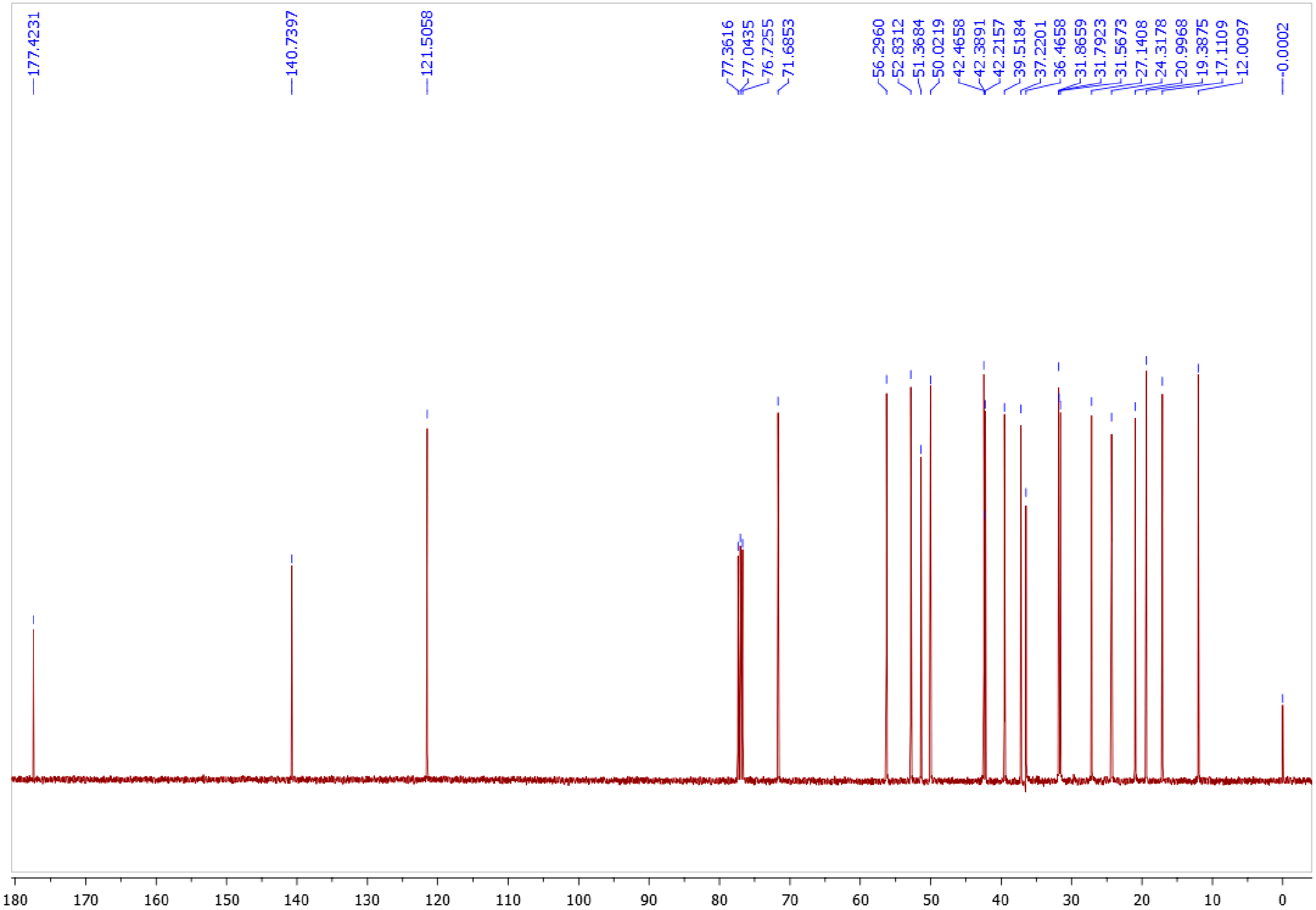

Figure S4. ^{13}C NMR spectrum of Methyl (20S)-3β-hydroxy-pregn-5-ene-20-carboxylate (**13**)

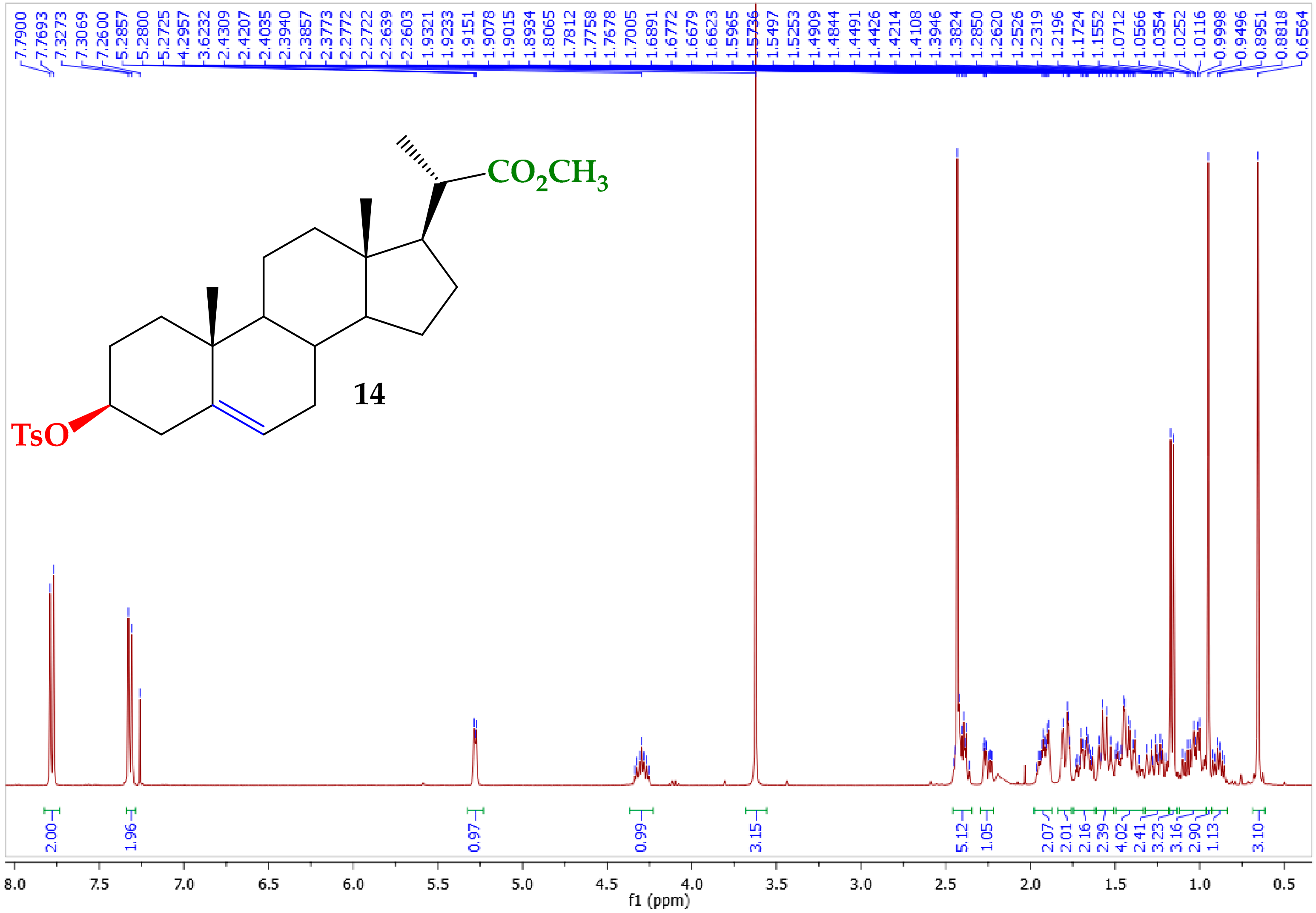

Figure S5. ^{1}H NMR spectrum of Methyl (20S)-3β-(4-toluennsulfonyloxy)-pregn-5-ene-20-carboxylate (**14**)

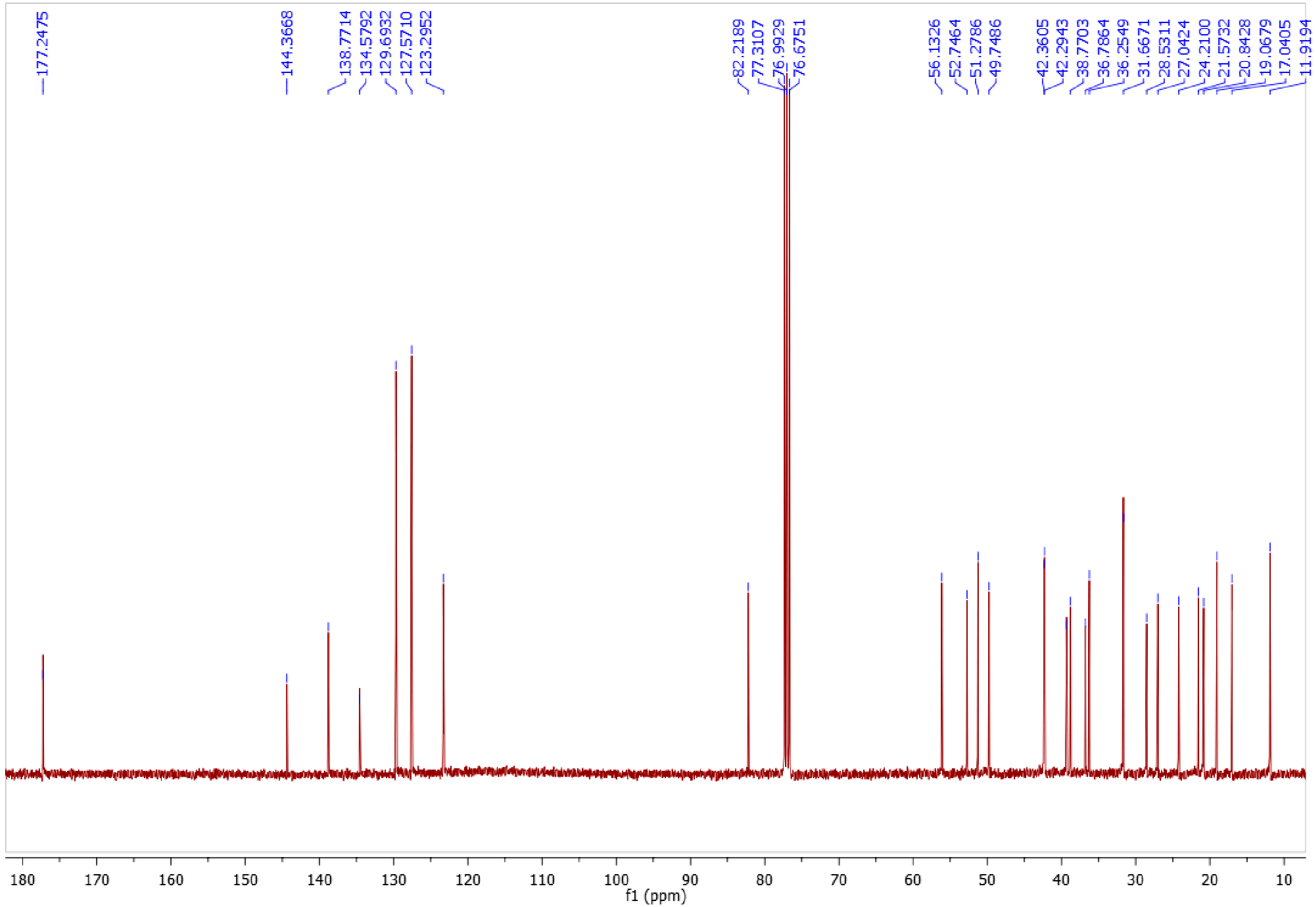

Figure S6. ^{13}C NMR spectrum of Methyl (20S)-3β-(4-toluennsulfonyloxy)-pregn-5-ene-20-carboxylate (**14**)

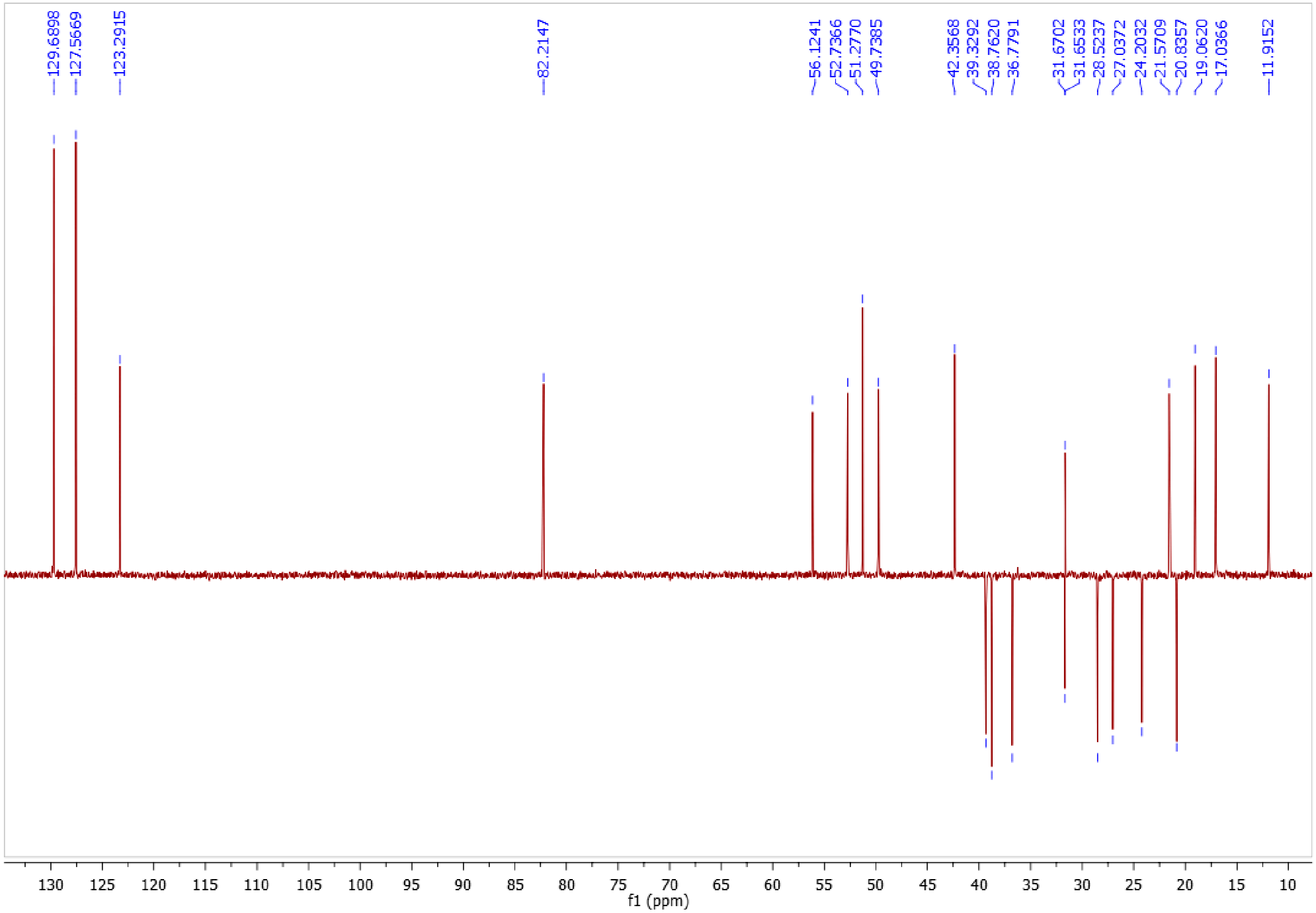

Figure S7. ^{13}C DEPT-135 NMR spectrum of Methyl (20S)-3β-(4-toluennsulfonyloxy)-pregn-5-ene-20-carboxylate (**14**)

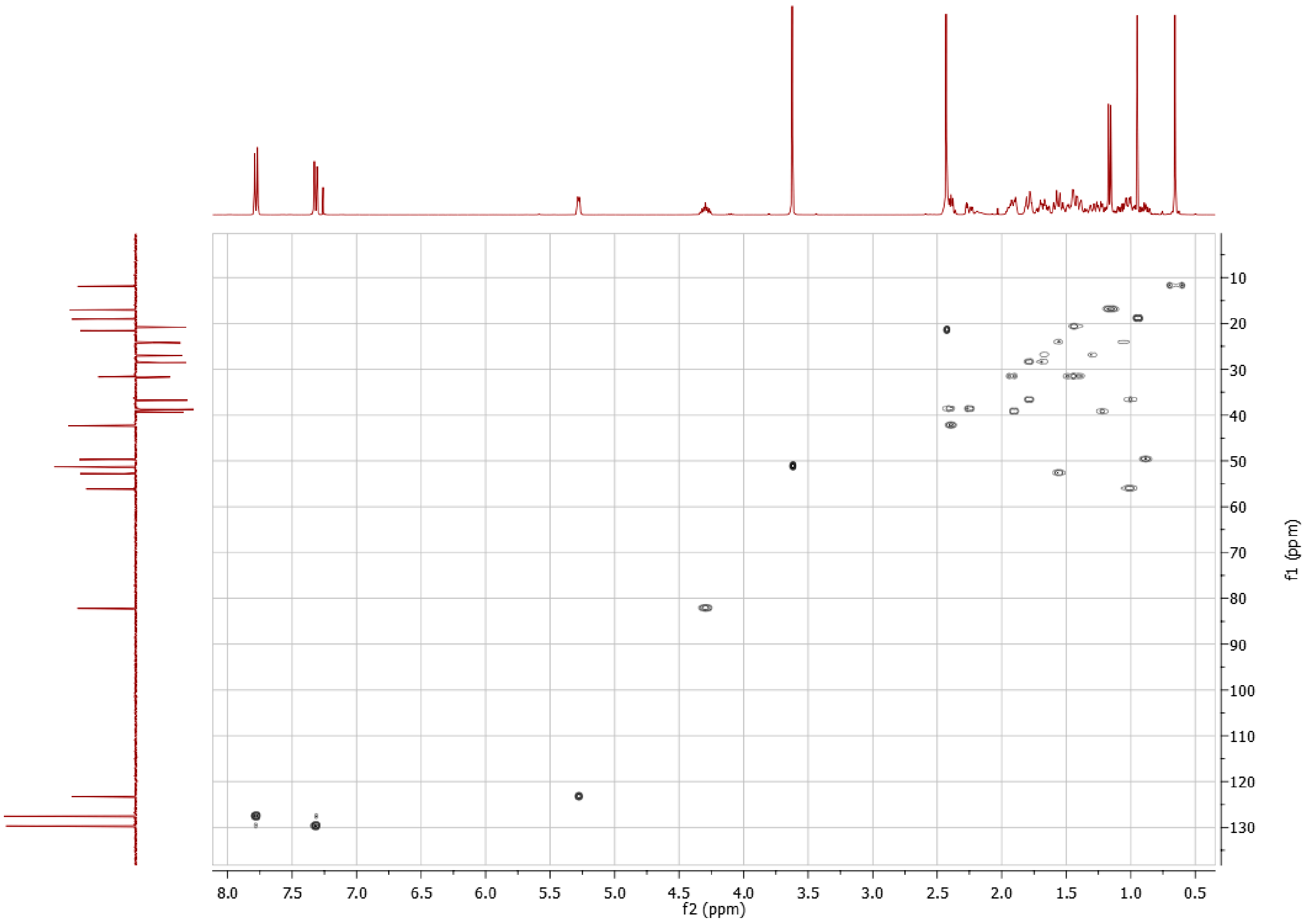

Figure S8. 2D HSQC spectrum of Methyl (20S)-3β-(4-toluennsulfonyloxy)-pregn-5-ene-20-carboxylate (**14**)

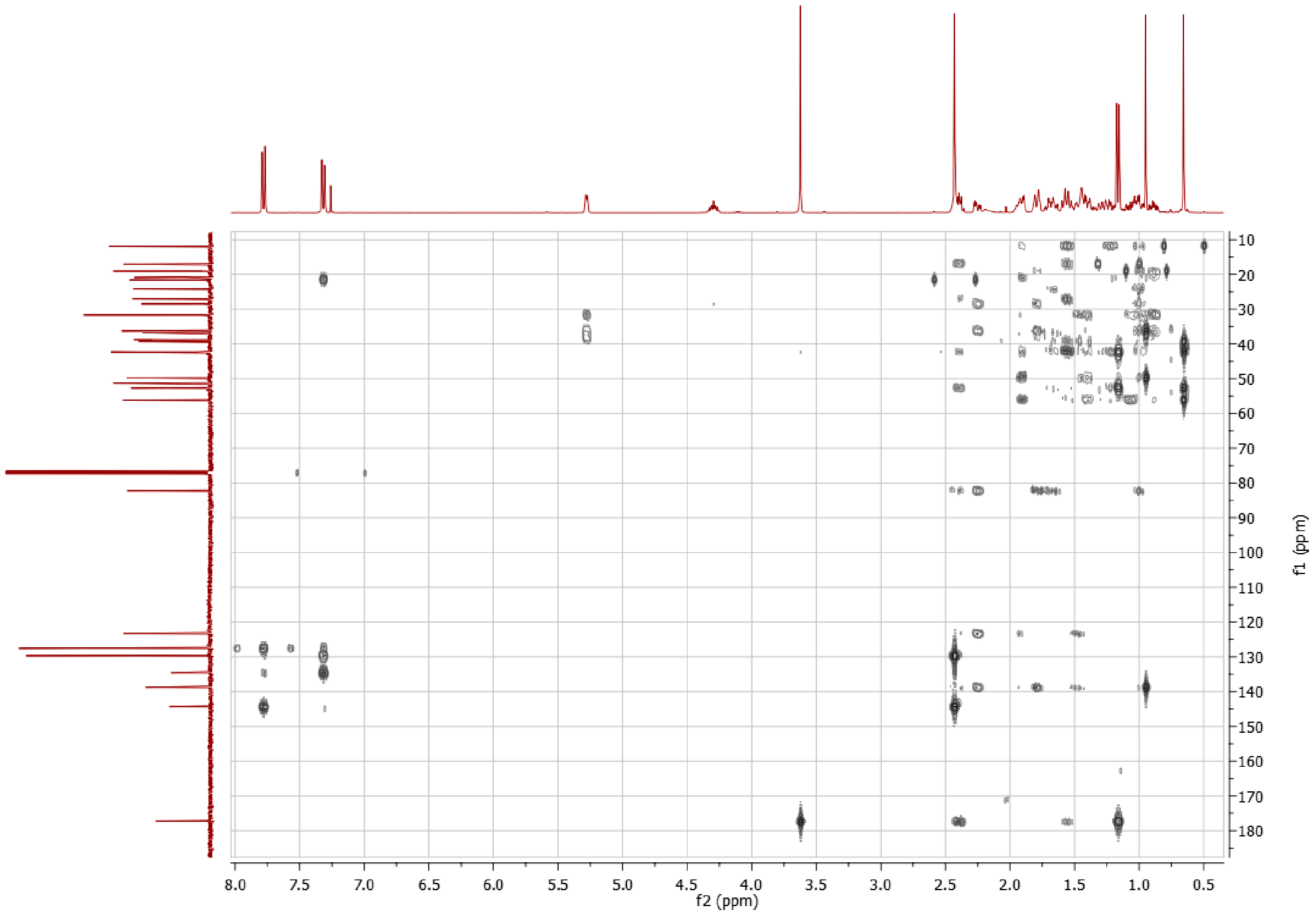

Figure S9. 2D HMBC spectrum of Methyl (20S)-3β-(4-toluennsulfonyloxy)-pregn-5-ene-20-carboxylate (**14**)

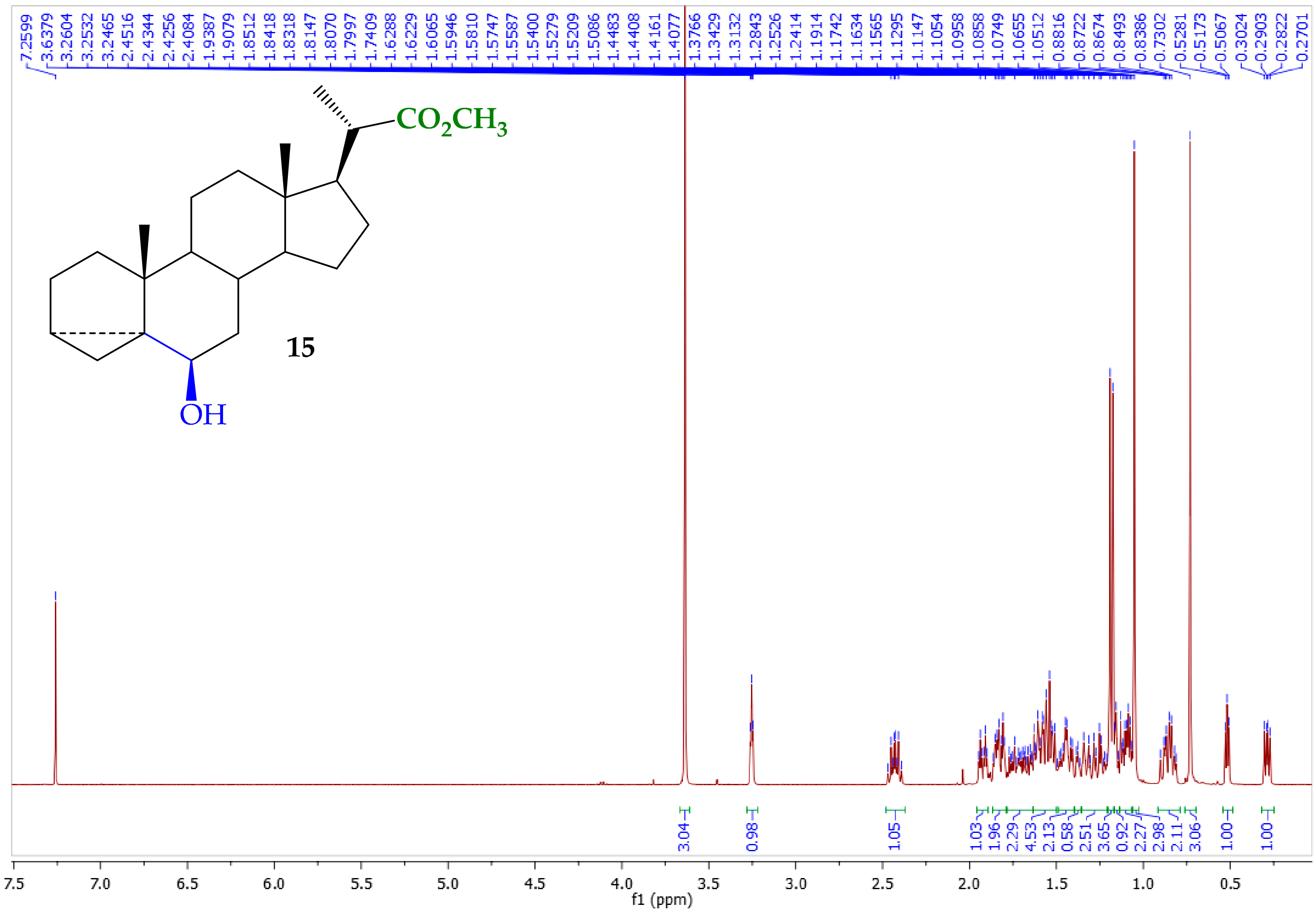

Figure S10. ^{1}H NMR spectrum of Methyl (20S)-6β-hydroxy-3α,5-cyclo-5α-pregnane-20-carboxylate (**15**)

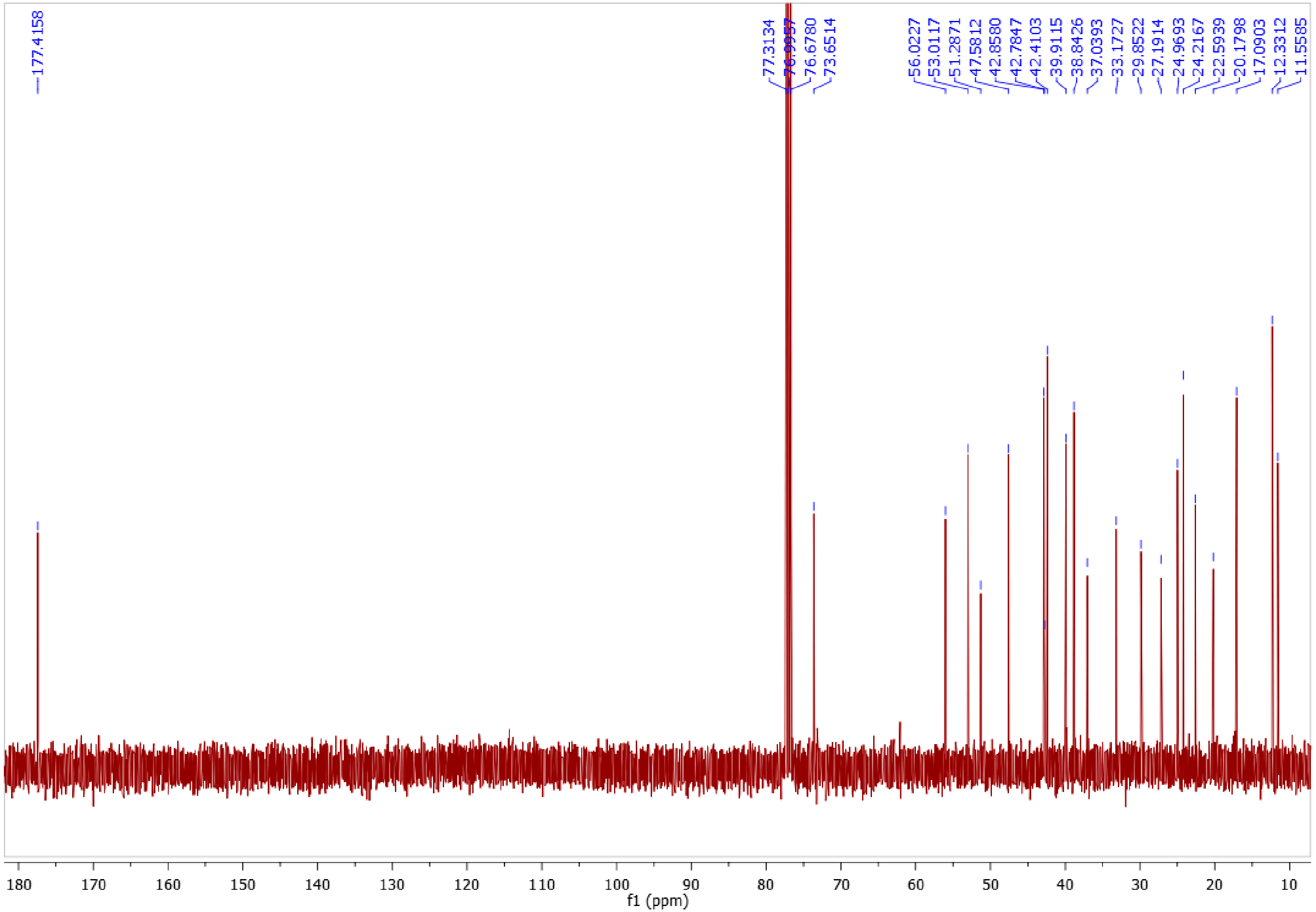

Figure S11. ^{13}C NMR spectrum of Methyl (20S)-6β-hydroxy-3*α*,5-cyclo-5*α*-pregnane-20-carboxylate (**15**)

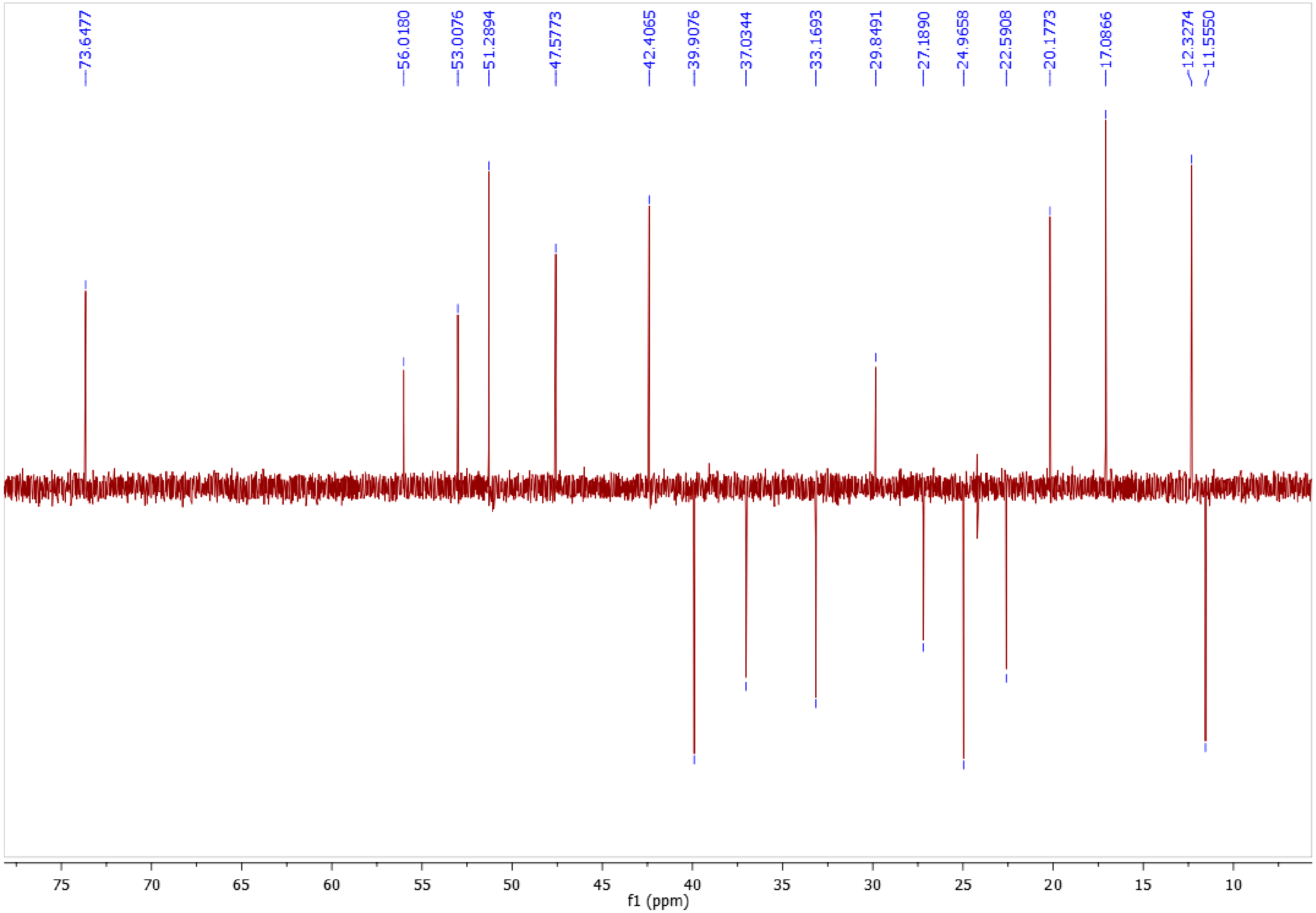

Figure S12. ^{13}C DEPT-135 NMR spectrum of Methyl (20S)-6β-hydroxy-3α,5-cyclo-5α-pregnane-20-carboxylate (**15**)

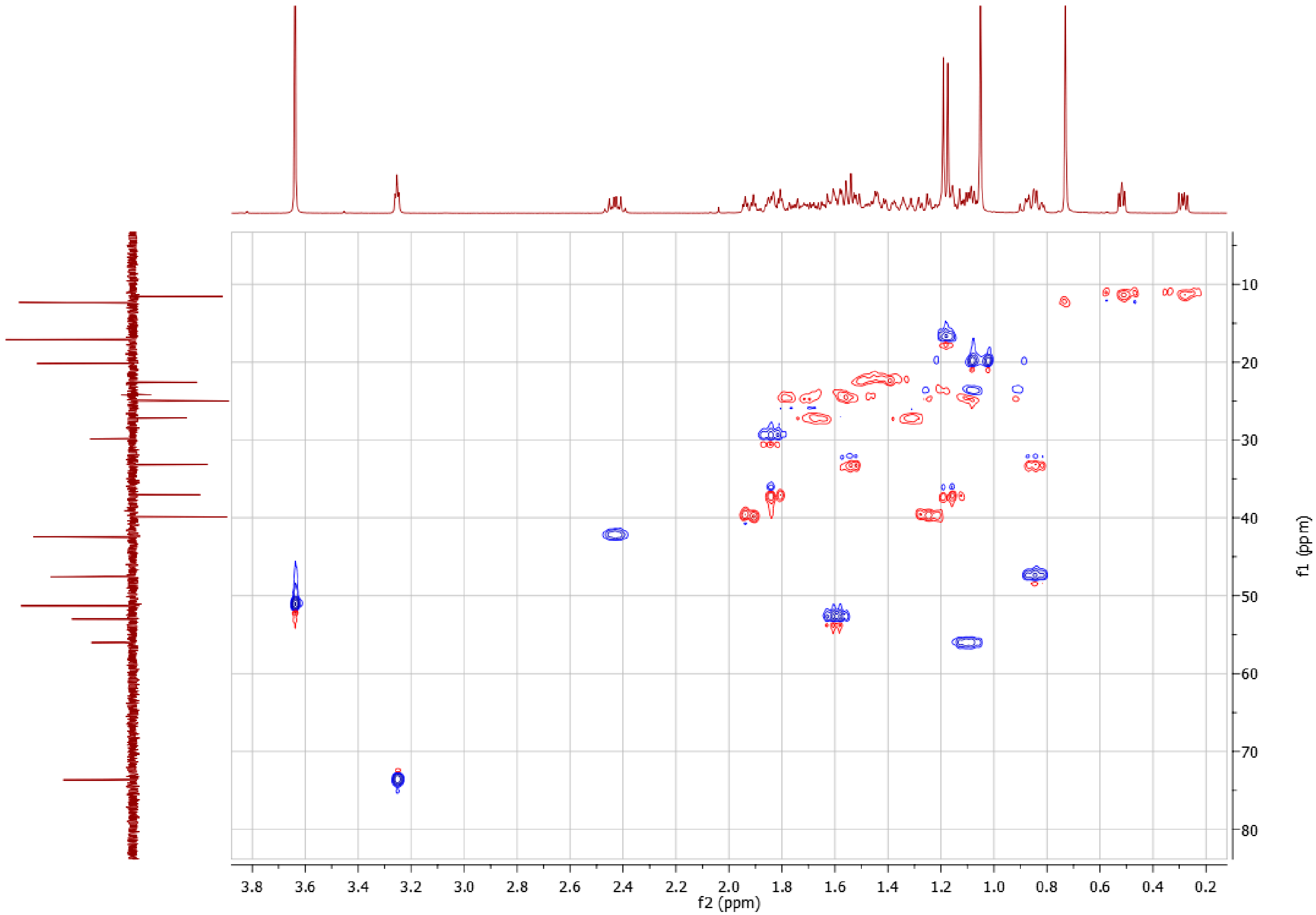

Figure S13. 2D HSQC NMR spectrum of Methyl (20S)-6β-hydroxy-3α,5-cyclo-5α-pregnane-20-carboxylate (**15**)

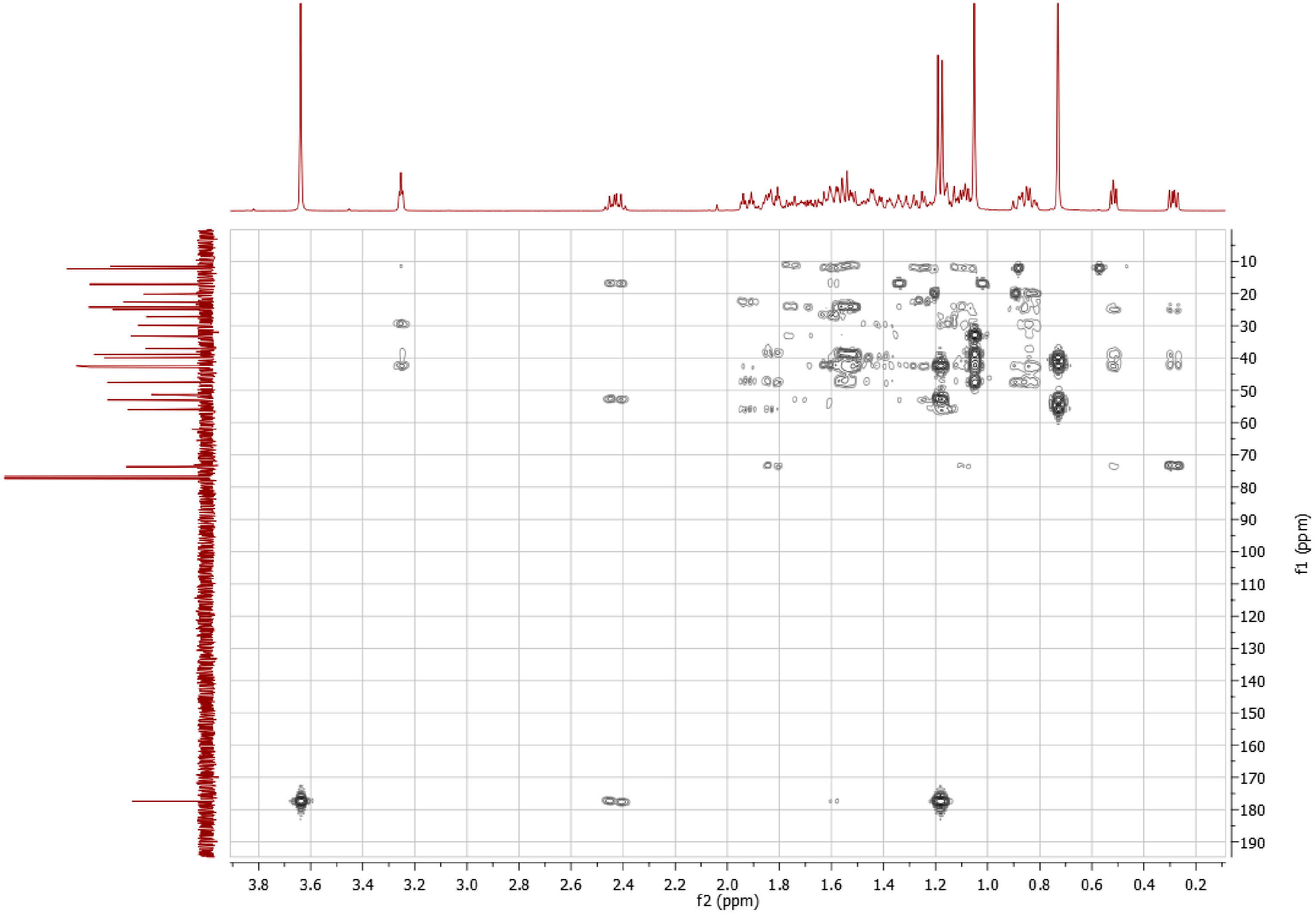

Figure S14. 2D HMBC NMR spectrum of Methyl (20S)-6β-hydroxy-3α,5-cyclo-5α-pregnane-20-carboxylate (**15**)

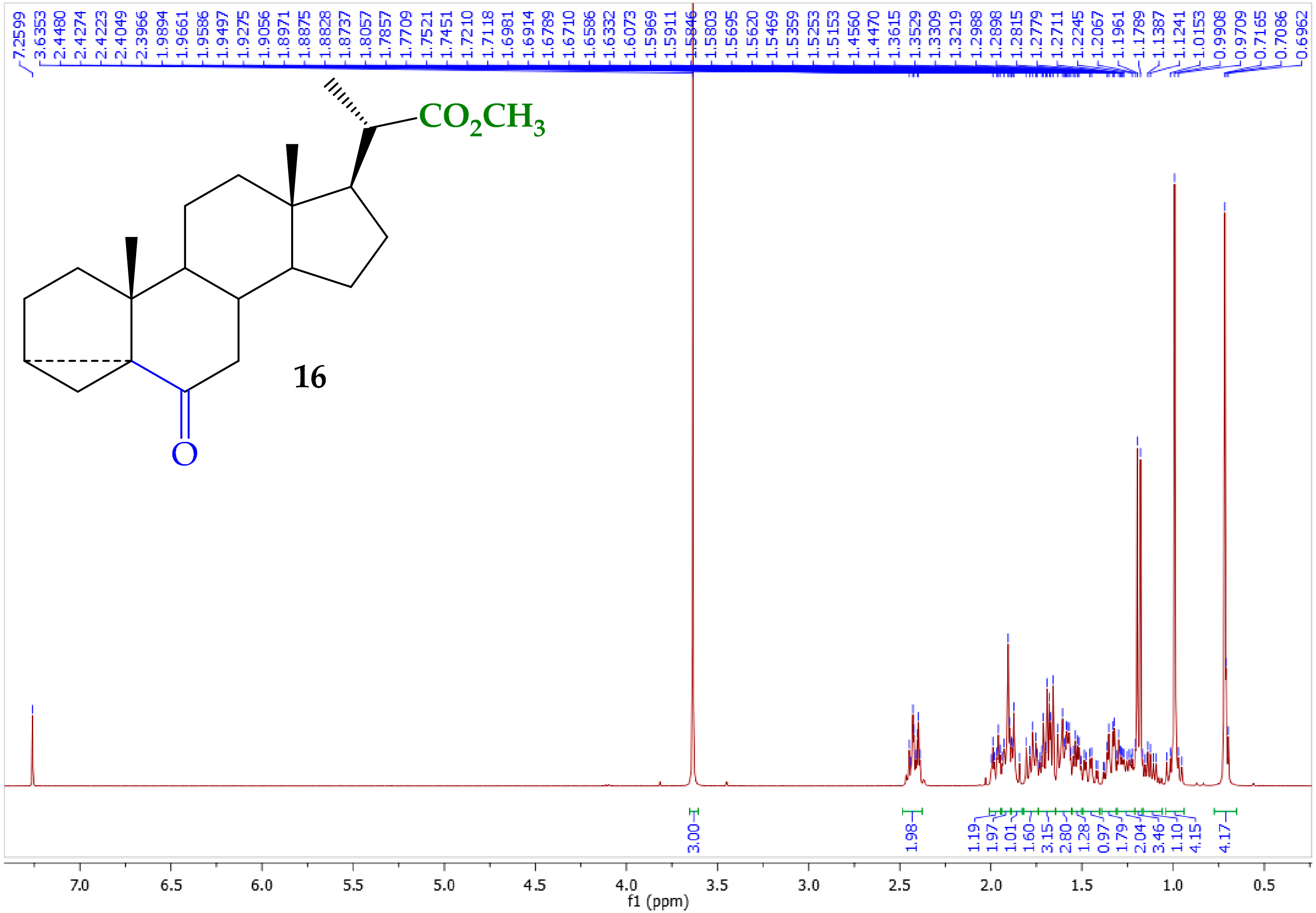

Figure S15. ^{1}H NMR spectrum of Methyl (20S)-6-Oxo-3α,5-cyclo-5α-pregnane-20-carboxylate (**16**)

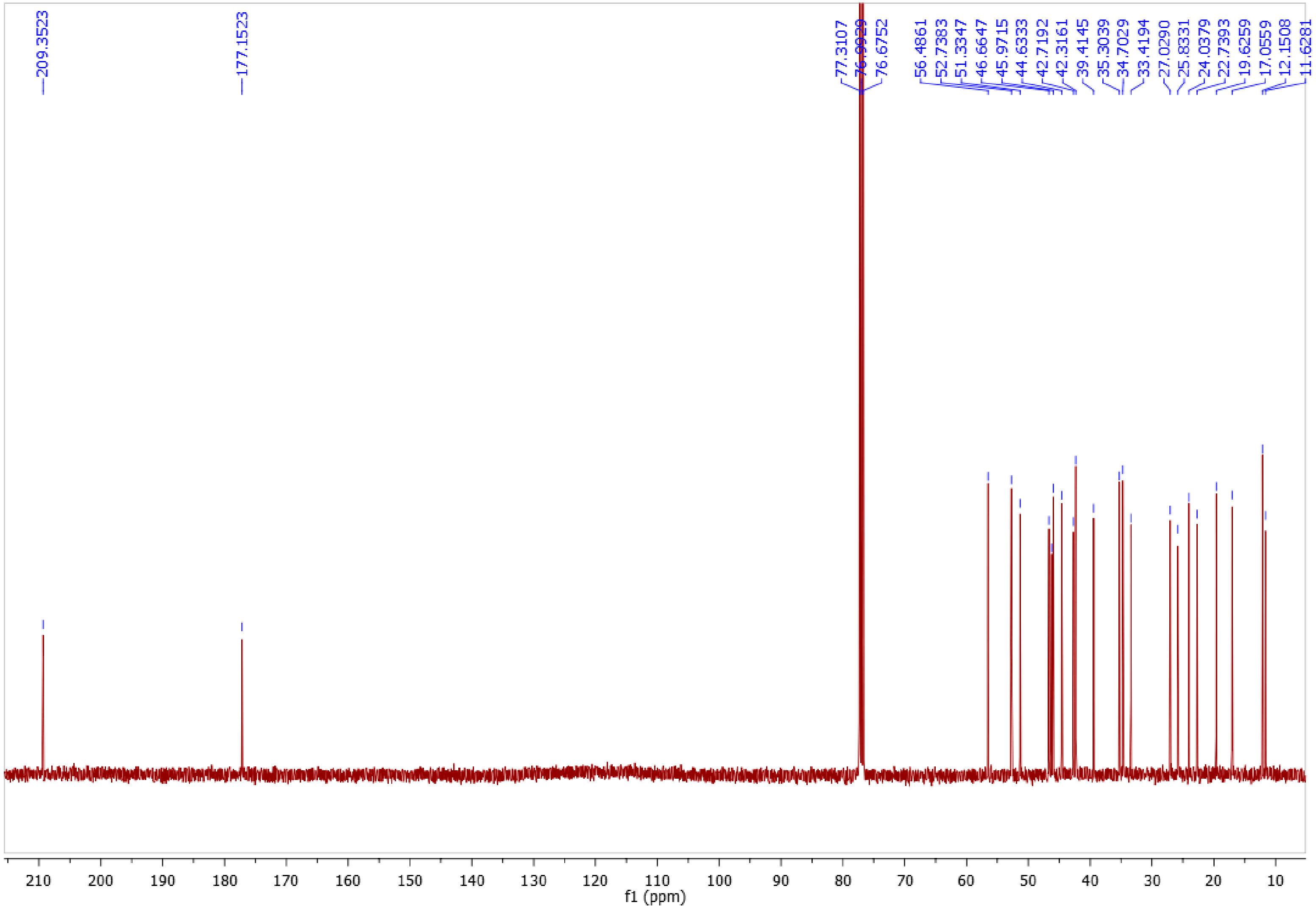

Figure S16. ^{13}C NMR spectrum of Methyl (20S)-6-Oxo-3α,5-cyclo-5α-pregnane-20-carboxylate (**16**)

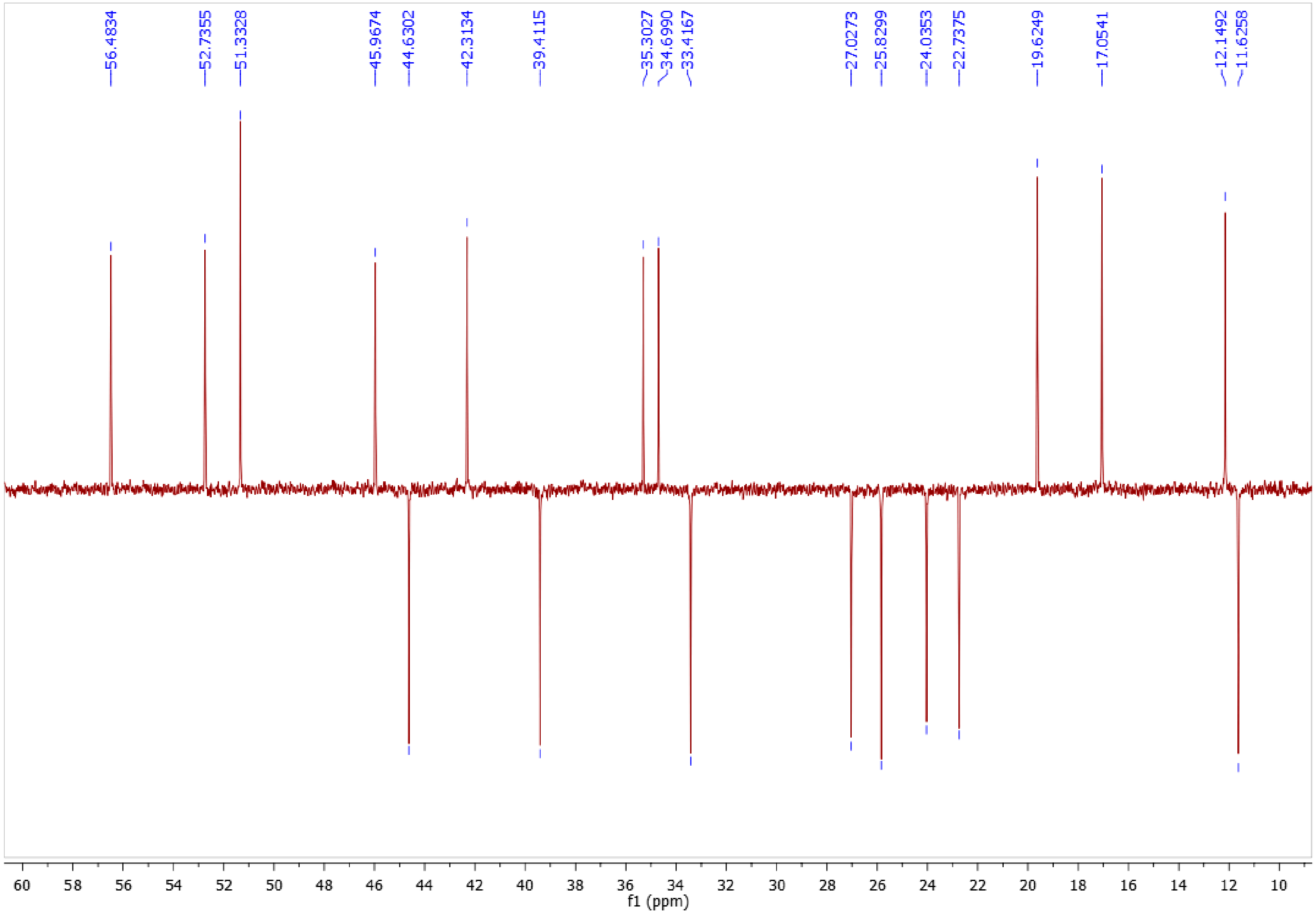

Figure S17. ^{13}C DEPT-135 NMR spectrum of Methyl (20S)-6-Oxo-3α,5-cyclo-5α-pregnane-20-carboxylate (**16**)

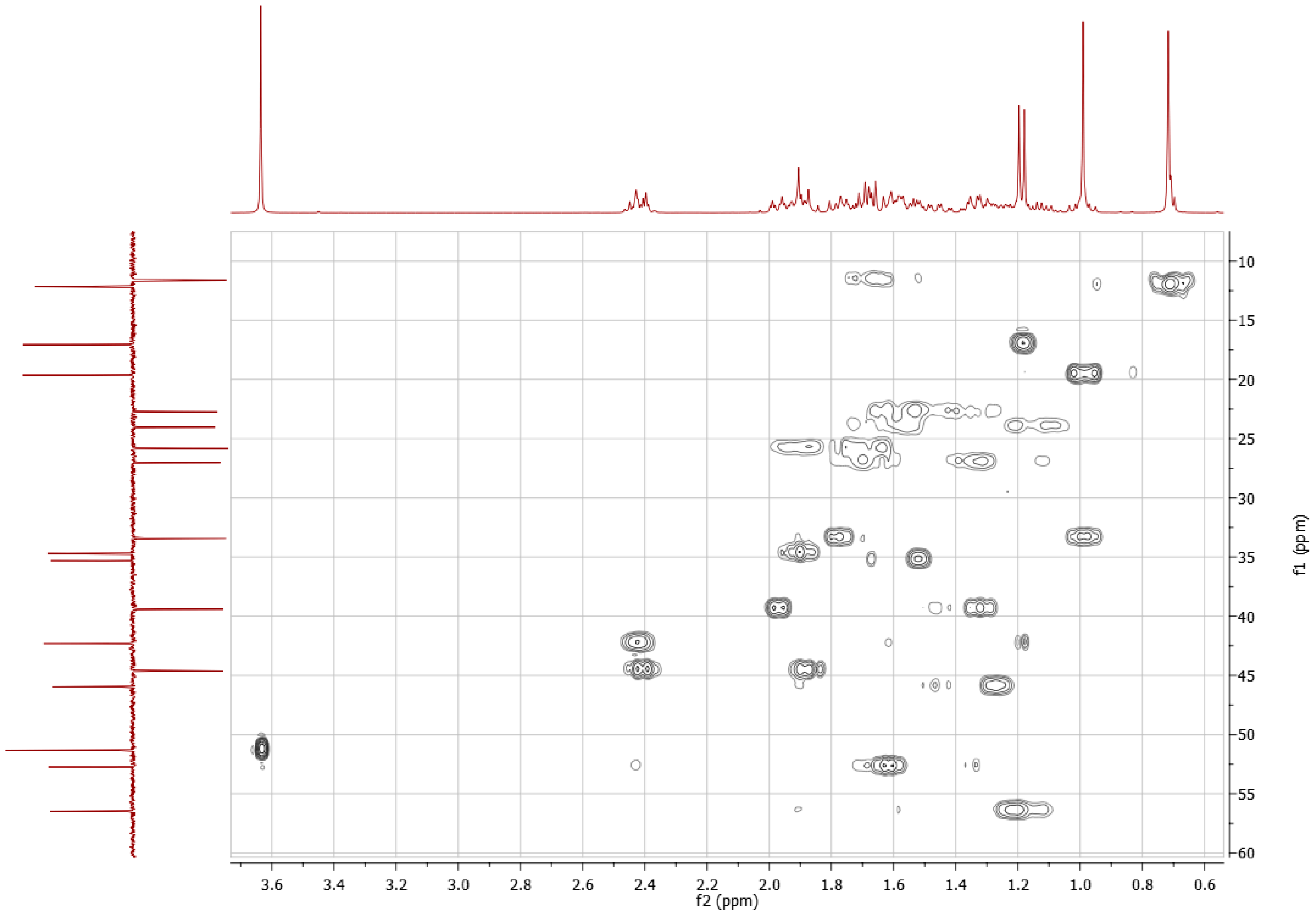

Figure S18. 2D HSQC NMR spectrum of Methyl (20S)-6-Oxo-3α,5-cyclo-5α-pregnane-20-carboxylate (**16**)

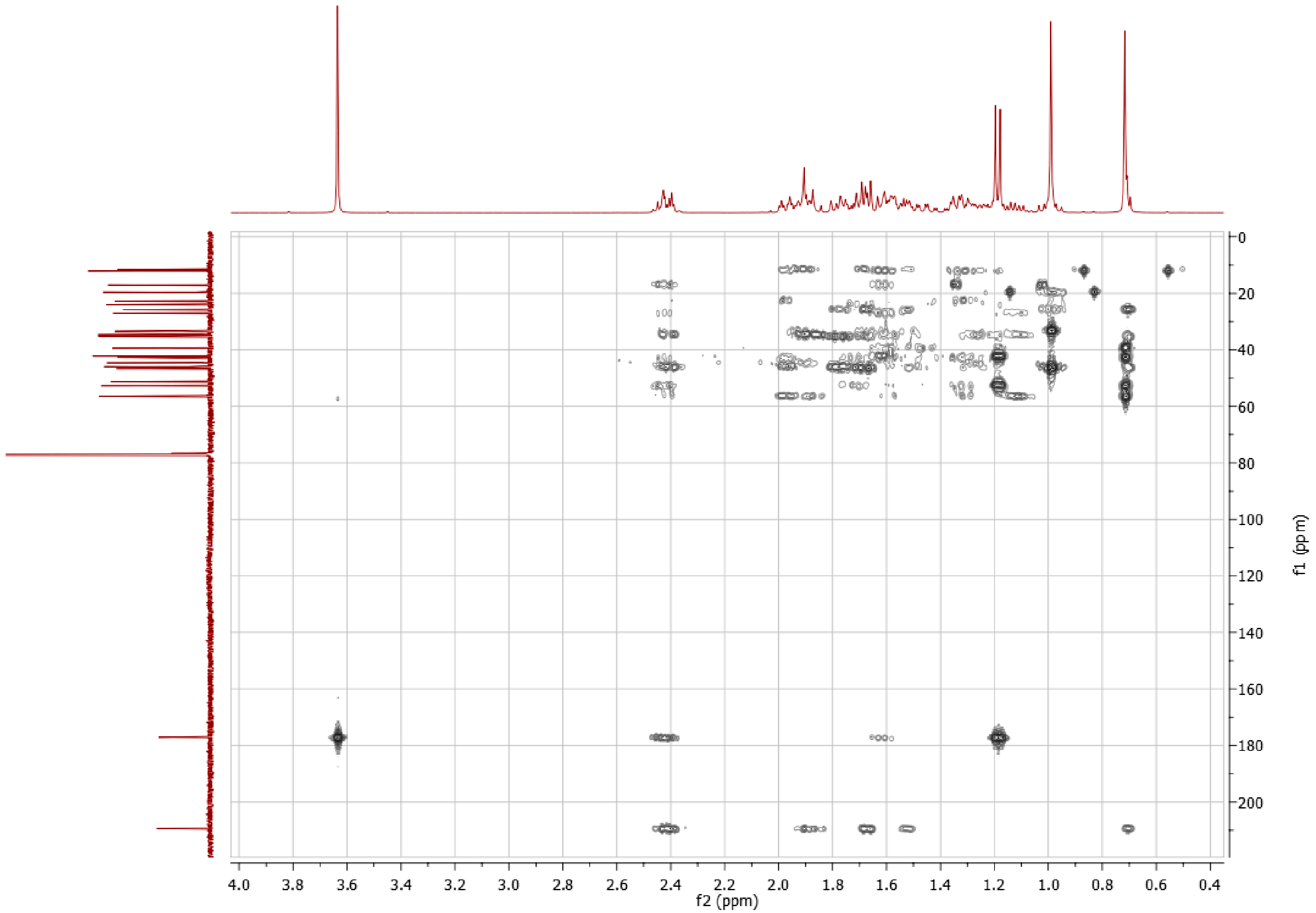

Figure S19. 2D HMBC NMR spectrum of Methyl (20S)-6-Oxo-3α,5-cyclo-5α-pregnane-20-carboxylate (**16**)

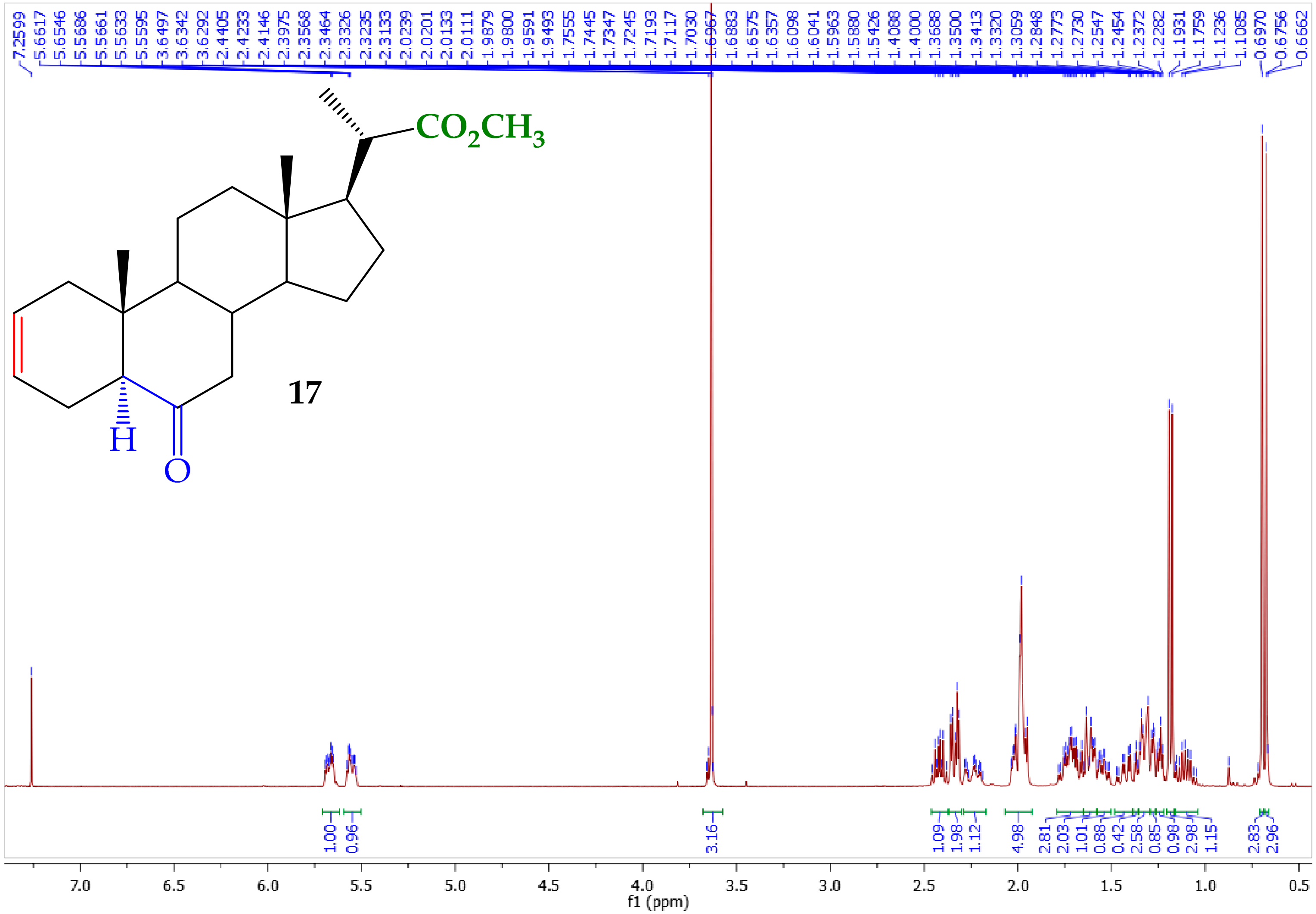

Figure S20. ^{1}H NMR spectrum of Methyl (20S)-6-Oxo-5α-pregn-2-ene-20-carboxylate (**17**)

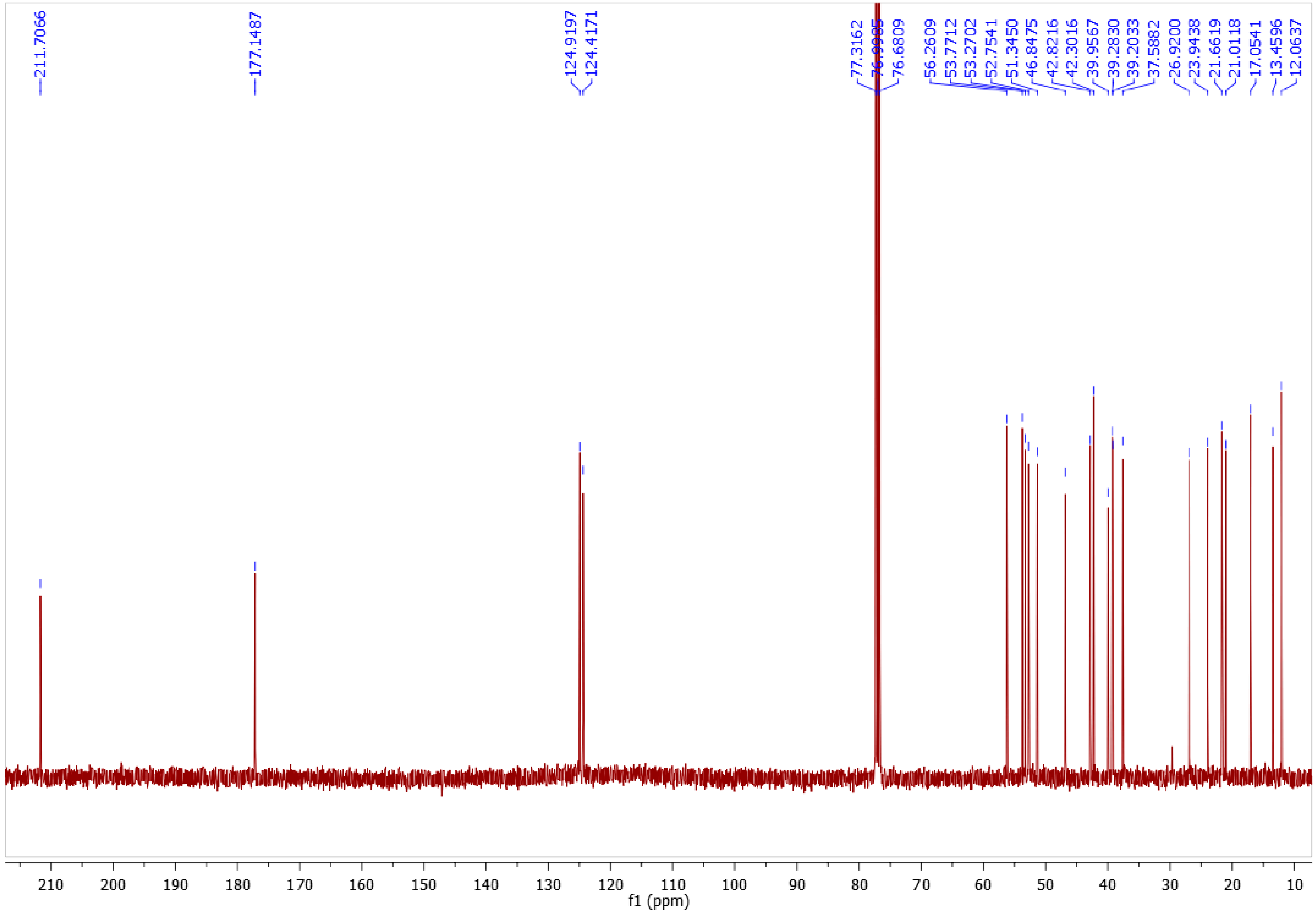

Figure S21. ^{13}C NMR spectrum of Methyl (20S)-6-Oxo-5α-pregn-2-ene-20-carboxylate (**17**)

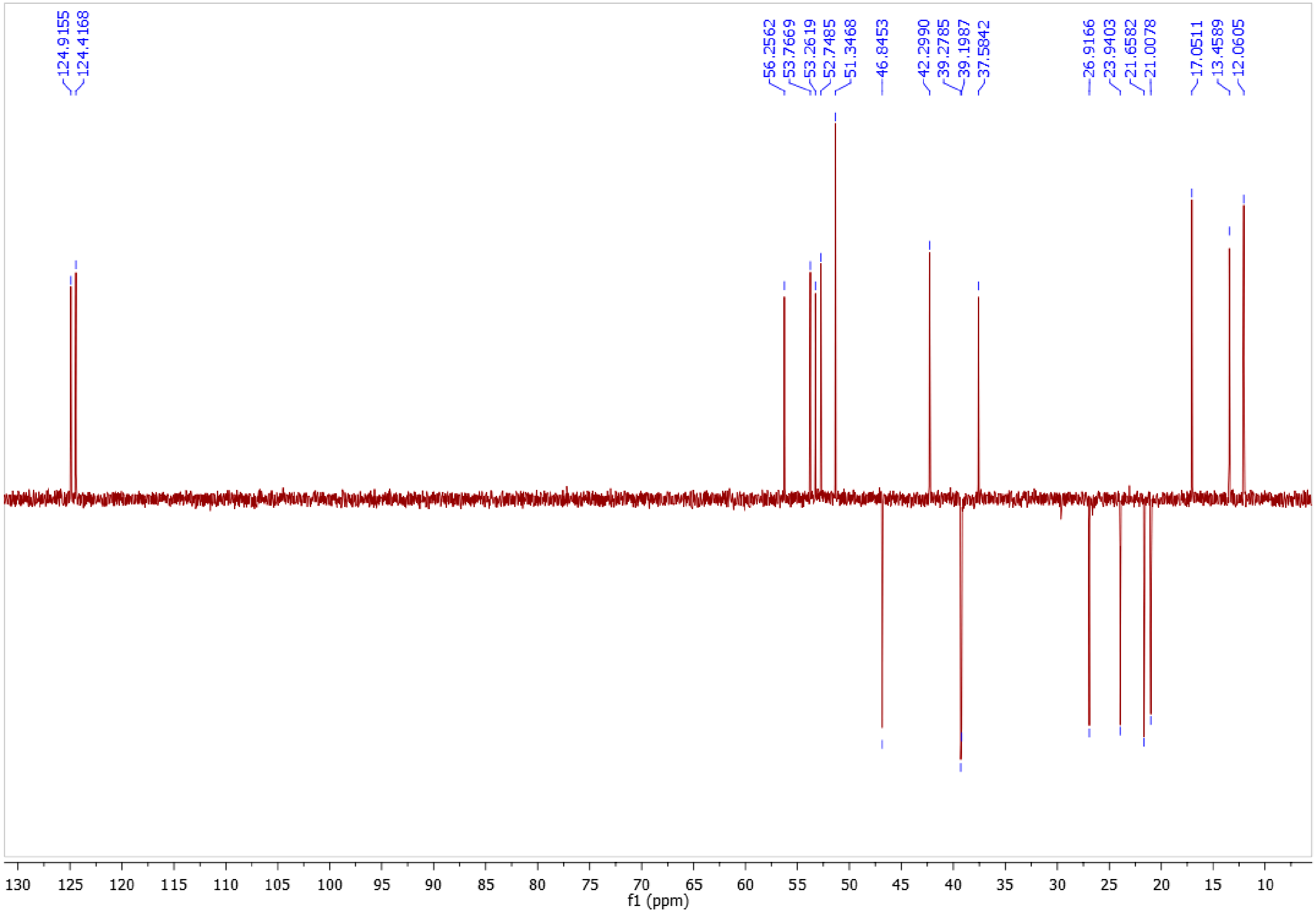

Figure S22. ^{13}C DEPT-135 NMR spectrum of Methyl (20S)-6-Oxo-5α-pregn-2-ene-20-carboxylate (**17**)

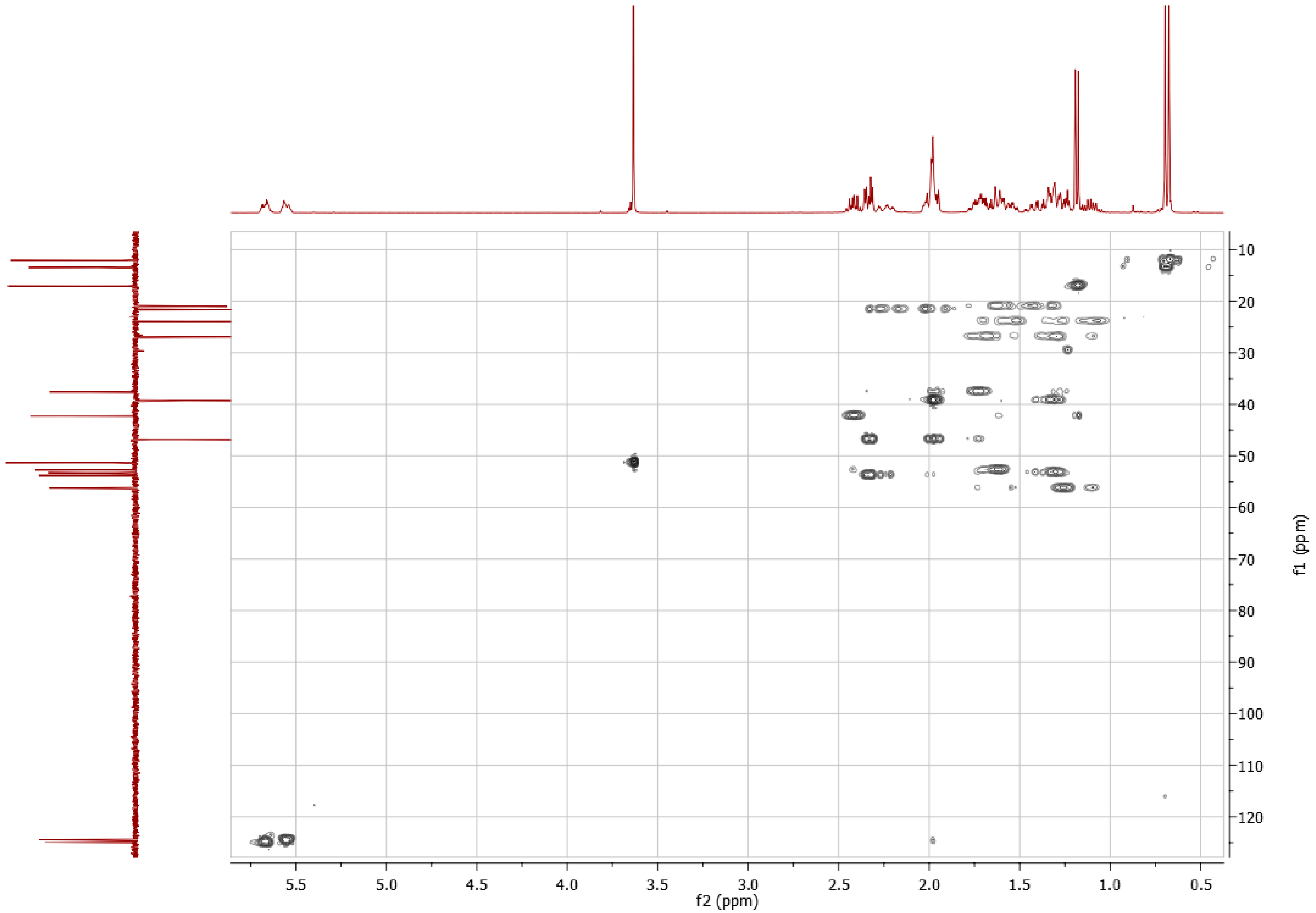

Figure S23. 2D HSQC NMR spectrum of Methyl (20S)-6-Oxo-5α-pregn-2-ene-20-carboxylate (**17**)

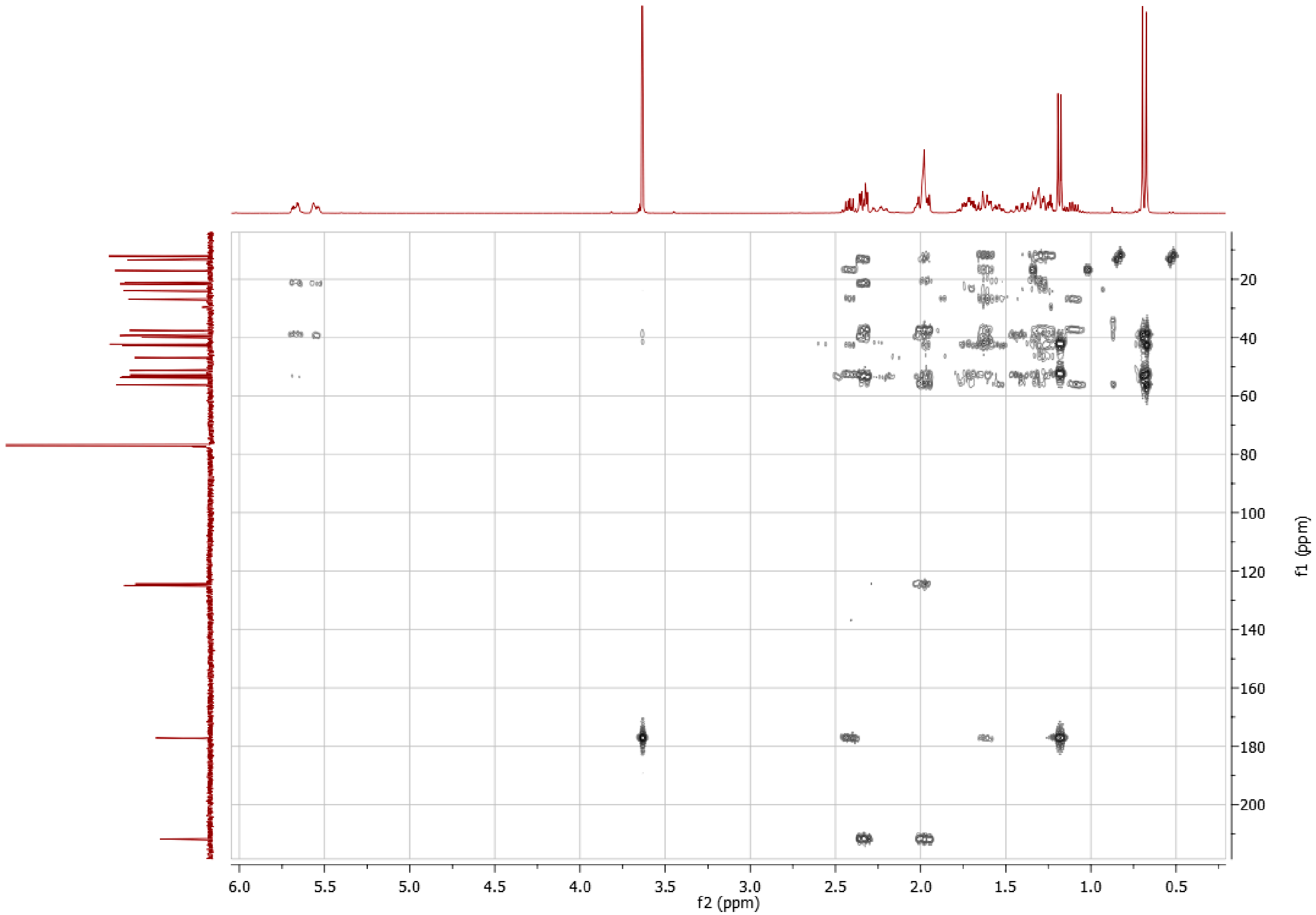

Figure S24. 2D HSQC NMR spectrum of Methyl (20S)-6-Oxo-5α-pregn-2-ene-20-carboxylate (**17**)

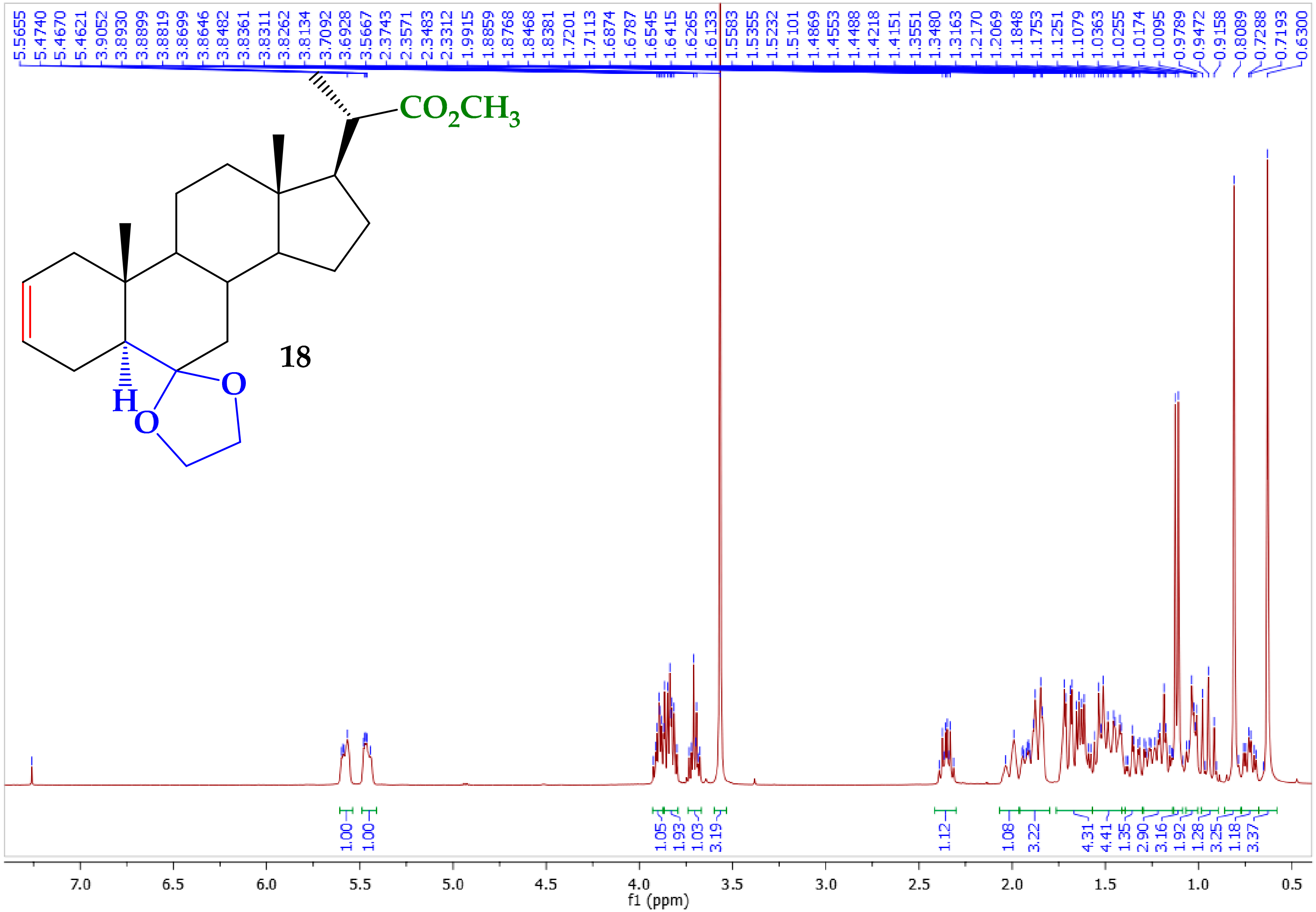

Figure S25. ^{1}H NMR spectrum of Methyl (20S)-6,6-ethylenedioxy-5α-pregn-2-ene-20-carboxylate (**18**)

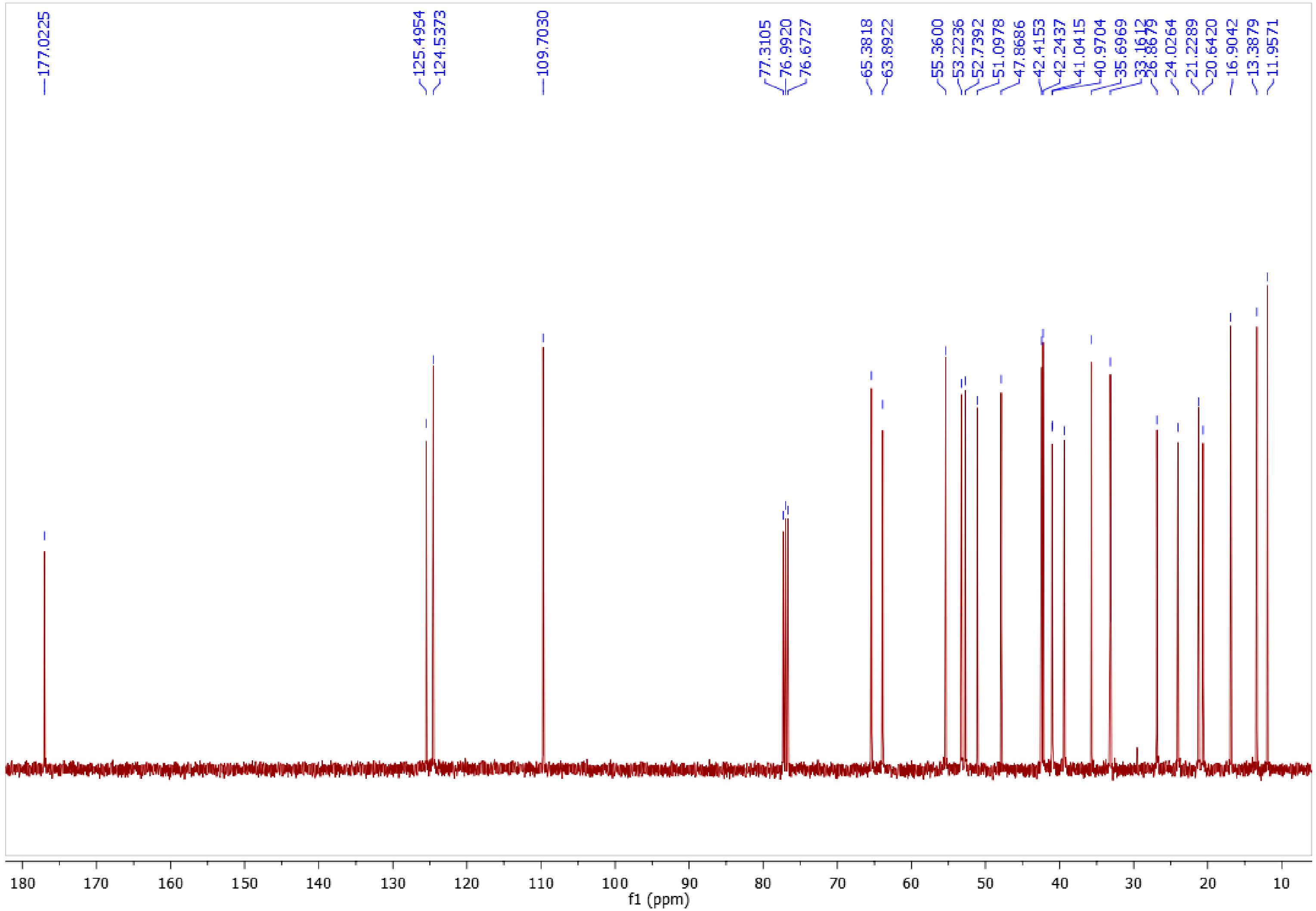

Figure S26. ^{13}C NMR spectrum of Methyl (20S)-6,6-ethylenedioxy-5α-pregn-2-ene-20-carboxylate (**18**)

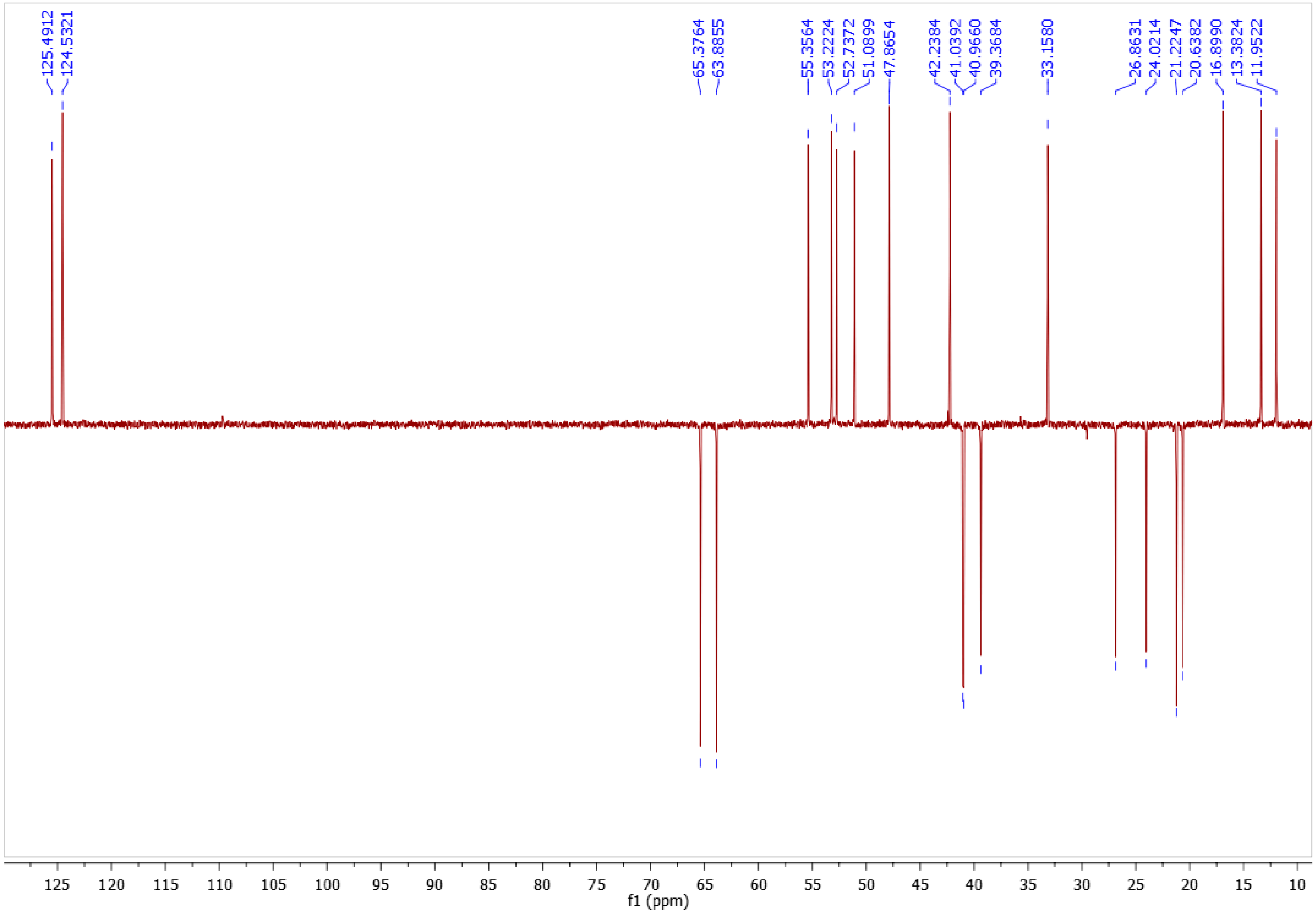

Figure S27. ^{13}C DEPT-135 NMR spectrum of Methyl (20S)-6,6-ethylenedioxy-5α-pregn-2-ene-20-carboxylate (**18**)

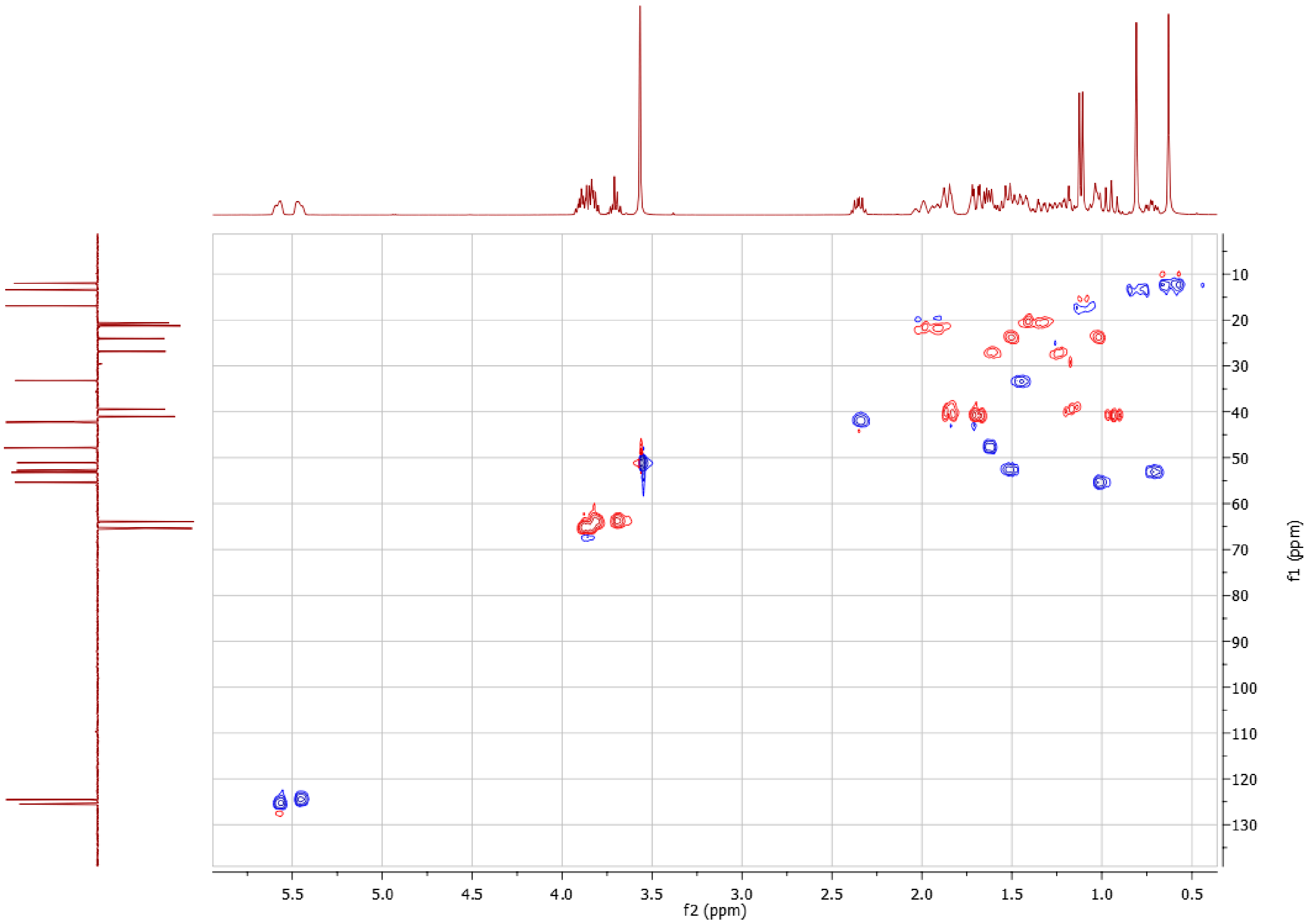

Figure S28. 2D HSQC NMR spectrum of Methyl (20S)-6,6-ethylenedioxy-5α-pregn-2-ene-20-carboxylate (**18**)

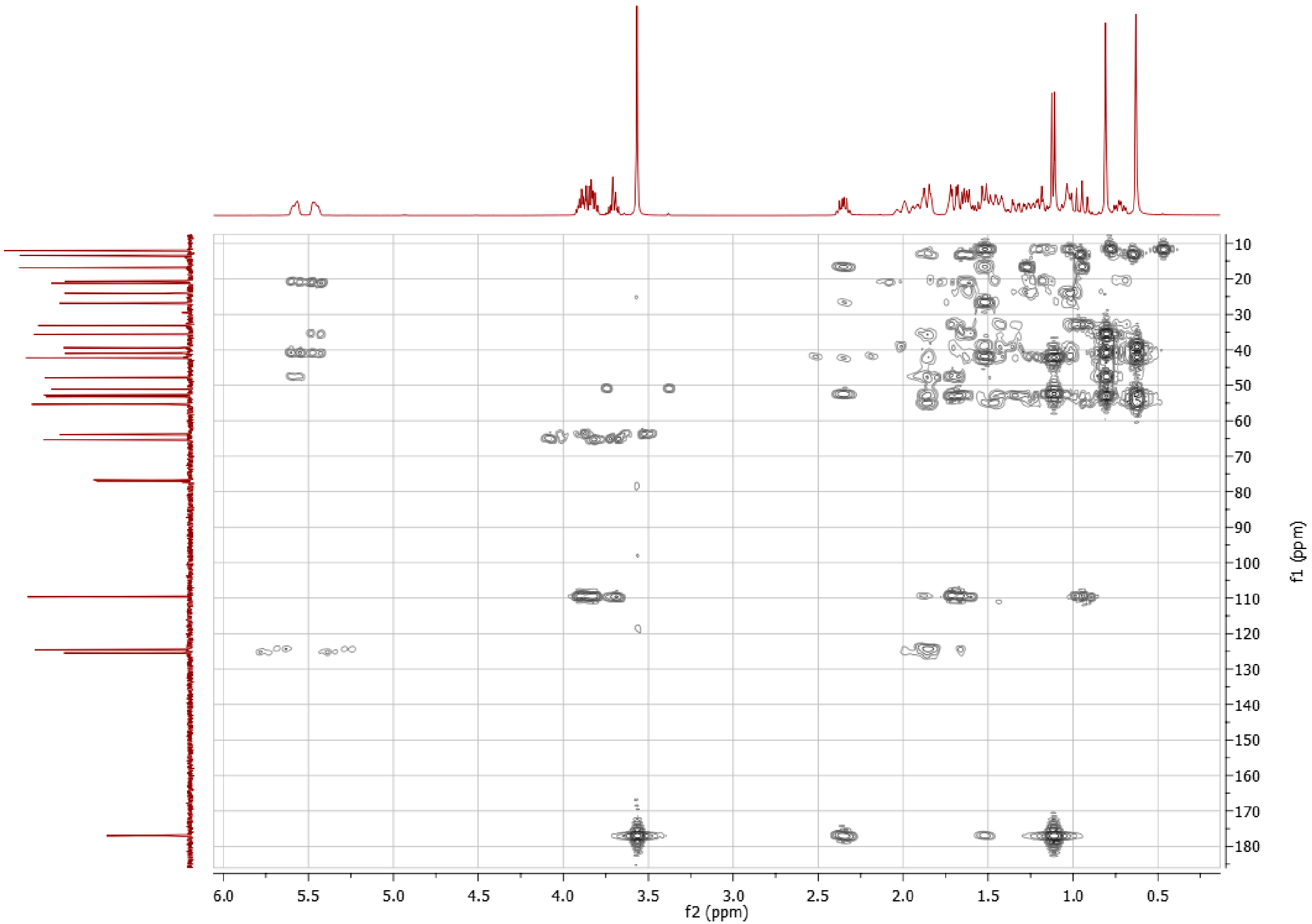

Figure S29. 2D HMBC NMR spectrum of Methyl (20S)-6,6-ethylenedioxy-5α-pregn-2-ene-20-carboxylate (**18**)

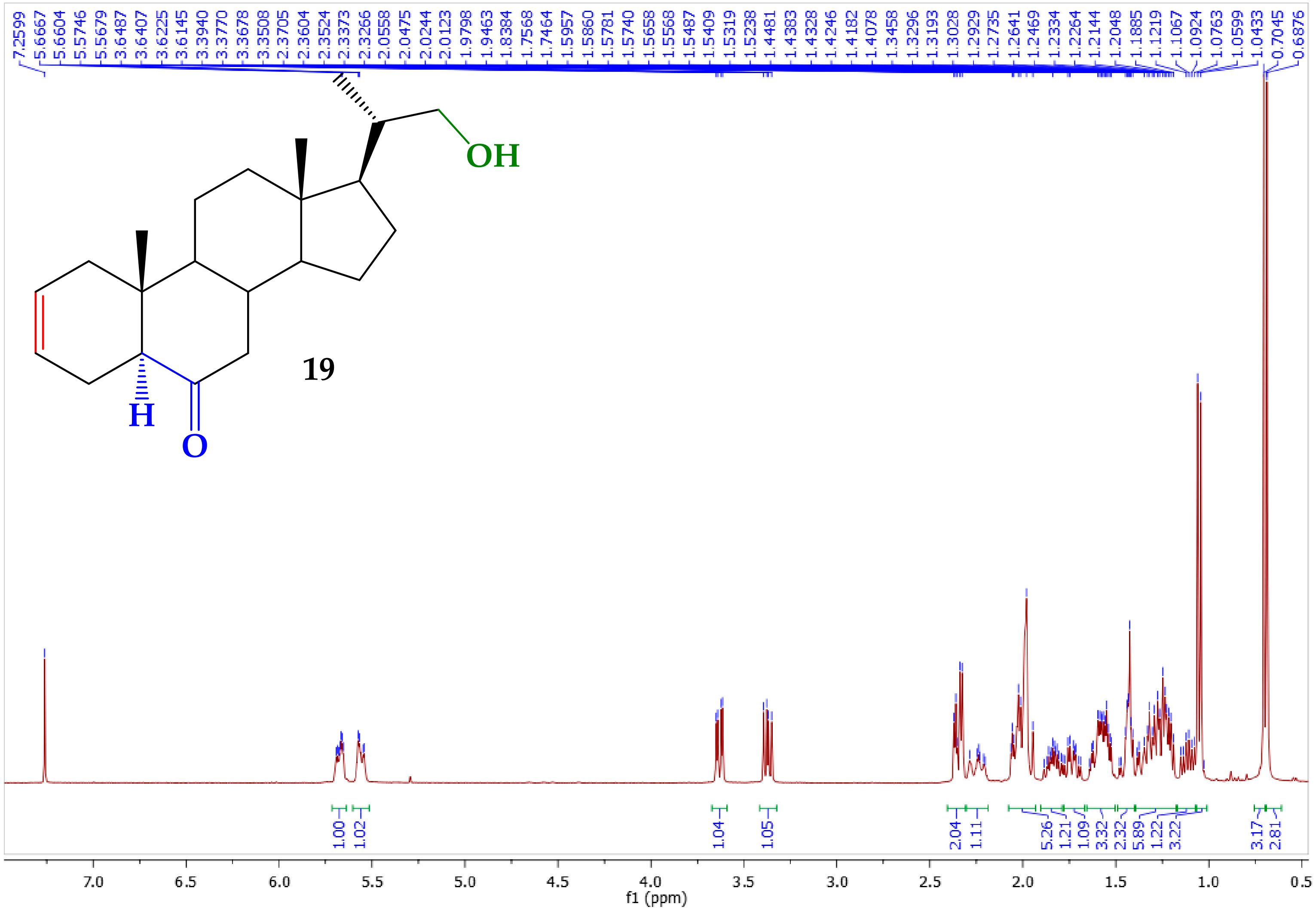

Figure S30. ^{1}H NMR spectrum of 22-hydroxy-5α-cholan-2-ene-23,24-dinor-6-one (**19**)

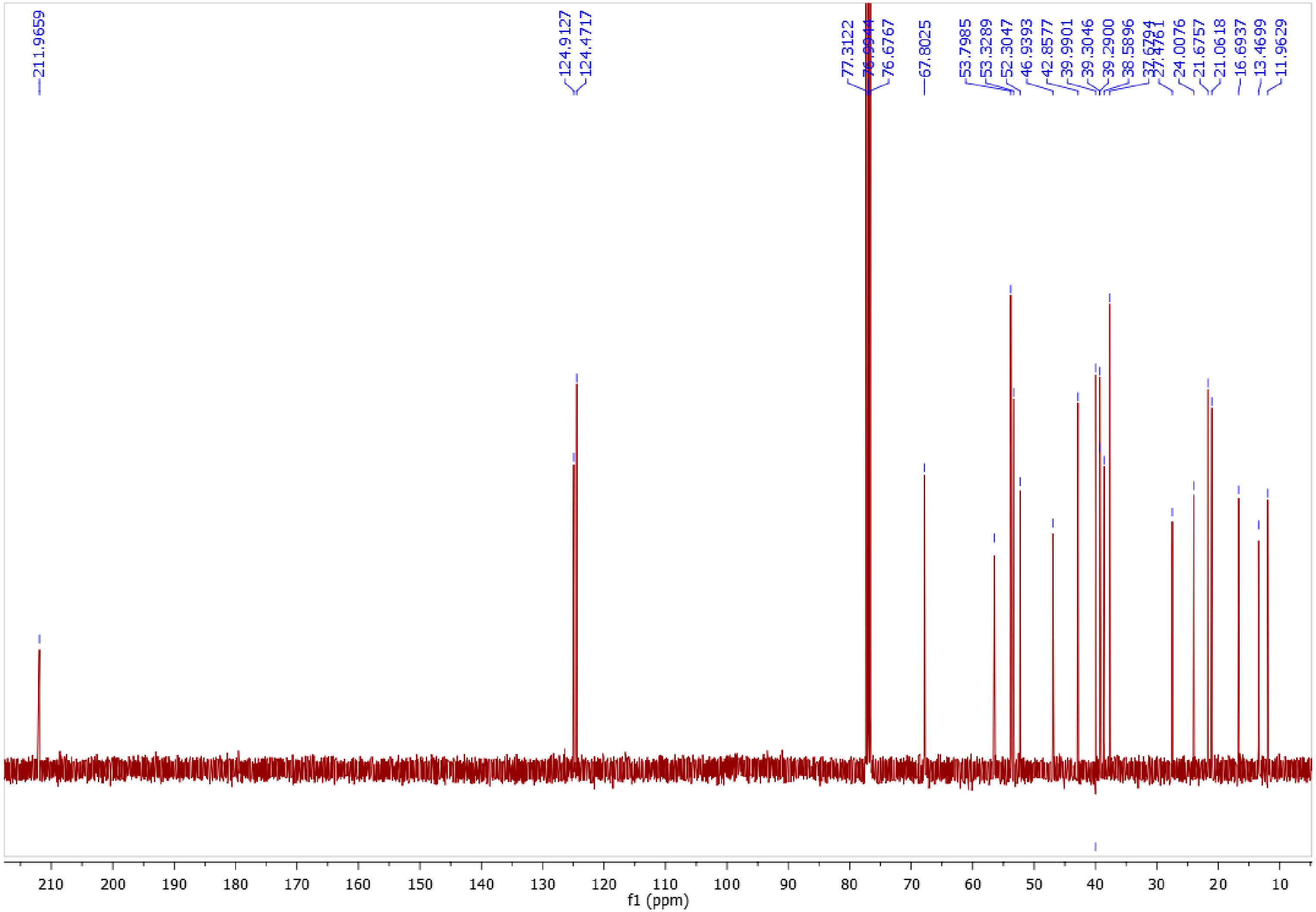

Figure S31. ^{13}C NMR spectrum of 22-hydroxy-5α-cholan-2-ene-23,24-dinor-6-one (**19**)

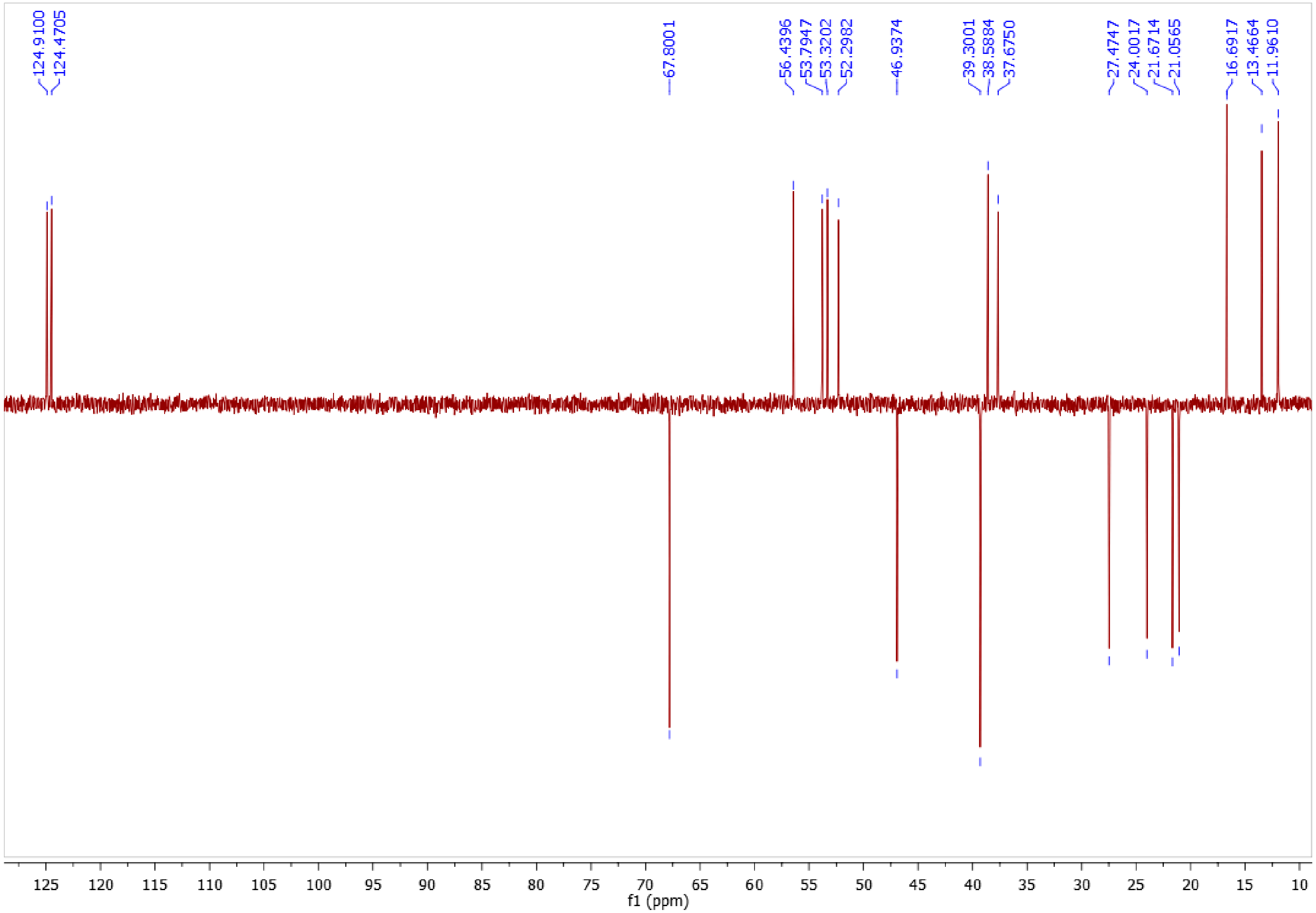

Figure S32. ^{13}C DEPT-135 NMR spectrum of 22-hydroxy-5α-cholan-2-ene-23,24-dinor-6-one (**19**)

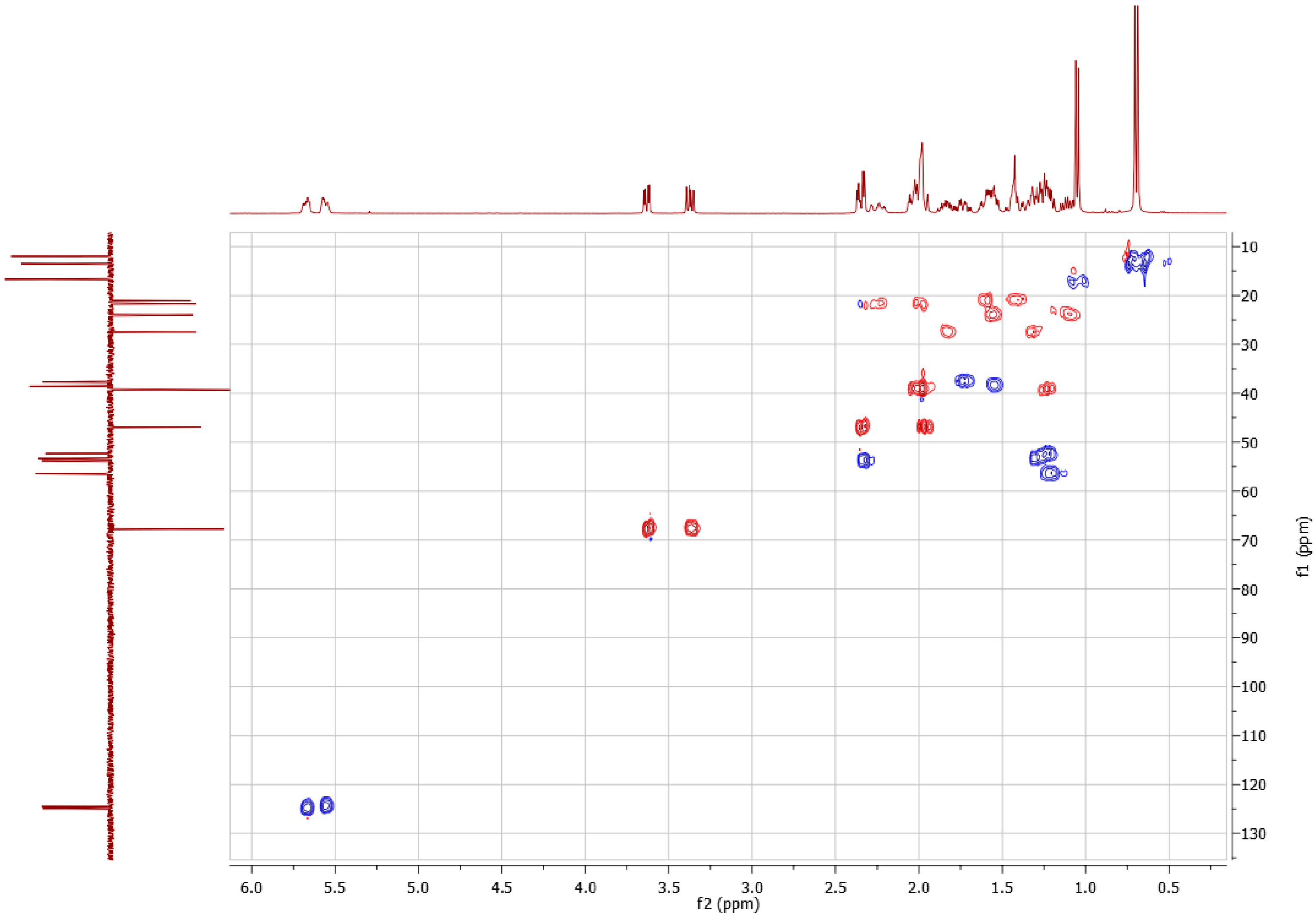

Figure S33. 2D HSQC NMR spectrum of 22-hydroxy-5α-cholan-2-ene-23,24-dinor-6-one (**19**)

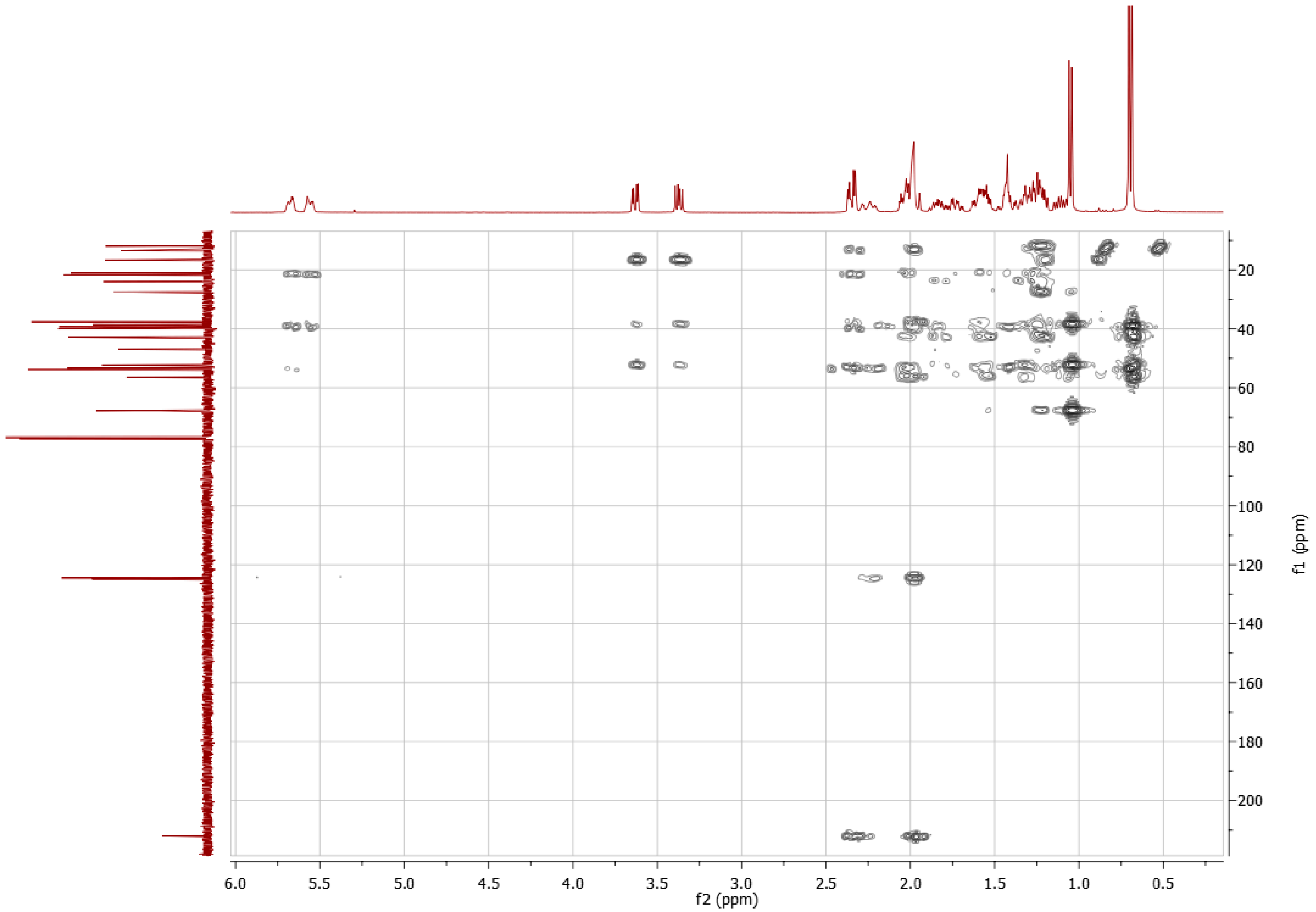

Figure S34. 2D HMBC NMR spectrum of 22-hydroxy-5α-cholan-2-ene-23,24-dinor-6-one (**19**)

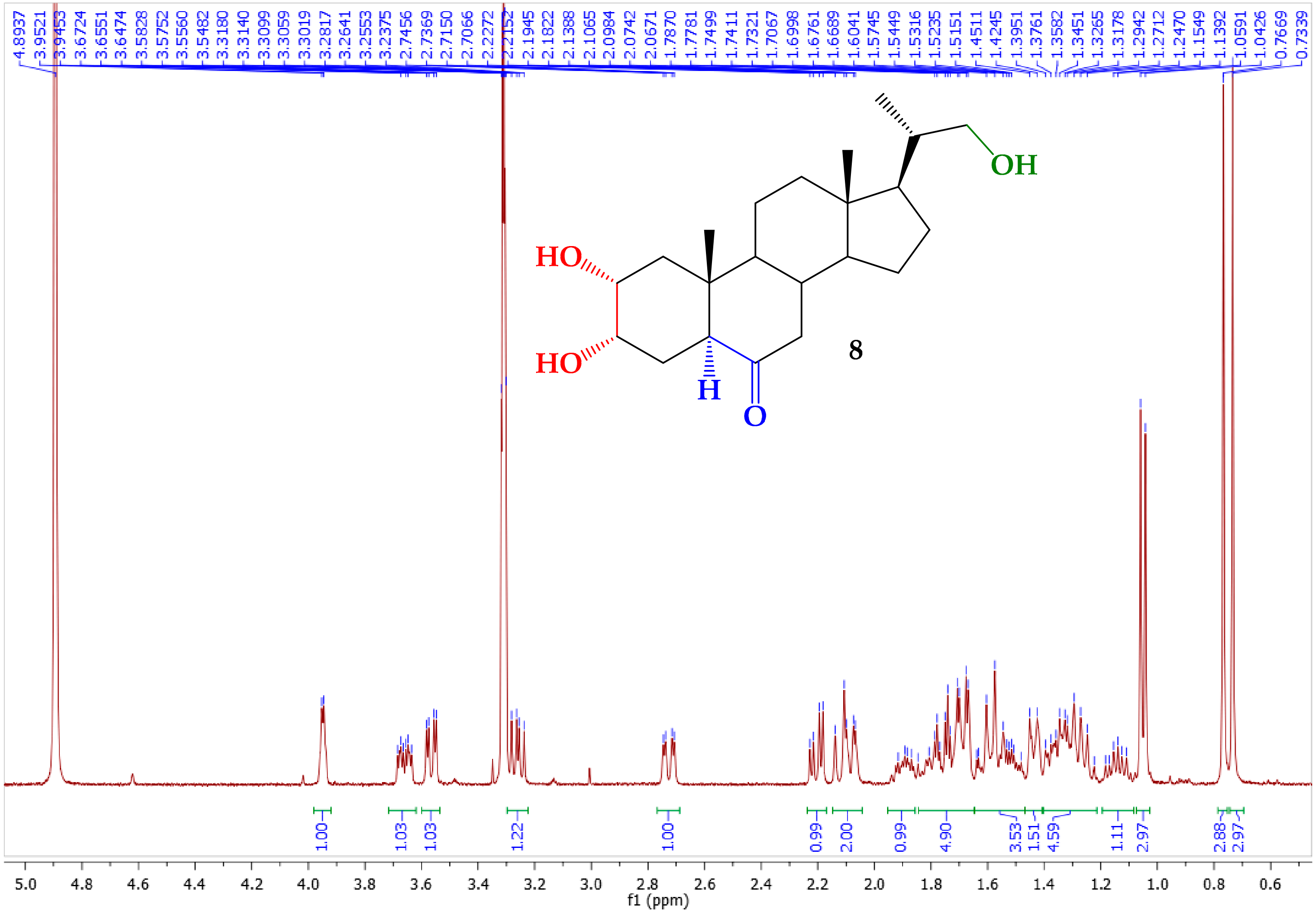

Figure S35. ^{1}H NMR spectrum of 2α,3α,22-trihydroxy-5α-cholan-23,24-dinor-6-one (**8**)

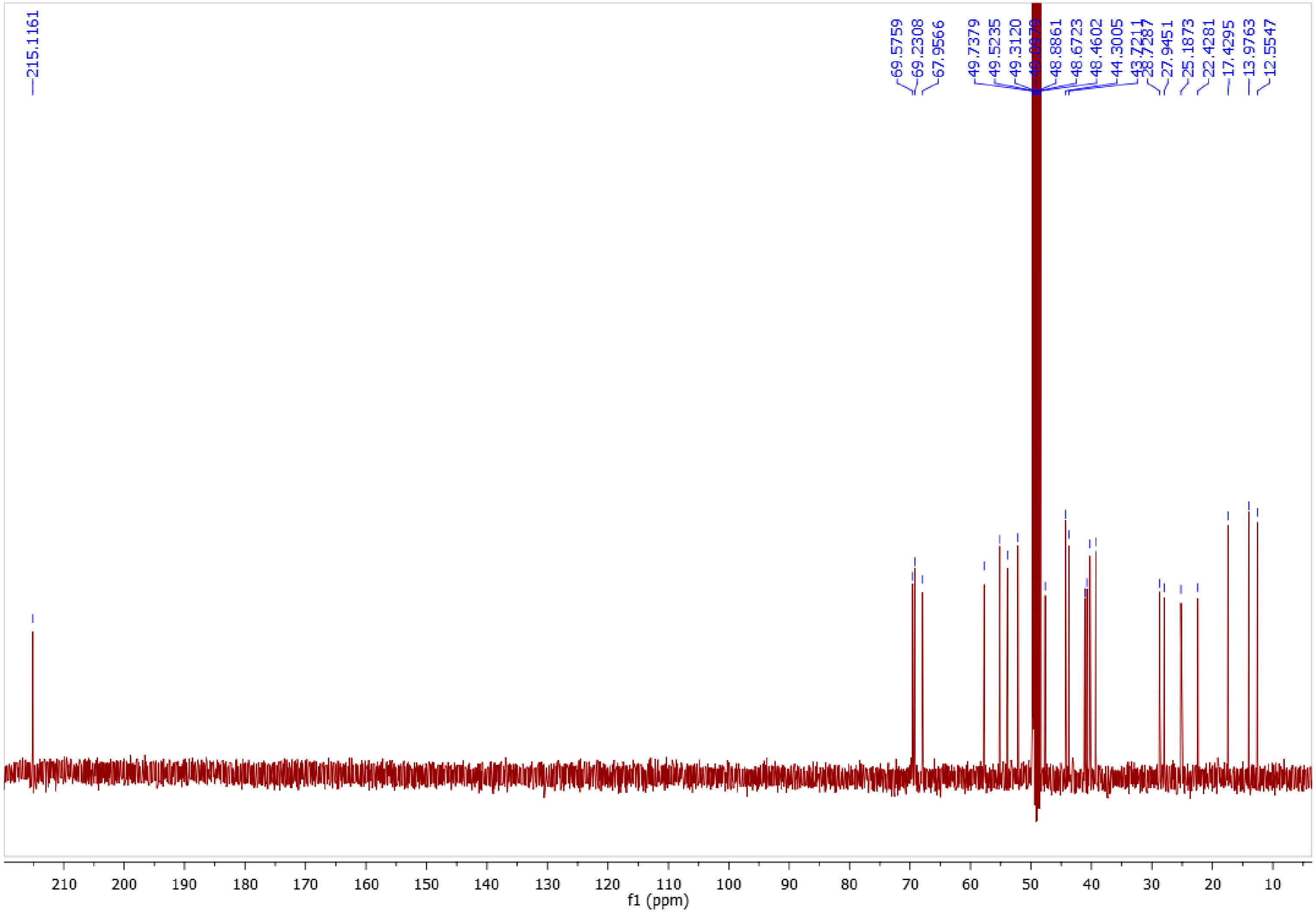

Figure S36. ^{13}C NMR spectrum of 2α,3α,22-thrihydroxy-5α-cholan-23,24-dinor-6-one (**8**)

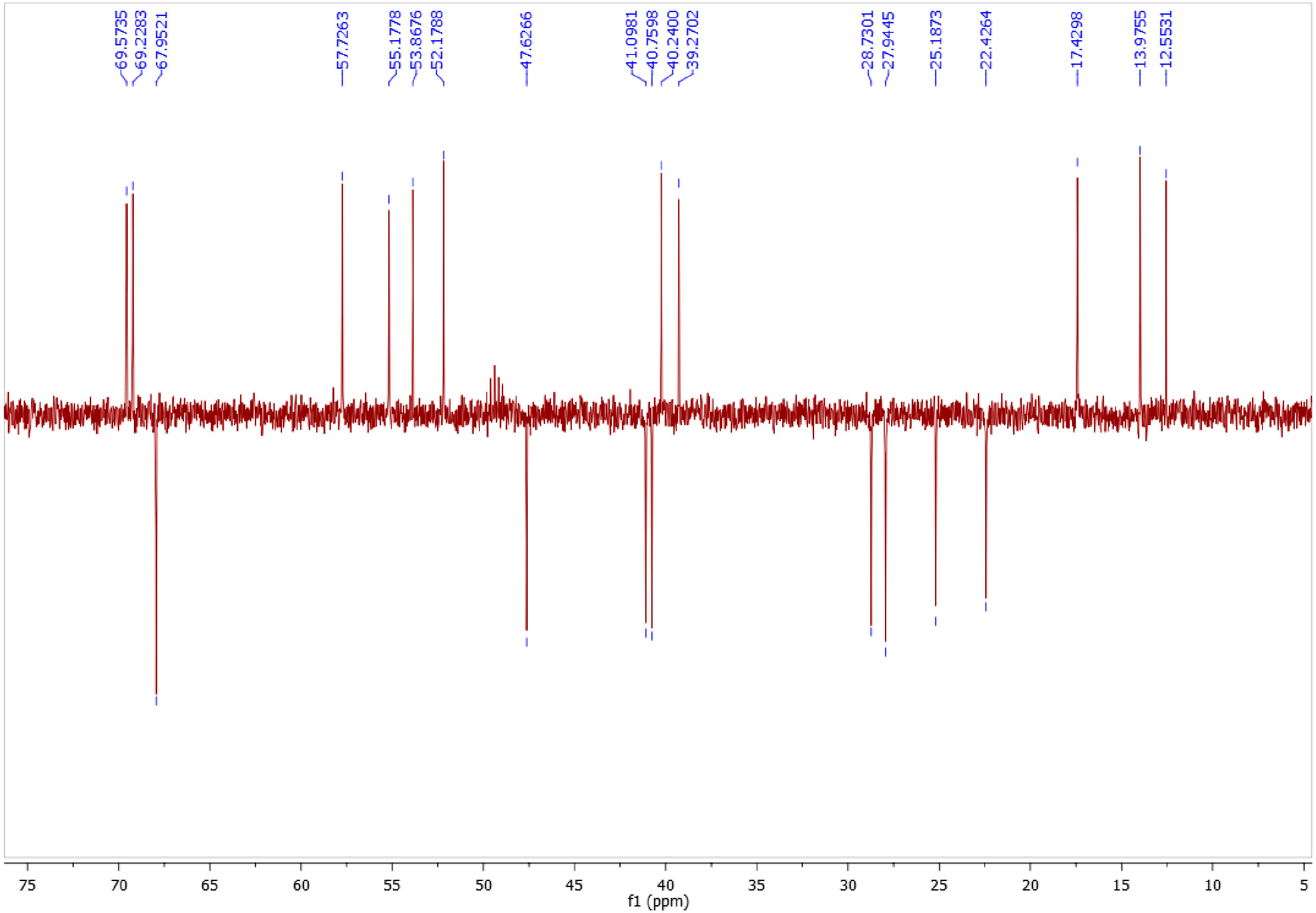

Figure S37. ^{13}C DEPT-135 NMR spectrum of 2*α*,3*α*,22-thrihydroxy-5*α*-cholan-23,24-dinor-6-one (**8**)

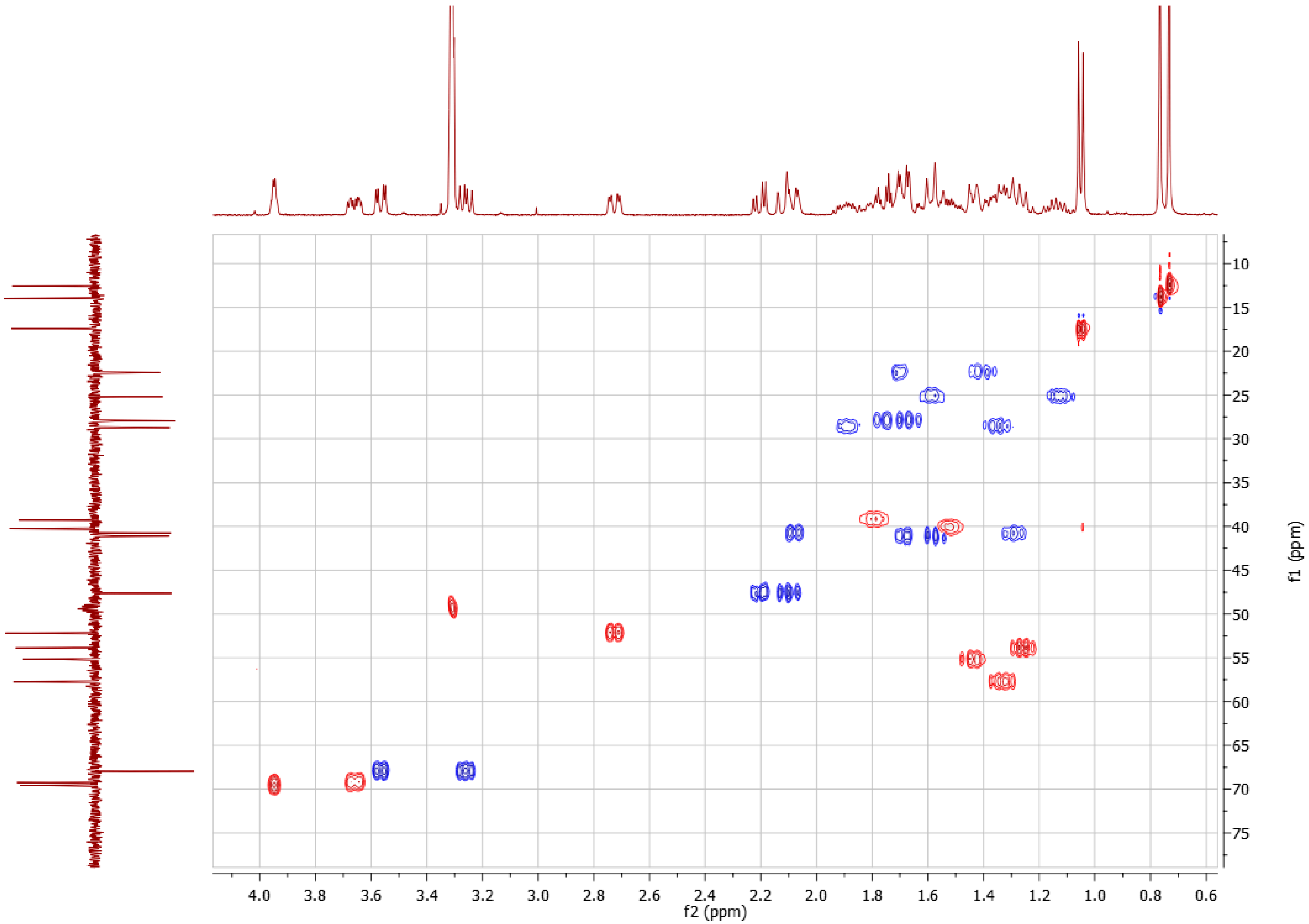

Figure S38. 2D HSQC NMR spectrum of 2α,3α,22-trihydroxy-5α-cholan-23,24-dinor-6-one (**8**)

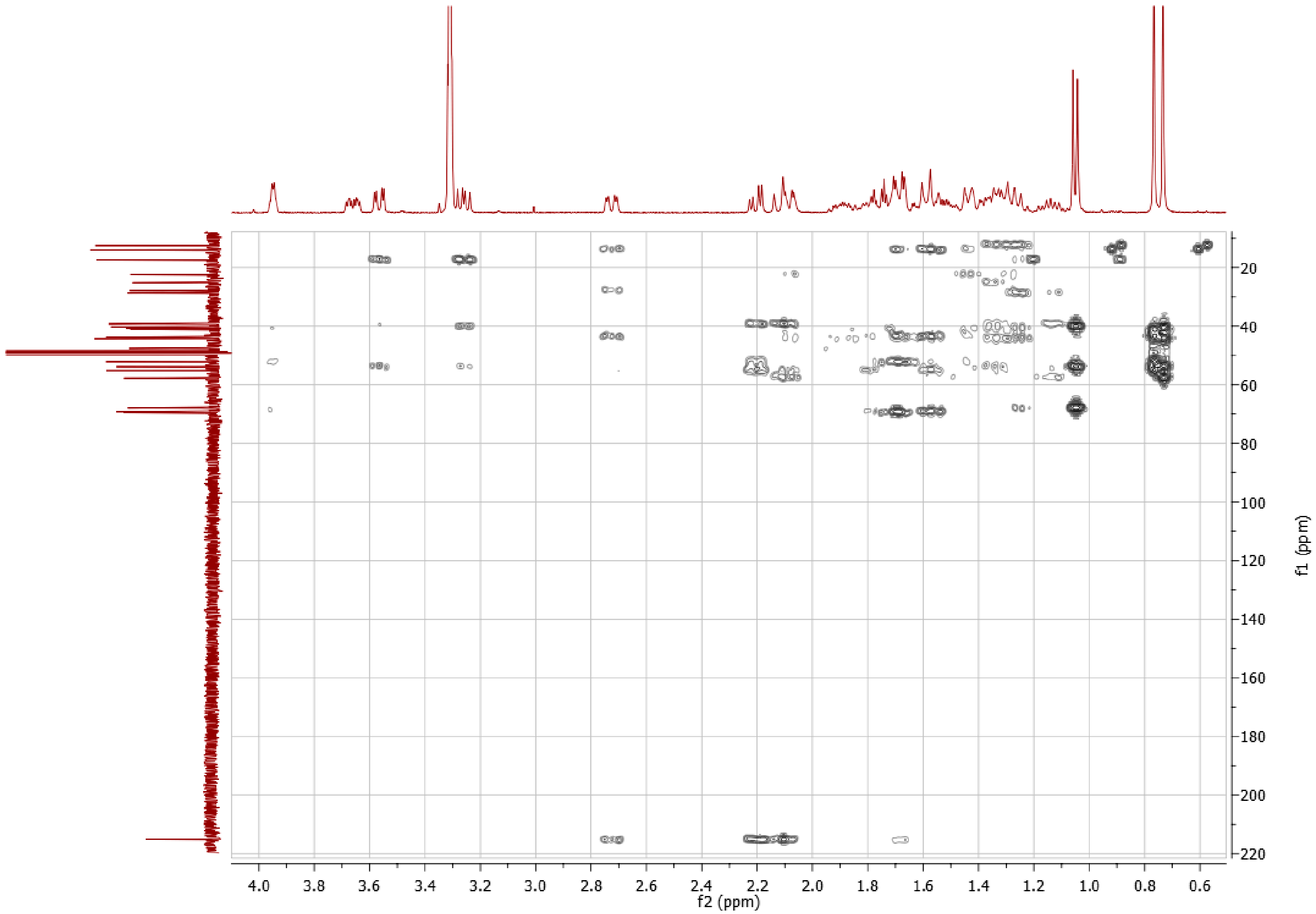

Figure S39. 2D HMBC NMR spectrum of 2α,3α,22-thrihydroxy-5α-cholan-23,24-dinor-6-one (**8**)

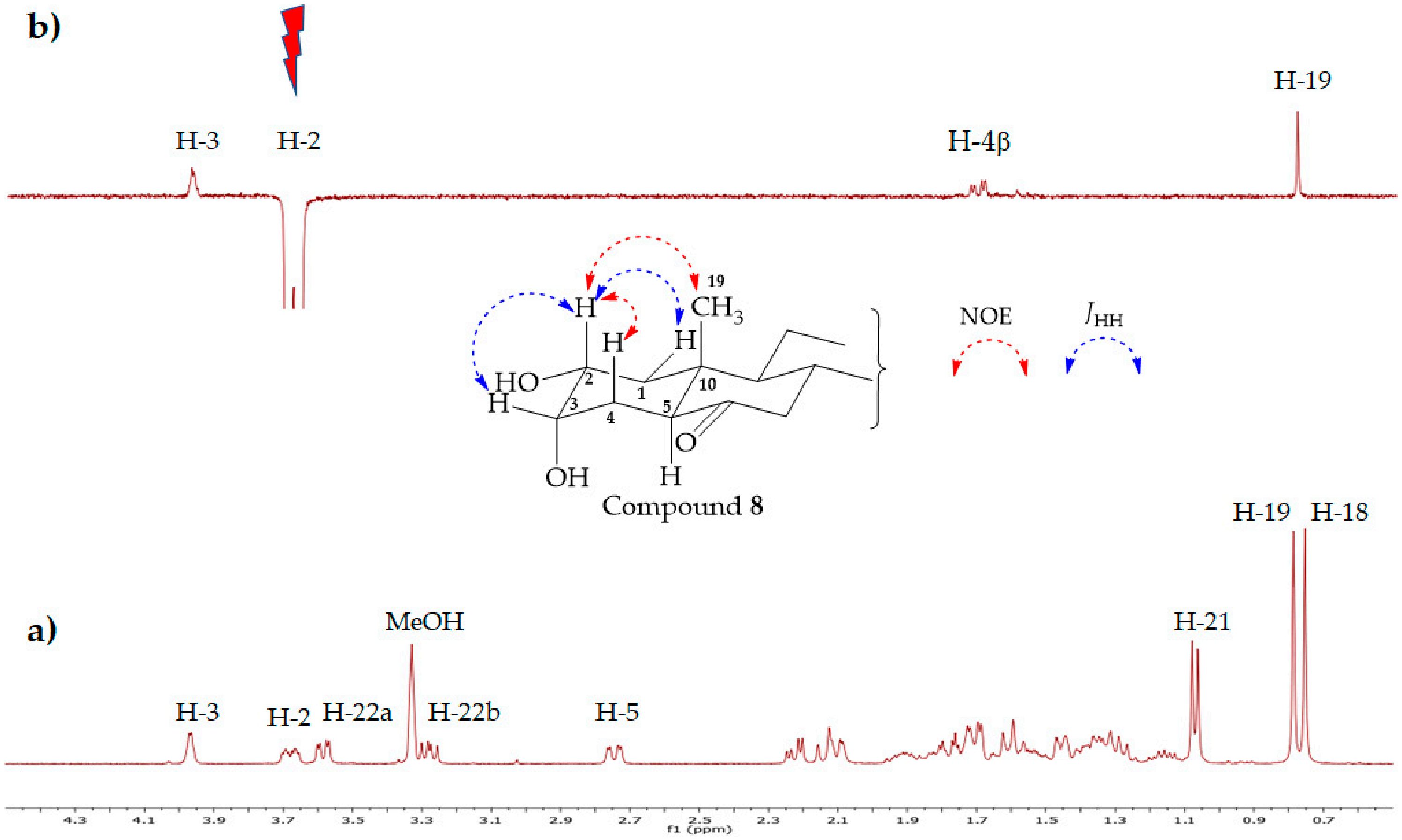

Figure S40. 1D selective NOESY NMR spectrum of 2*α*,3*α*,22-thrihydroxy-5*α*-cholan-23,24-dinor-6-one (**8**)

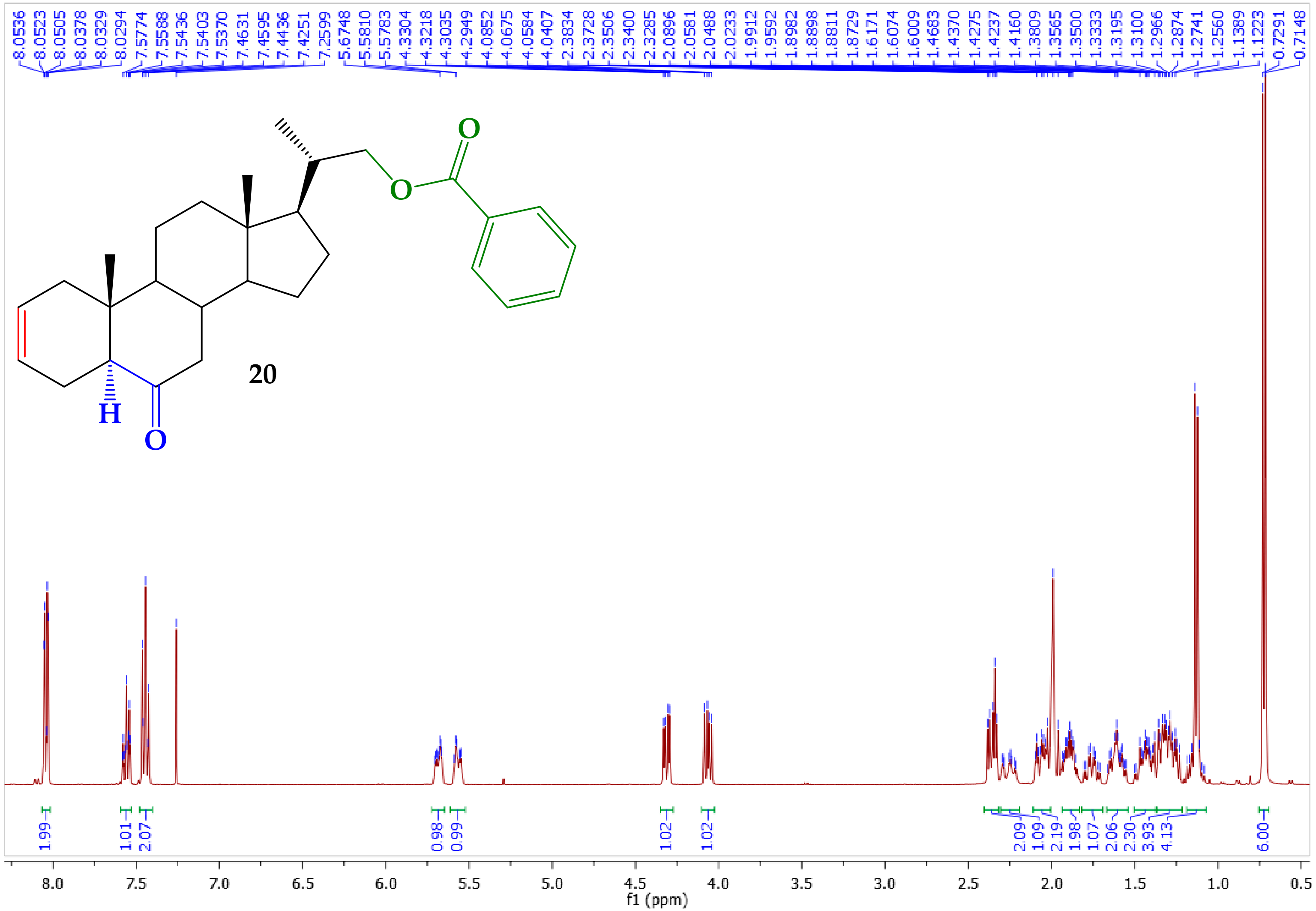

Figure S41. ^{1}H NMR spectrum of 5α-cholan-6-oxo-2-ene-23,24-dinor-22-benzoate-22-yl (**20**)

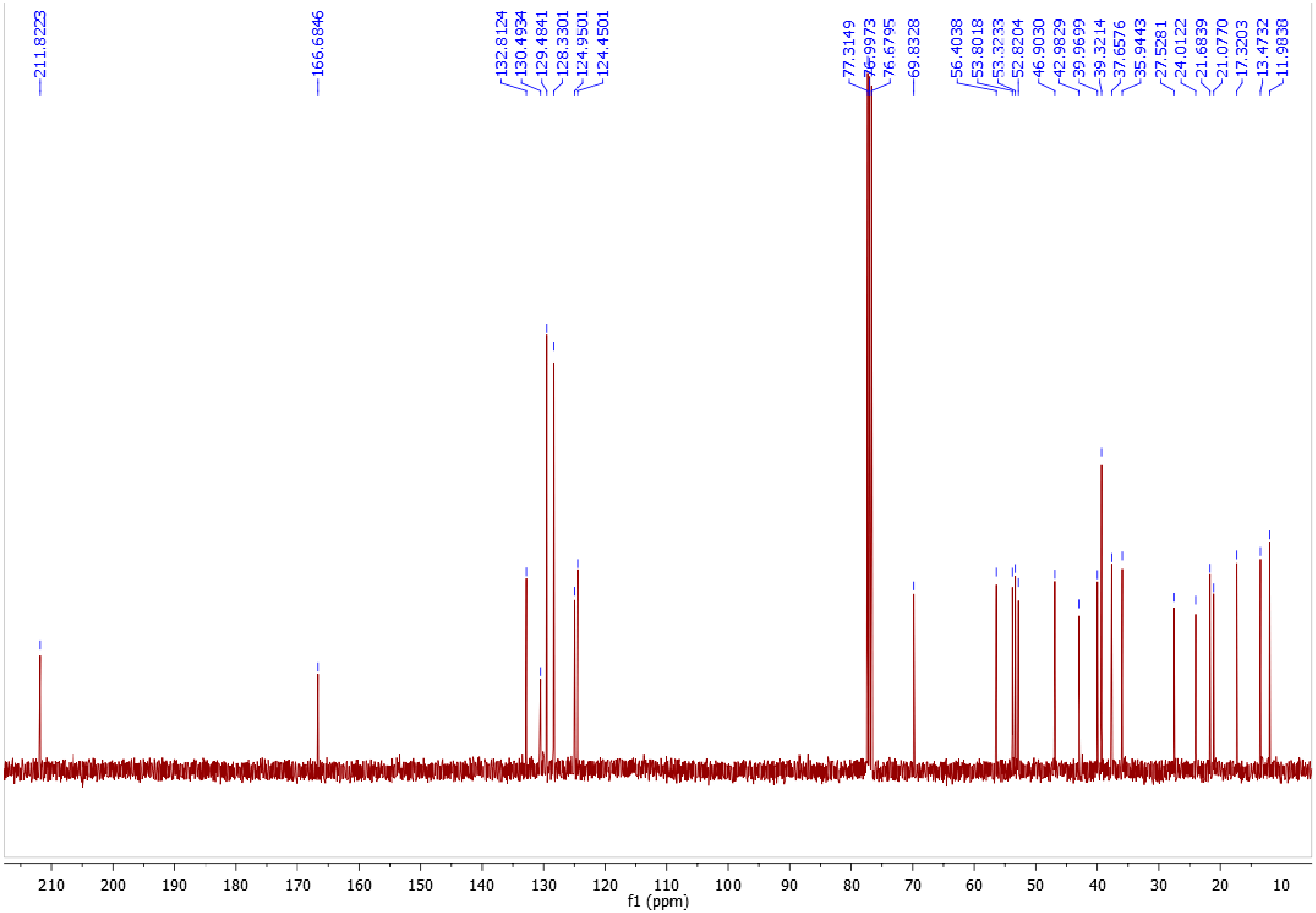

Figure S42. ^{13}C NMR spectrum of 5α-cholan-6-oxo-2-ene-23,24-dinor-22-benzoate-22-yl (**20**)

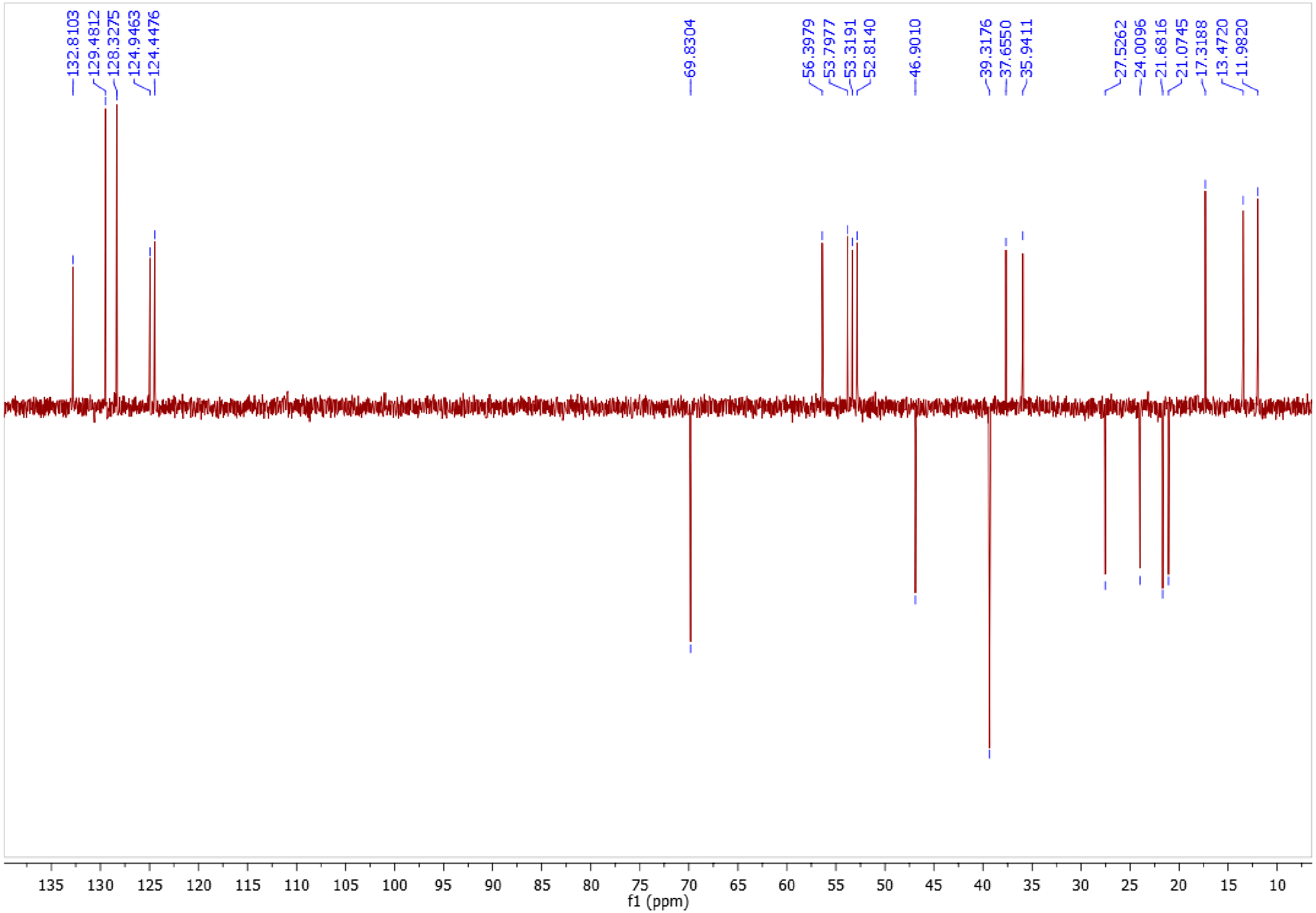

Figure S43. ^{13}C DEPT-135 NMR spectrum of 5α-cholan-6-oxo-2-ene-23,24-dinor-22-benzoate-22-yl (**20**)

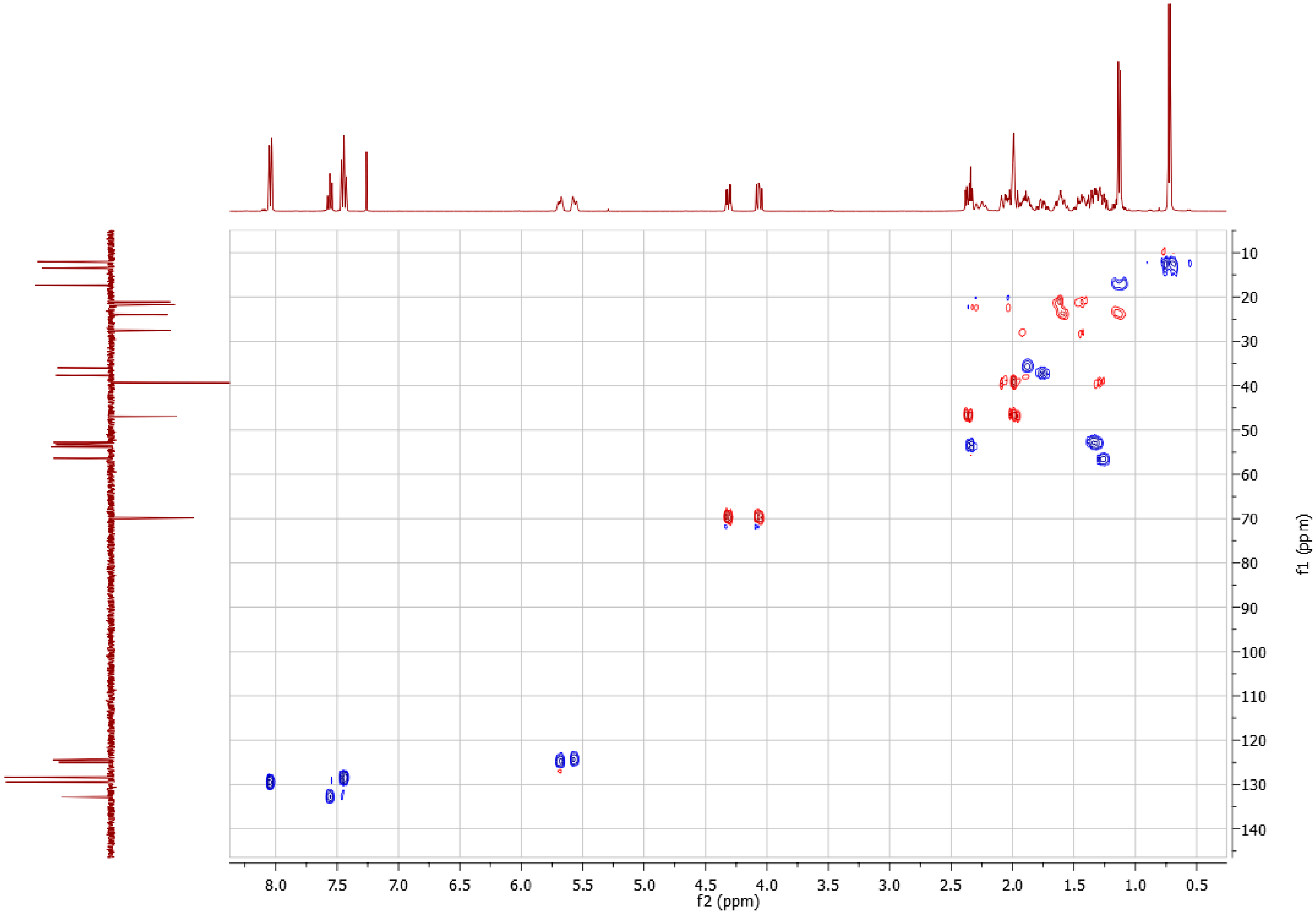

Figure S44. 2D HSQC NMR spectrum of 5α-cholan-6-oxo-2-ene-23,24-dinor-22-benzoate-22-yl (**20**)

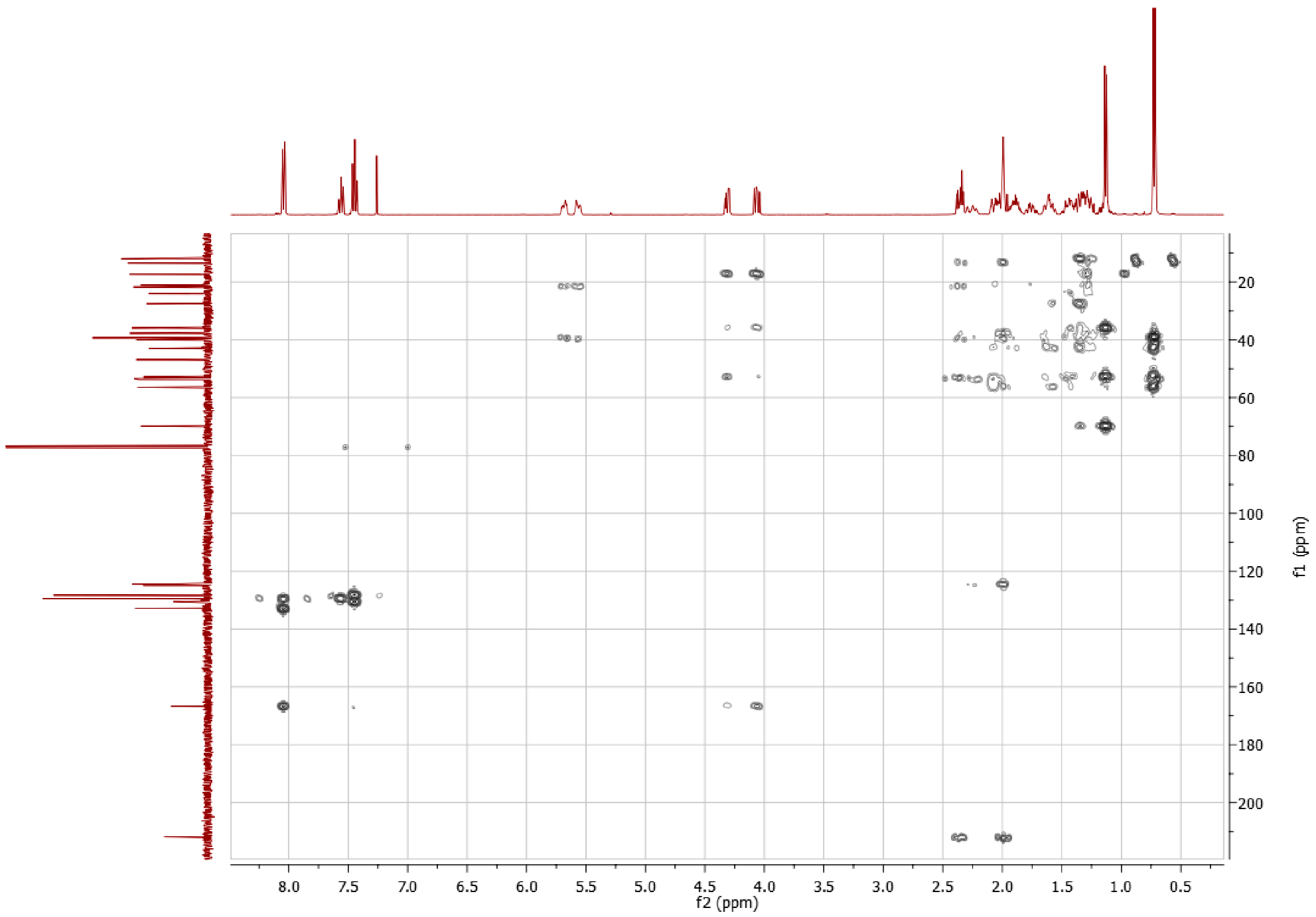

Figure S44. 2D HMBC NMR spectrum of 5α-cholan-6-oxo-2-ene-23,24-dinor-22-benzoate-22-yl (**20**)

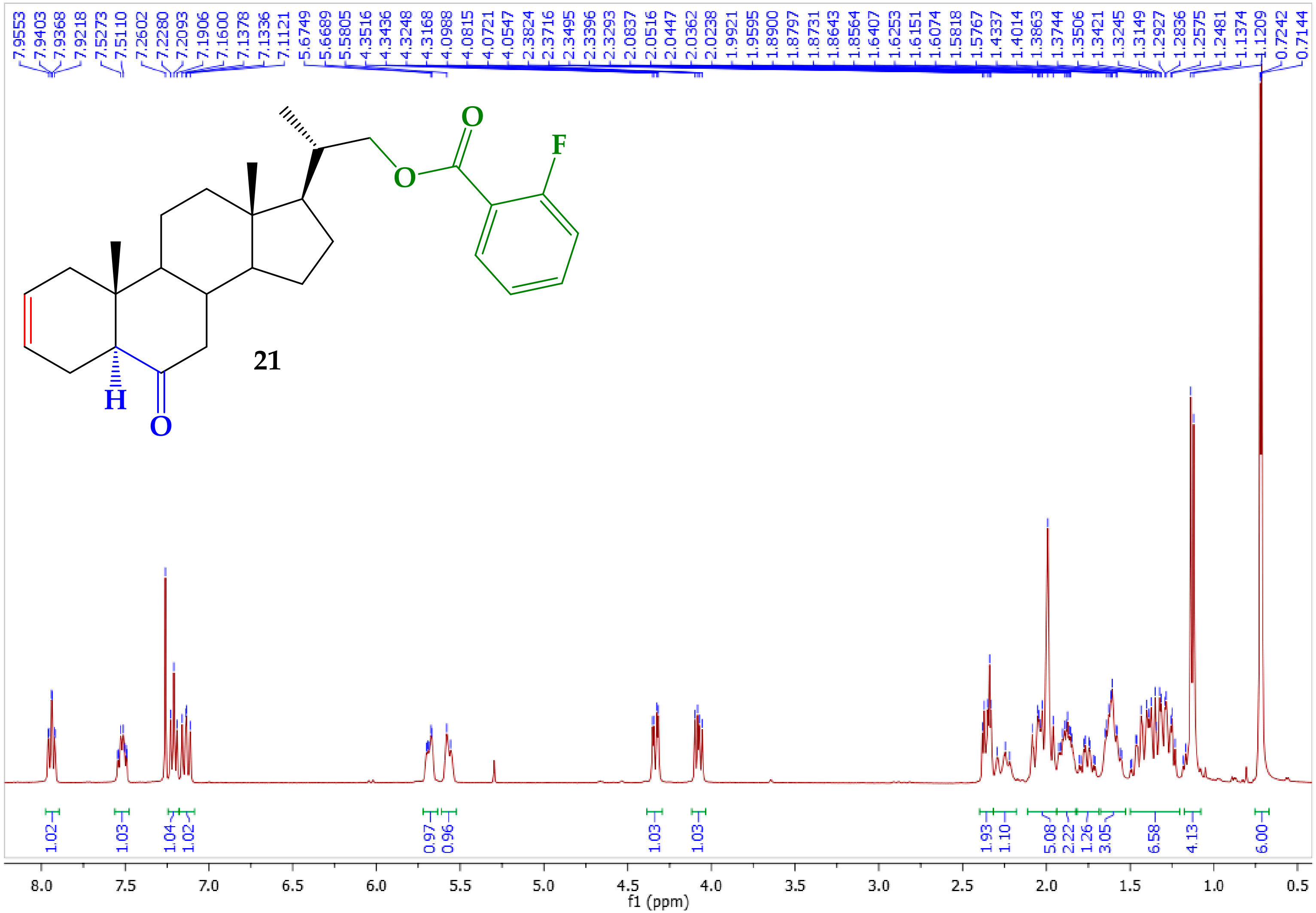

Figure S46. ^{1}H NMR spectrum of *5α-cholan-6-oxo-2-ene-23,24-dinor-22-(2-Fluoro) benzoate-22-yl* (***21***)

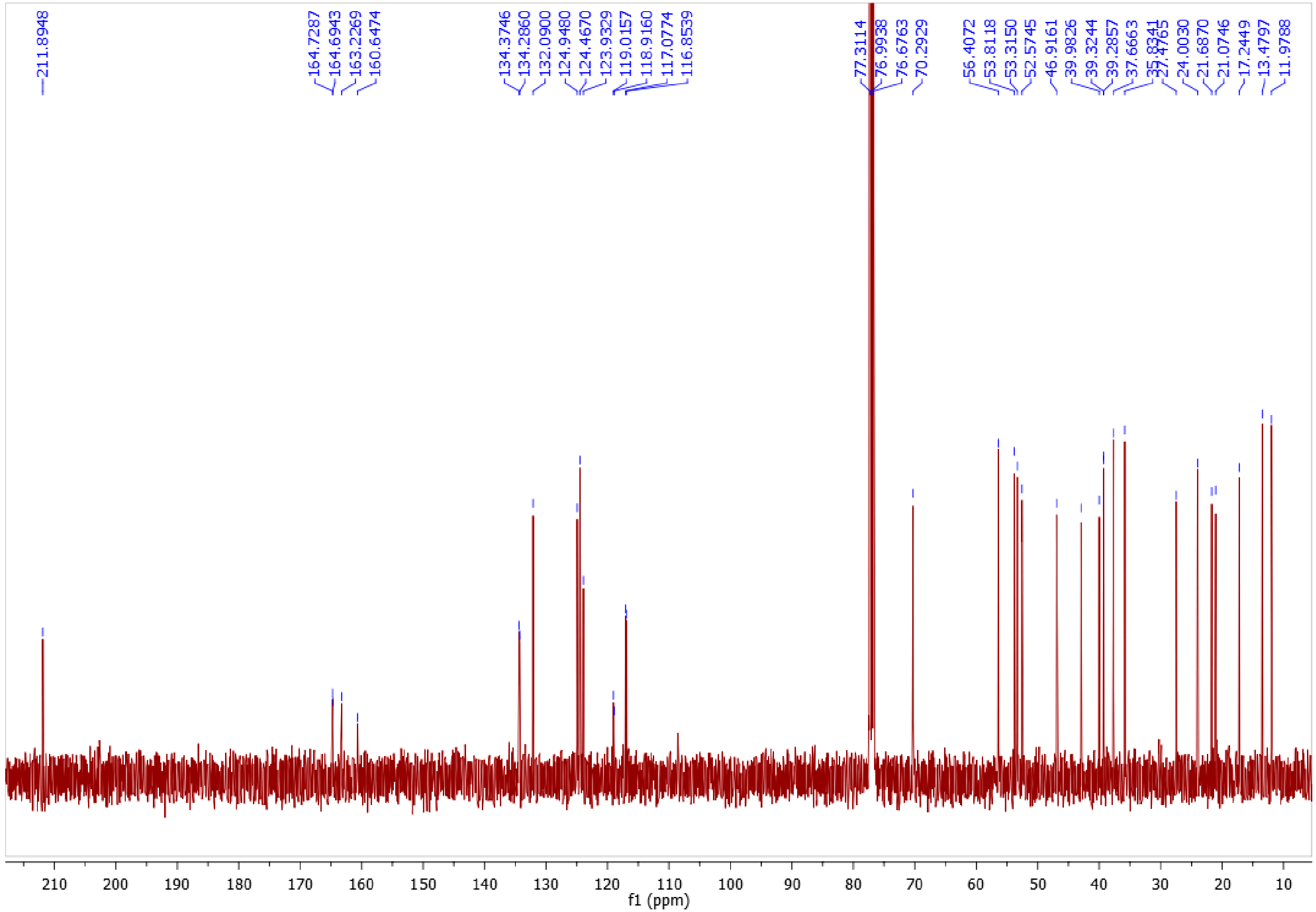

Figure S47. ^{13}C NMR spectrum of 5α-cholan-6-oxo-2-ene-23,24-dinor-22-(2-Fluoro) benzoate-22-yl (**21**)

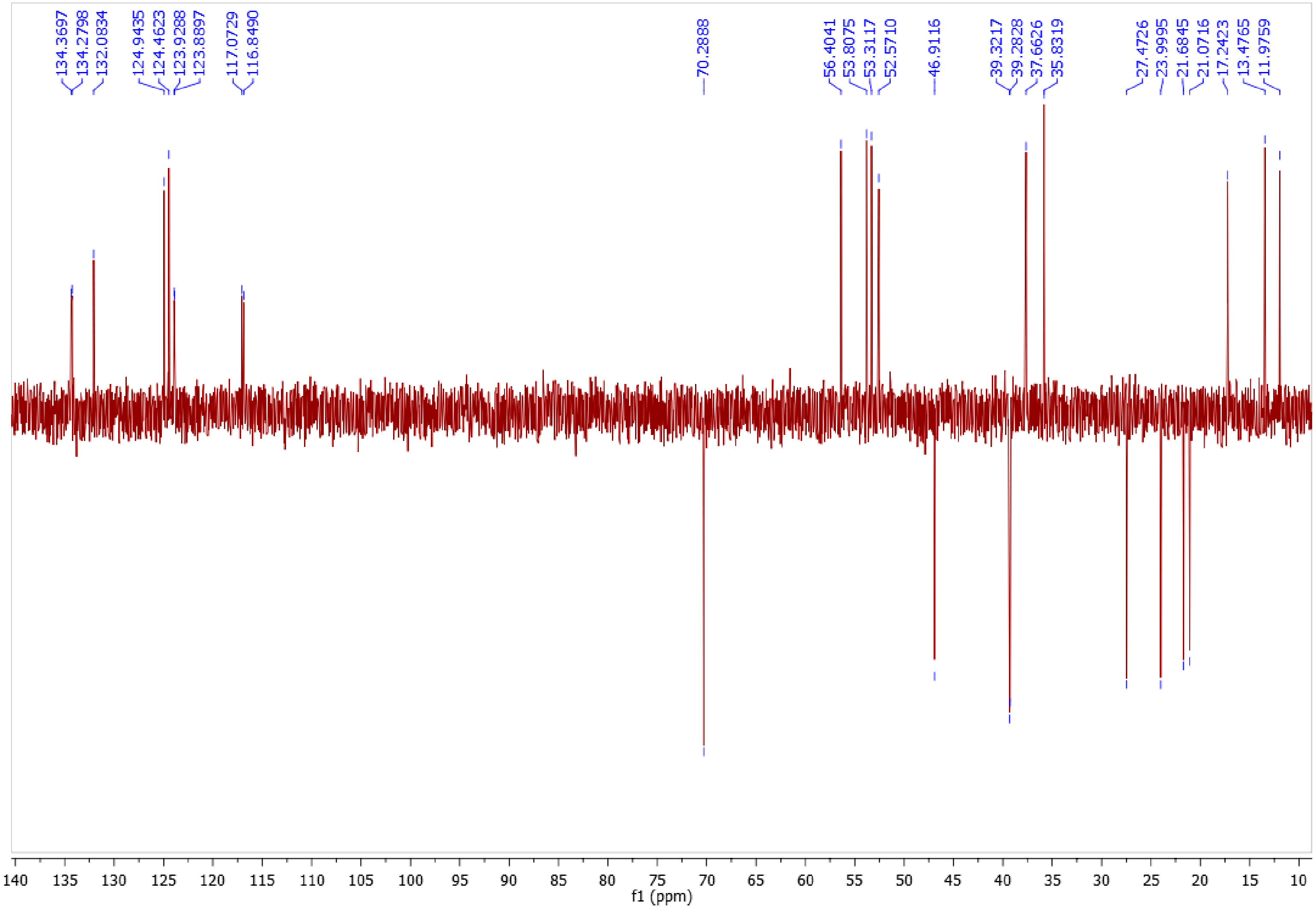

Figure S48. ^{13}C DEPT-135 NMR spectrum of 5α-cholan-6-oxo-2-ene-23,24-dinor-22-(2-Fluoro) benzoate-22-yl (**21**)

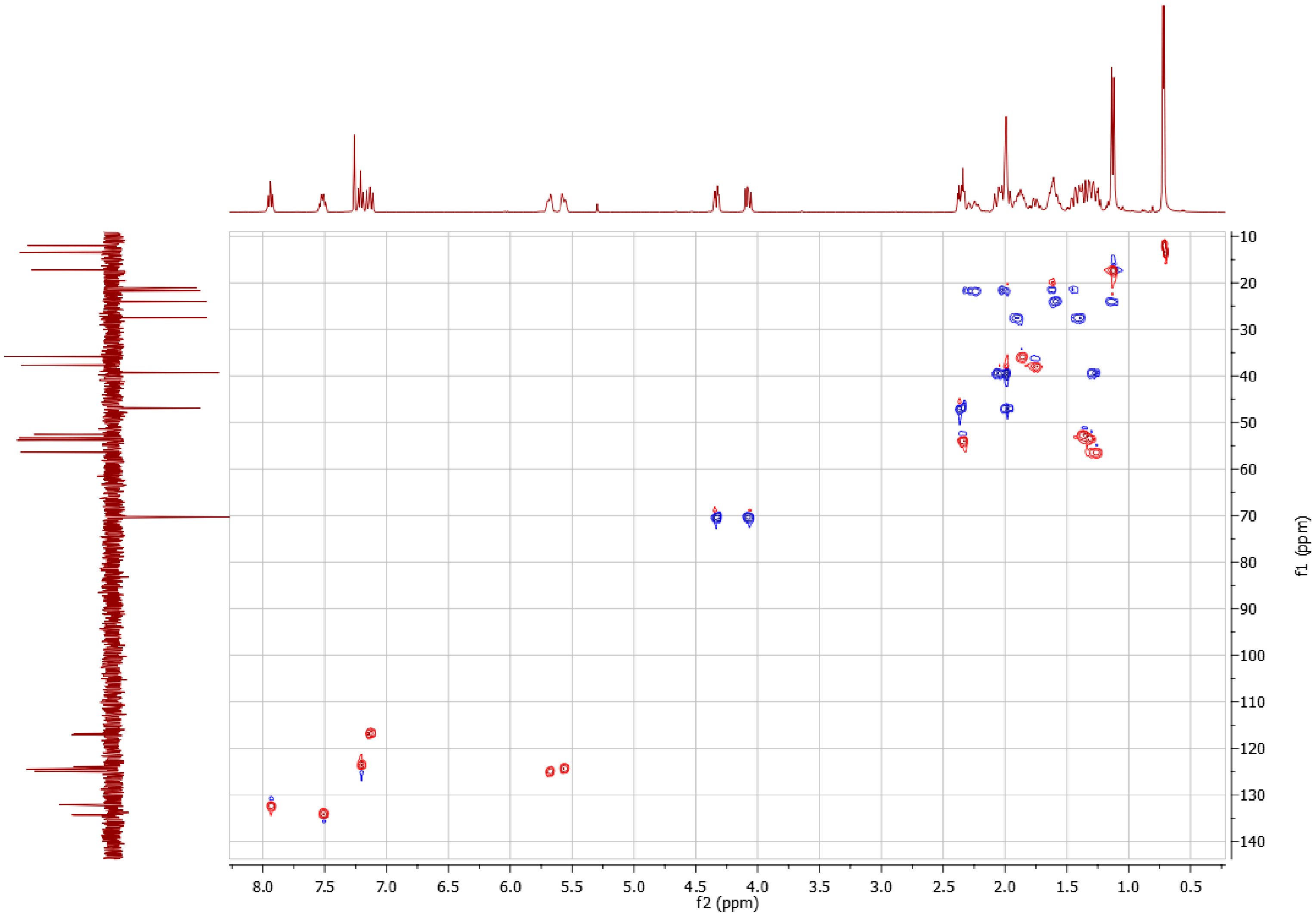

Figure S49. 2D HSQC NMR spectrum of 5α-cholan-6-oxo-2-ene-23,24-dinor-22-(2-Fluoro) benzoate-22-yl (**21**)

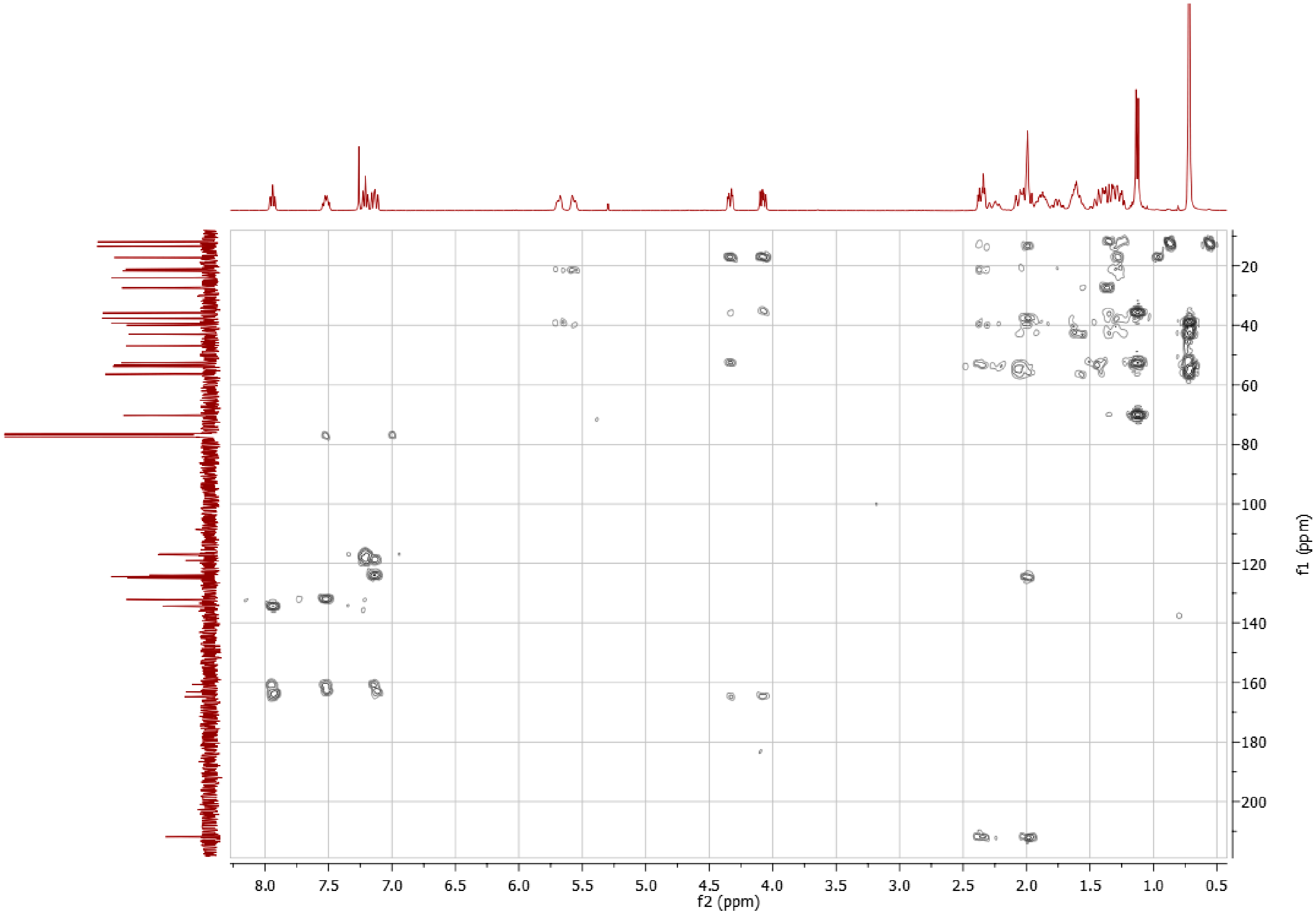

Figure S50. 2D HMBC NMR spectrum of 5α-cholan-6-oxo-2-ene-23,24-dinor-22-(2-Fluoro) benzoate-22-yl (**21**)

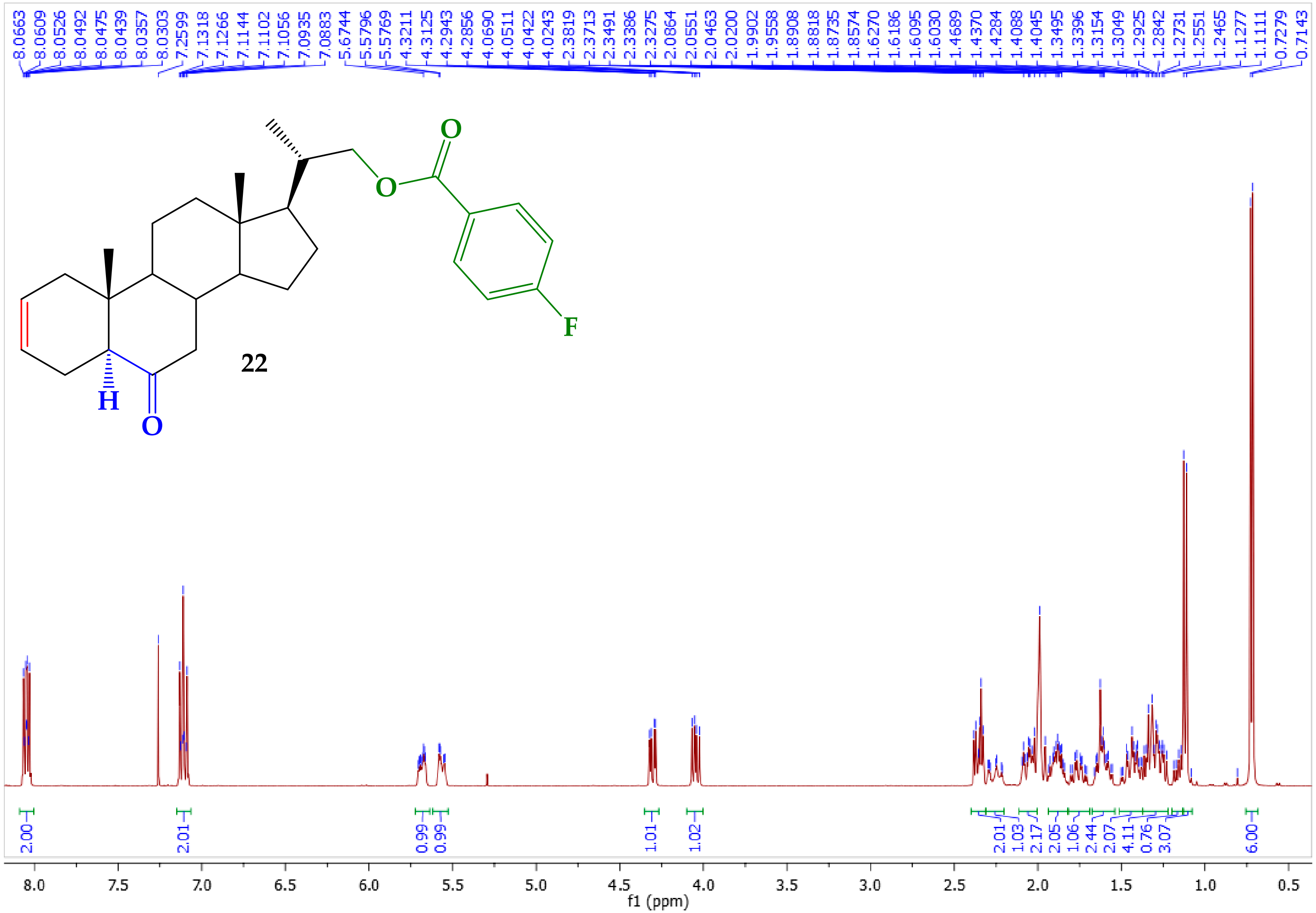

Figure S51. ^{1}H NMR spectrum of 5α-cholan-6-oxo-2-ene-23,24-dinor-22-(4-Fluoro)-benzoate-22-yl (**22**)

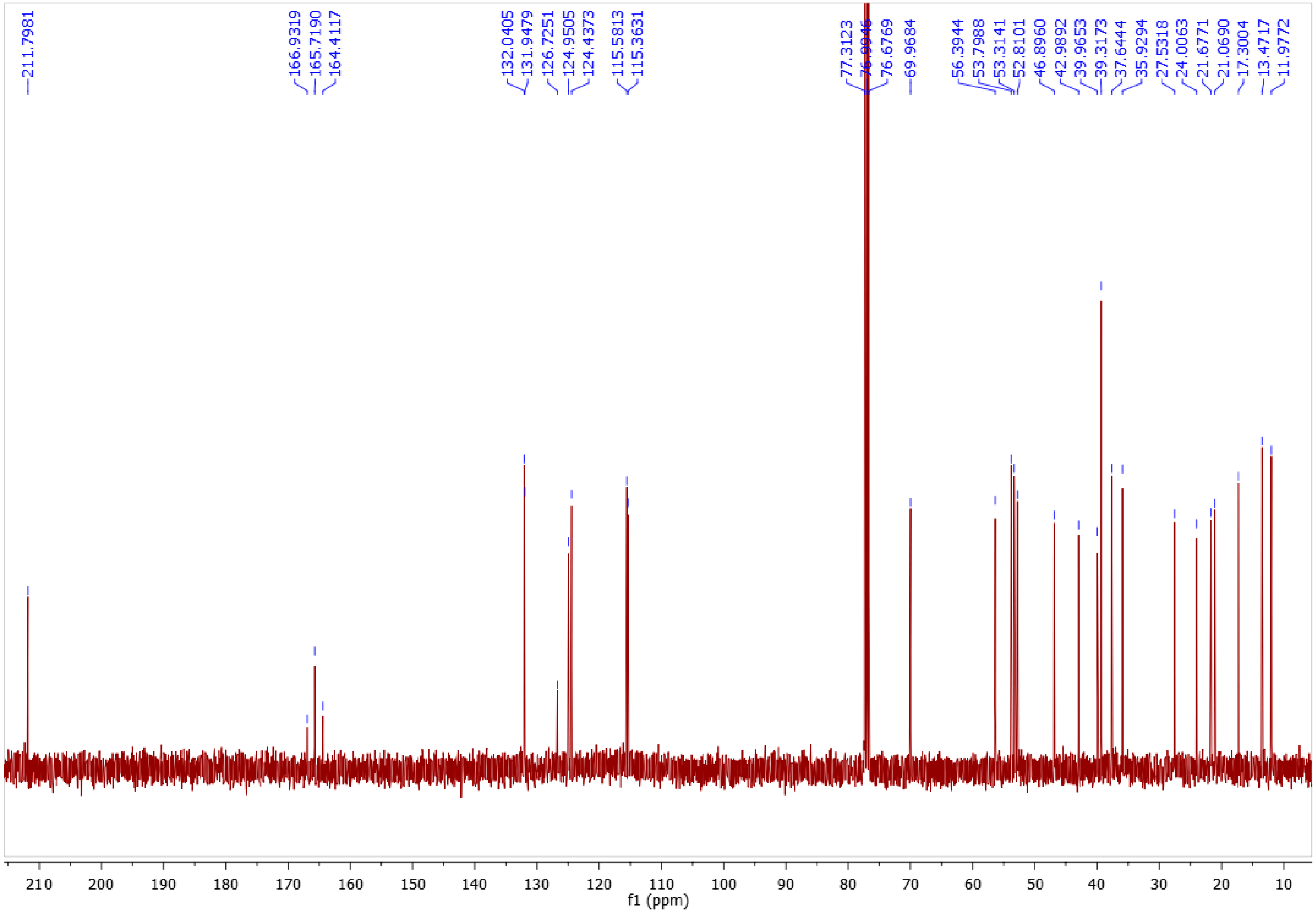

Figure S52. ^{13}C NMR spectrum of 5α-cholan-6-oxo-2-ene-23,24-dinor-22-(4-Fluoro)-benzoate-22-yl (**22**)

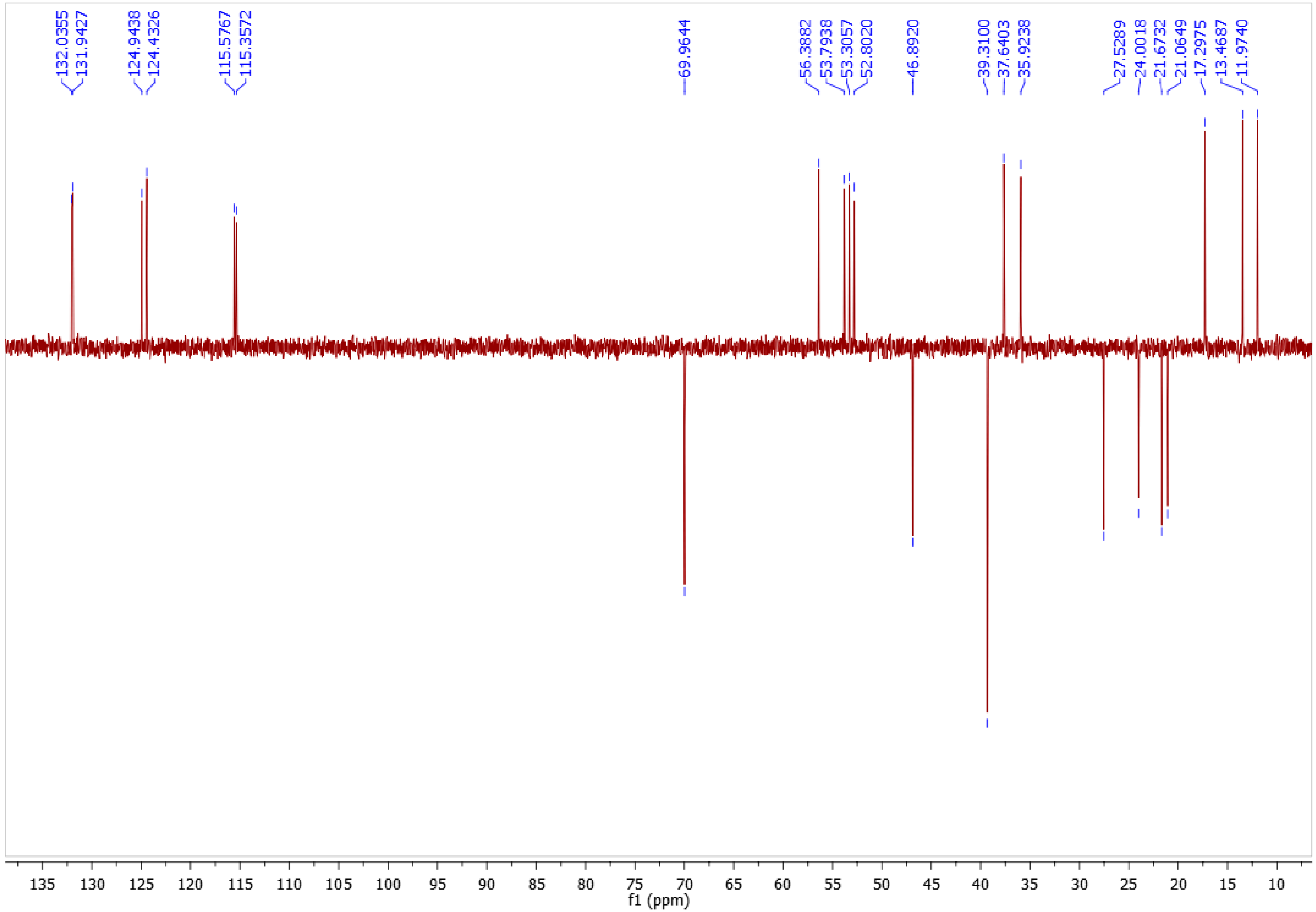

Figure S53. ^{13}C DEPT-135 NMR spectrum of 5α-cholan-6-oxo-2-ene-23,24-dinor-22-(4-Fluoro)-benzoate-22-yl (**22**)

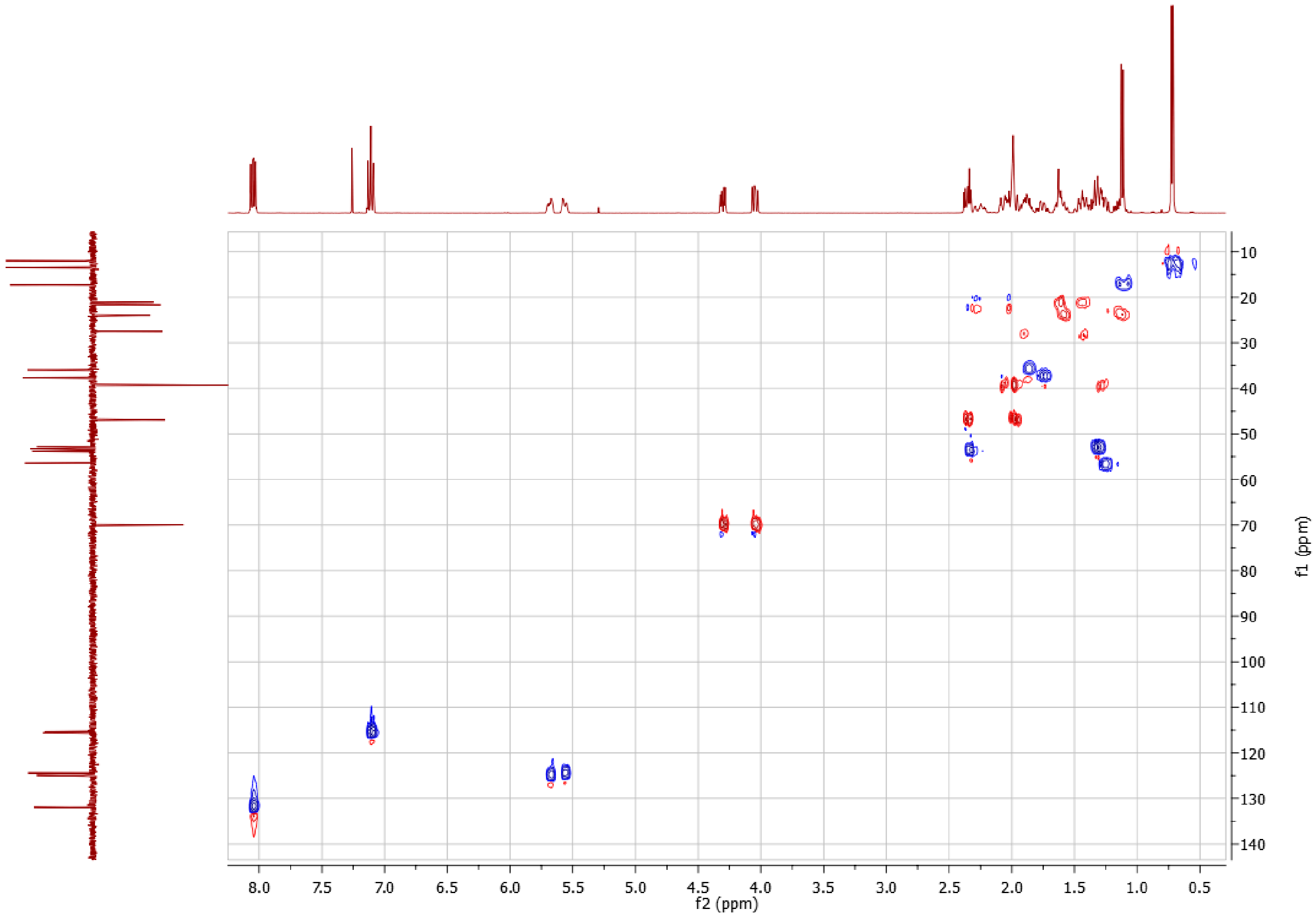

Figure S54. 2D HSQC NMR spectrum of 5α-cholan-6-oxo-2-ene-23,24-dinor-22-(4-Fluoro)-benzoate-22-yl (**22**)

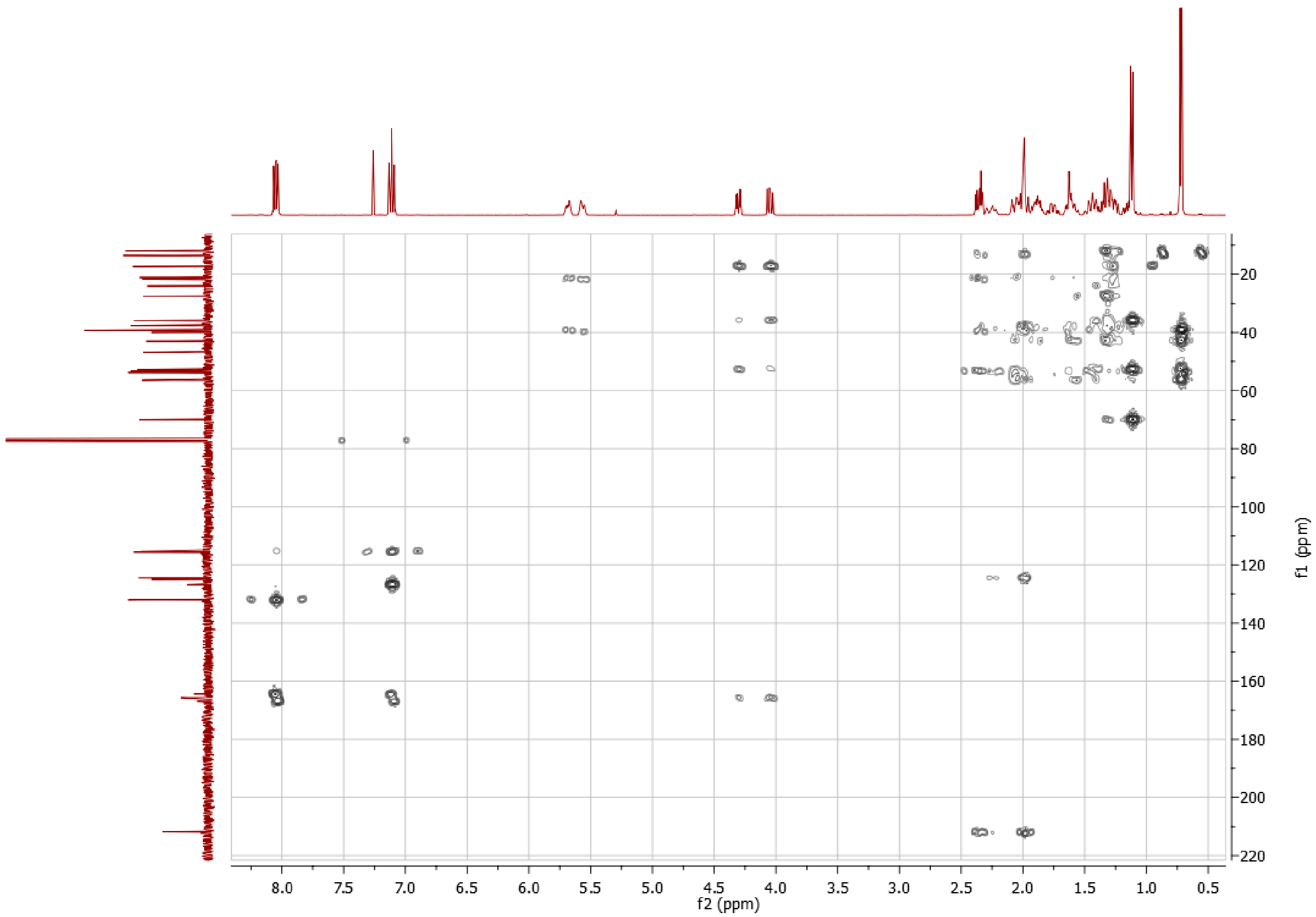

Figure S55. 2D HMBC NMR spectrum of 5α-cholan-6-oxo-2-ene-23,24-dinor-22-(4-Fluoro)-benzoate-22-yl (**22**)

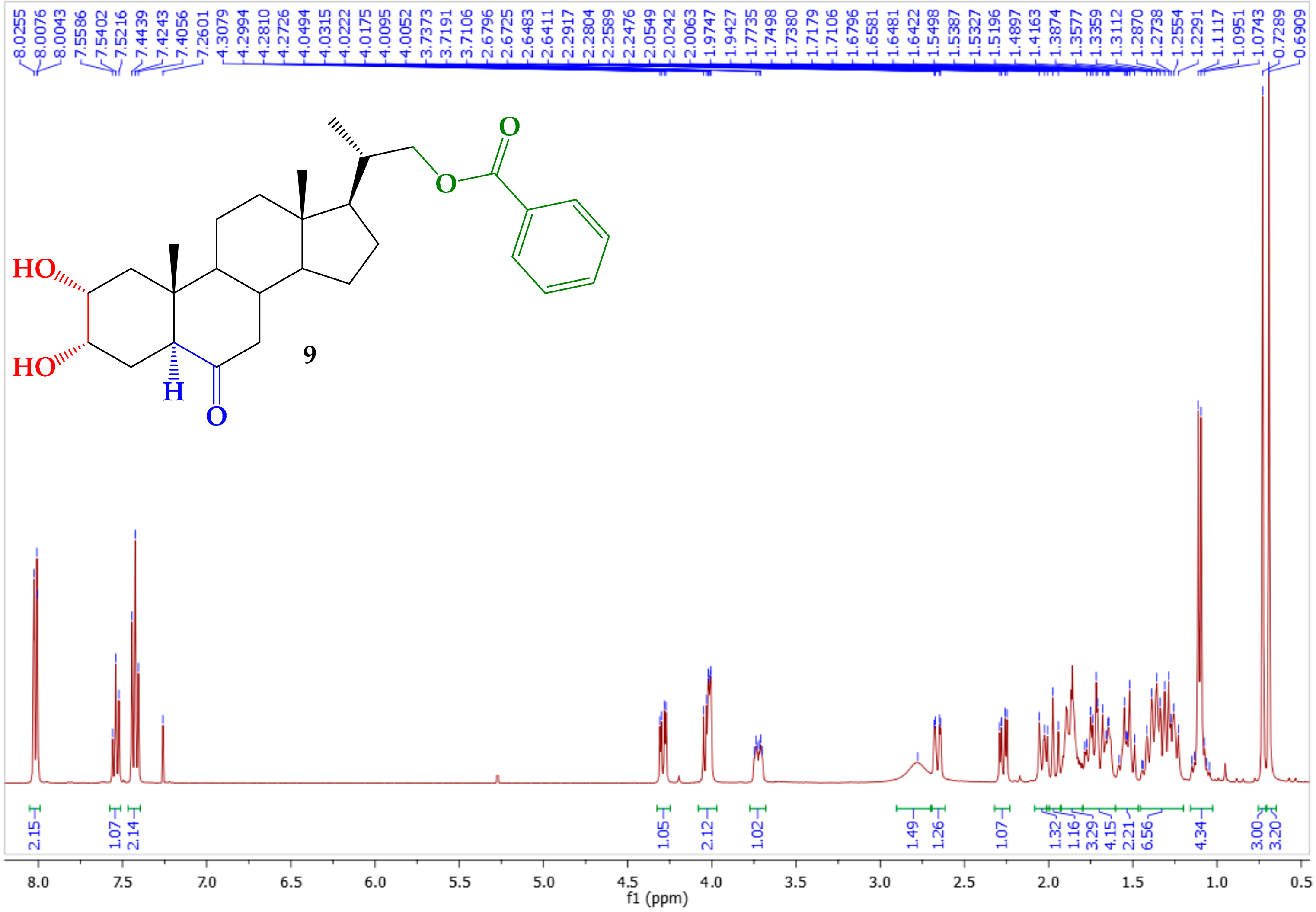

Figure S56. ^{1}H NMR spectrum of 2α,3α-dihydroxy-5α-cholan-6-oxo-23,24-dinor-22-benzoate-22-yl (**9**)

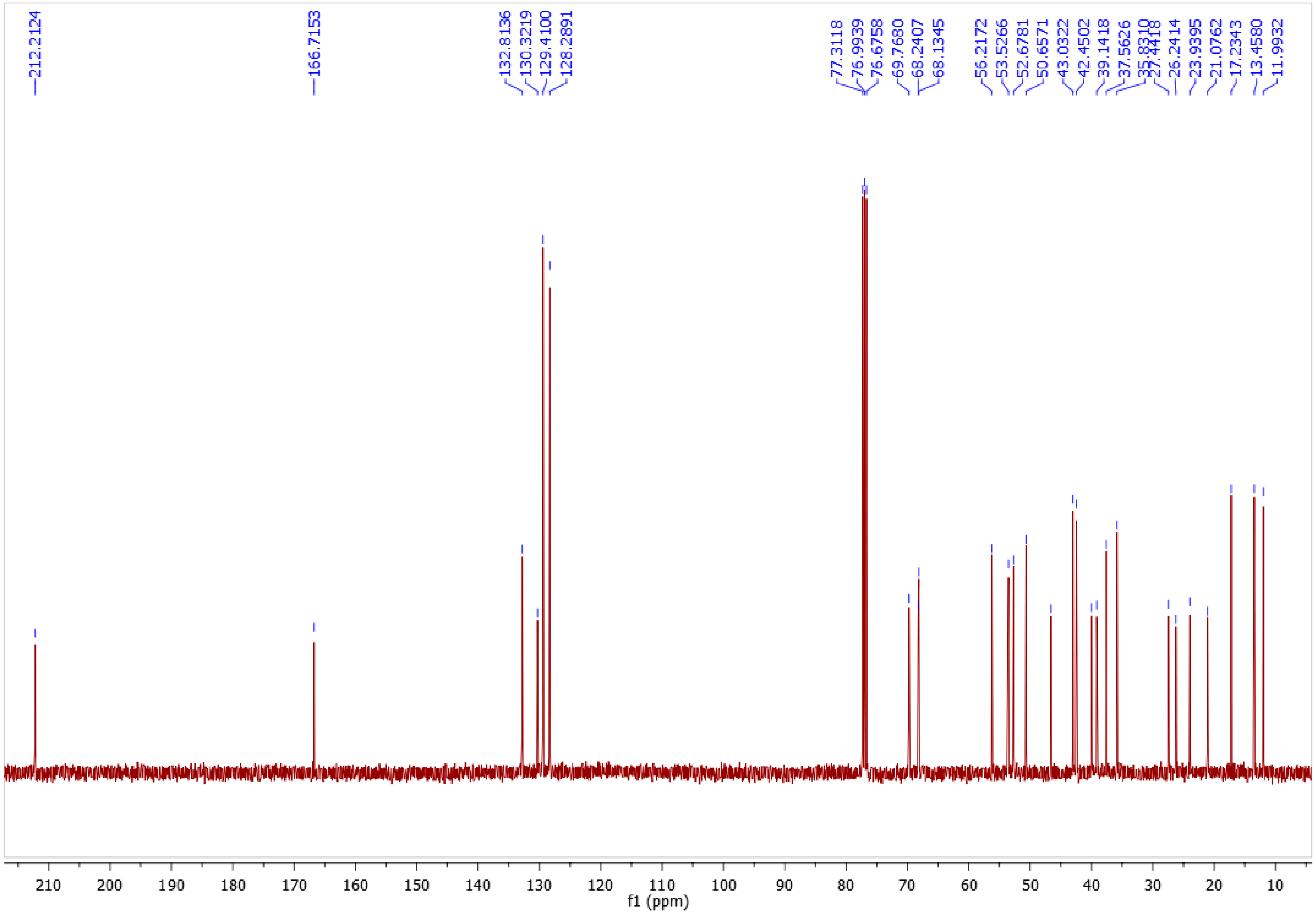

Figure S57. ^{13}C NMR spectrum of 2α,3α-dihydroxy-5α-cholan-6-oxo-23,24-dinor-22-benzoate-22-yl (**9**)

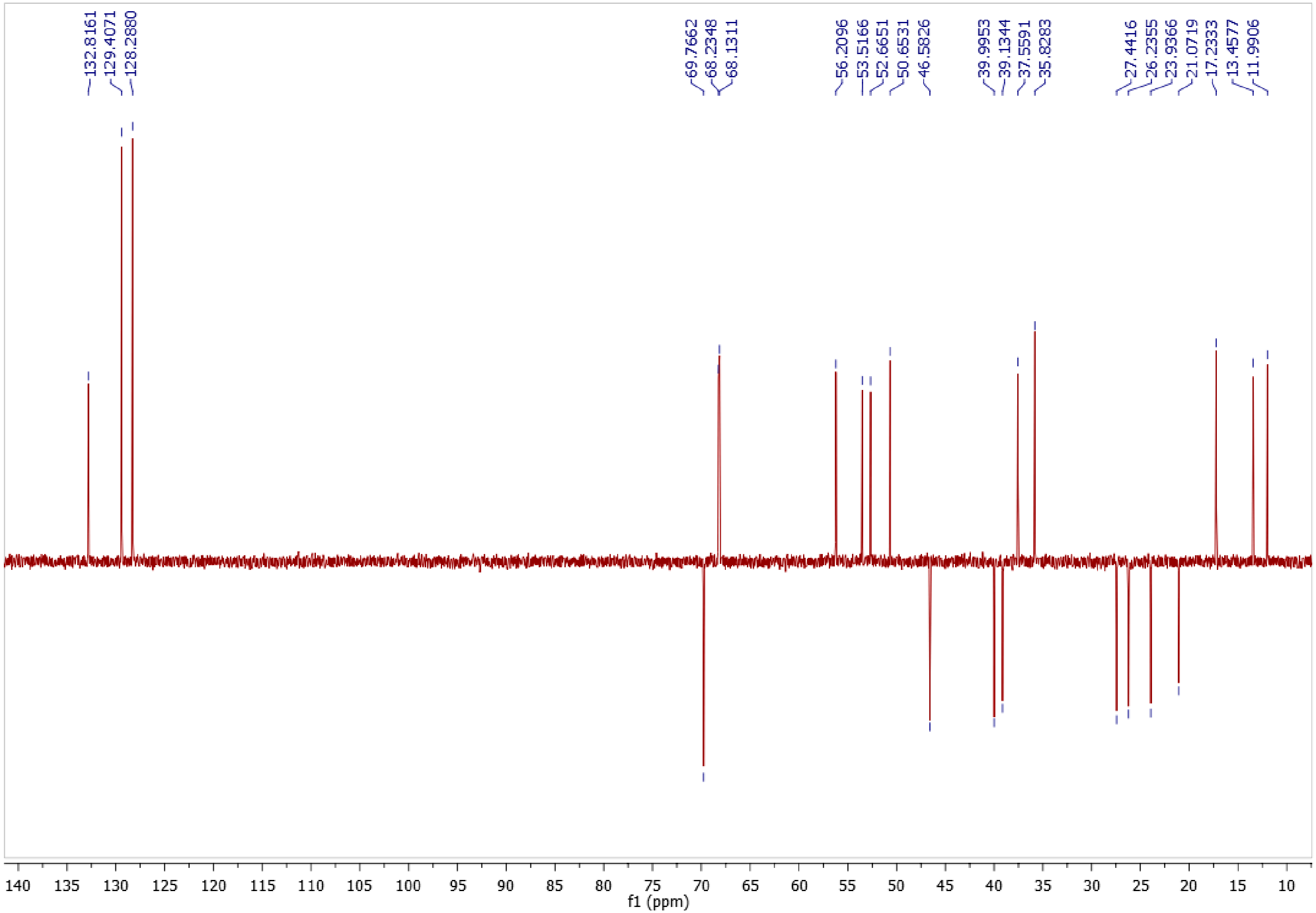

Figure S58. ^{13}C DEPT-135 NMR spectrum of 2α,3α-dihydroxy-5α-cholan-6-oxo-23,24-dinor-22-benzoate-22-yl (**9**)

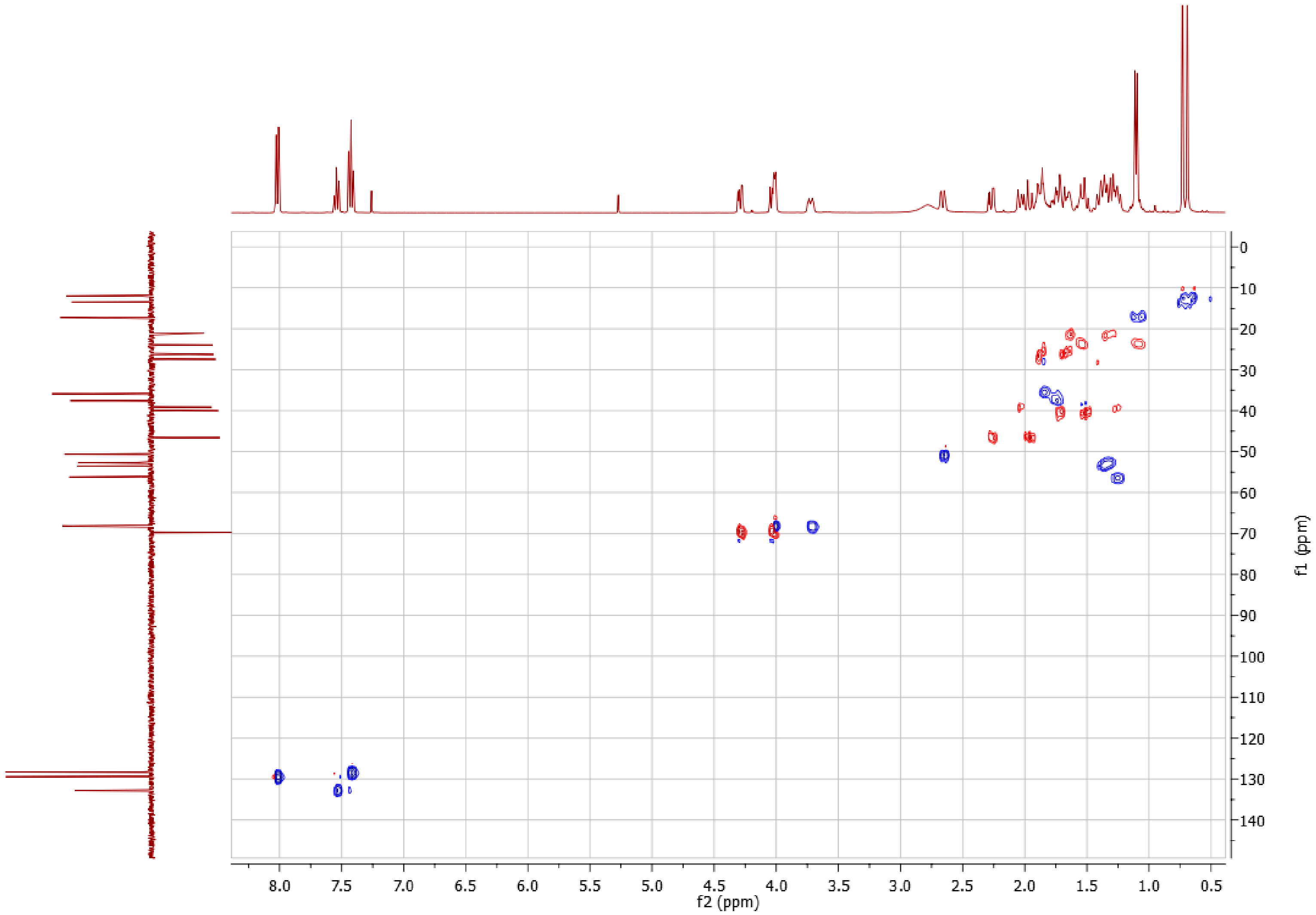

Figure S59. 2D HSQC NMR spectrum of 2α,3α-dihydroxy-5α-cholan-6-oxo-23,24-dinor-22-benzoate-22-yl (**9**)

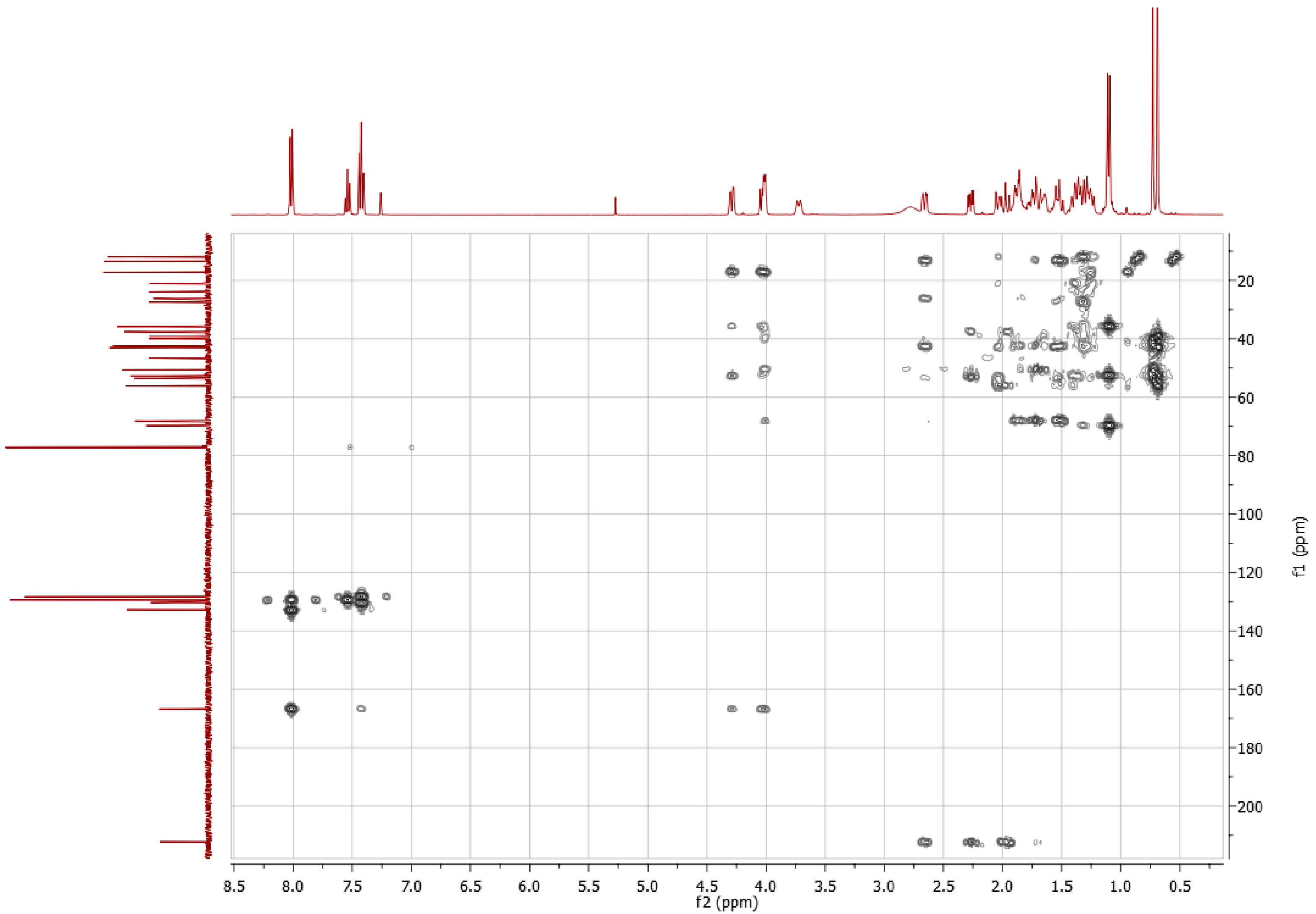

Figure S60. 2D HMBC NMR spectrum of 2α,3α-dihydroxy-5α-cholan-6-oxo-23,24-dinor-22-benzoate-22-yl (**9**)

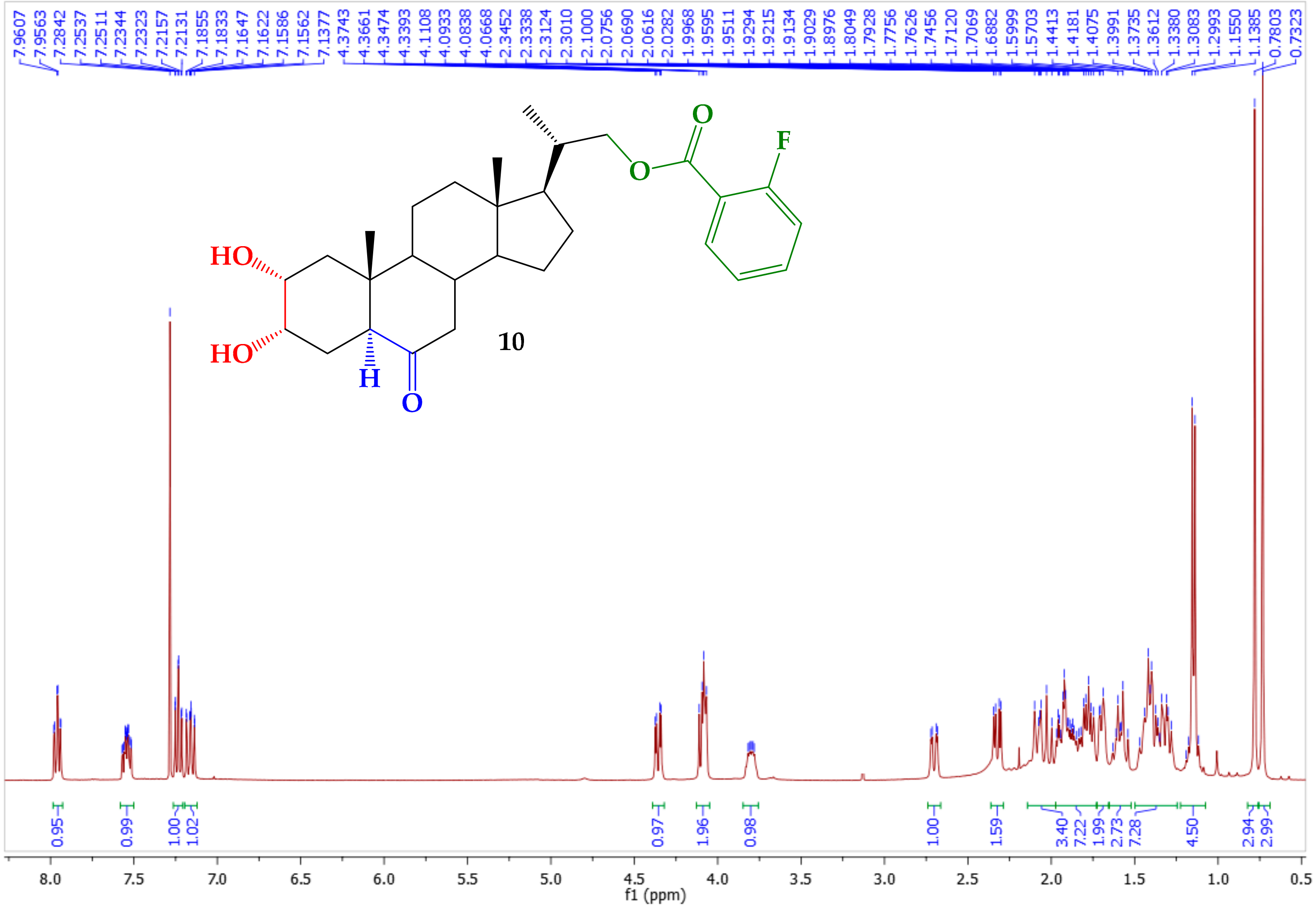

Figure S61. ^{1}H NMR spectrum of 2α,3α-dihydroxy-5α-cholan-6-oxo-23,24-dinor-22-(2-Fluoro)-benzoate-22-yl (**10**)

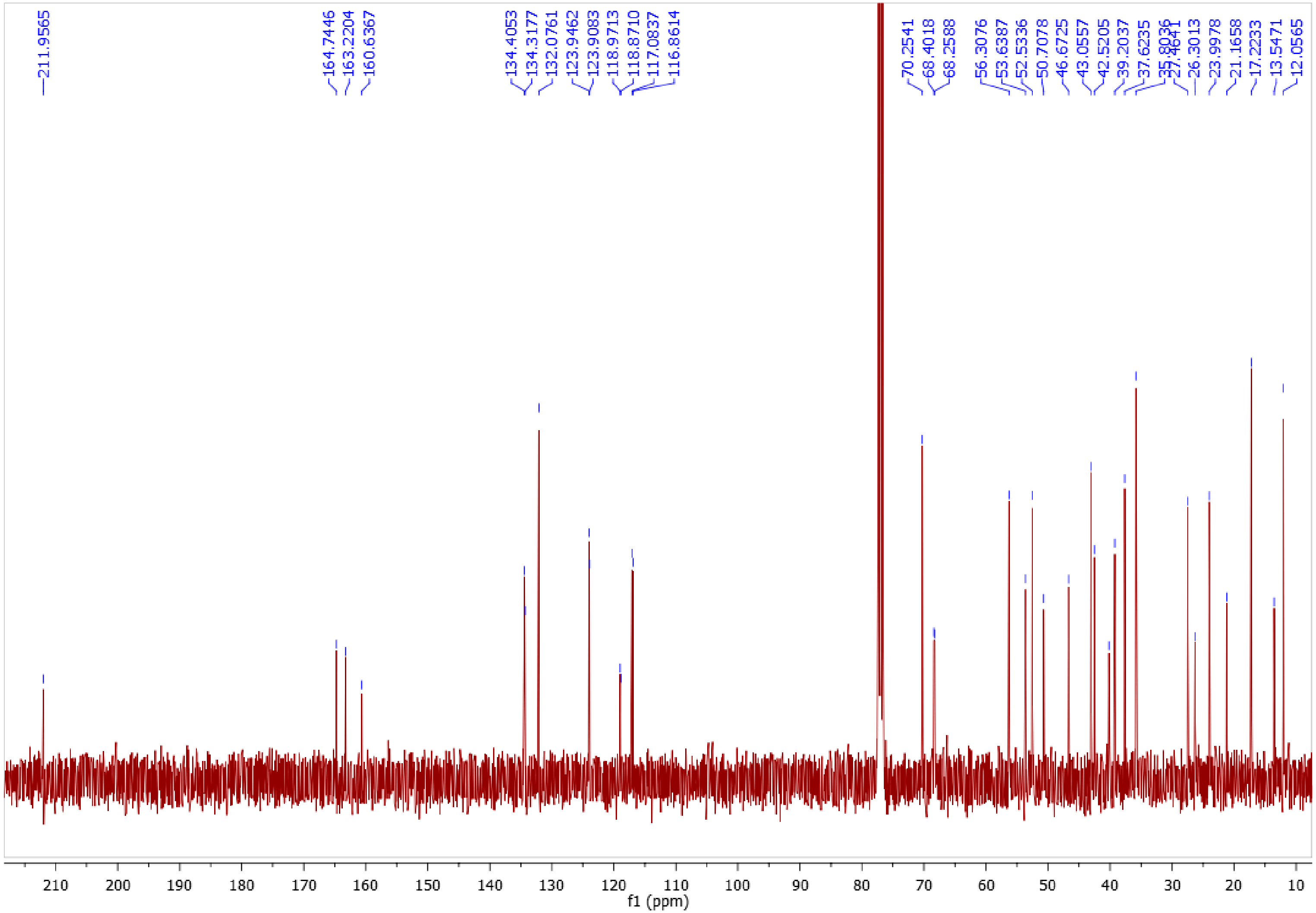

Figure S62. ^{13}C NMR spectrum of 2α,3α-dihydroxy-5α-cholan-6-oxo-23,24-dinor-22-(2-Fluoro)-benzoate-22-yl (**10**)

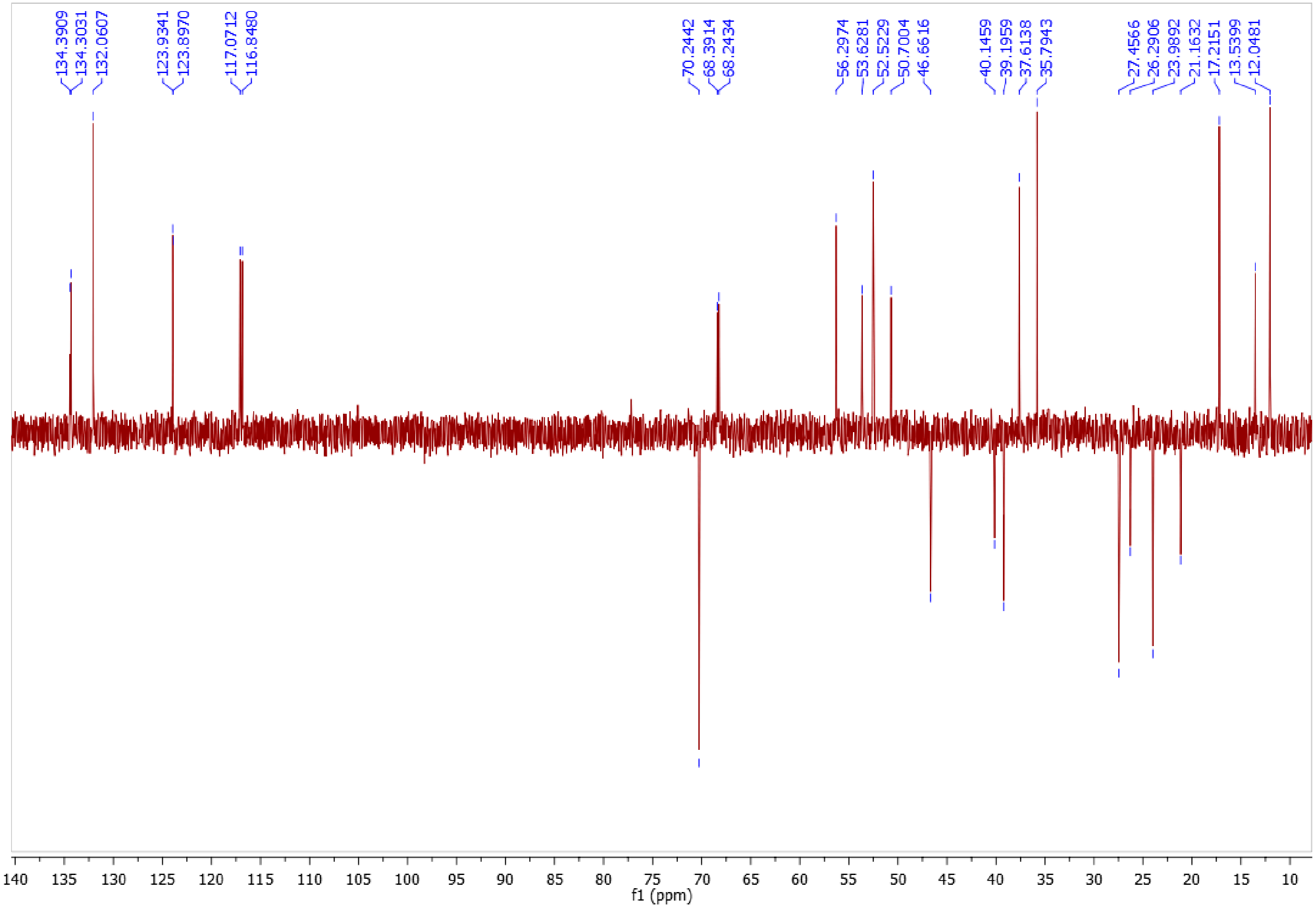

Figure S63. ^{13}C DEPT-135 NMR spectrum of 2*α*,3*α*-dihydroxy-5*α*-cholan-6-oxo-23,24-dinor-22-(2-Fluoro)-benzoate-22-yl (**10**)

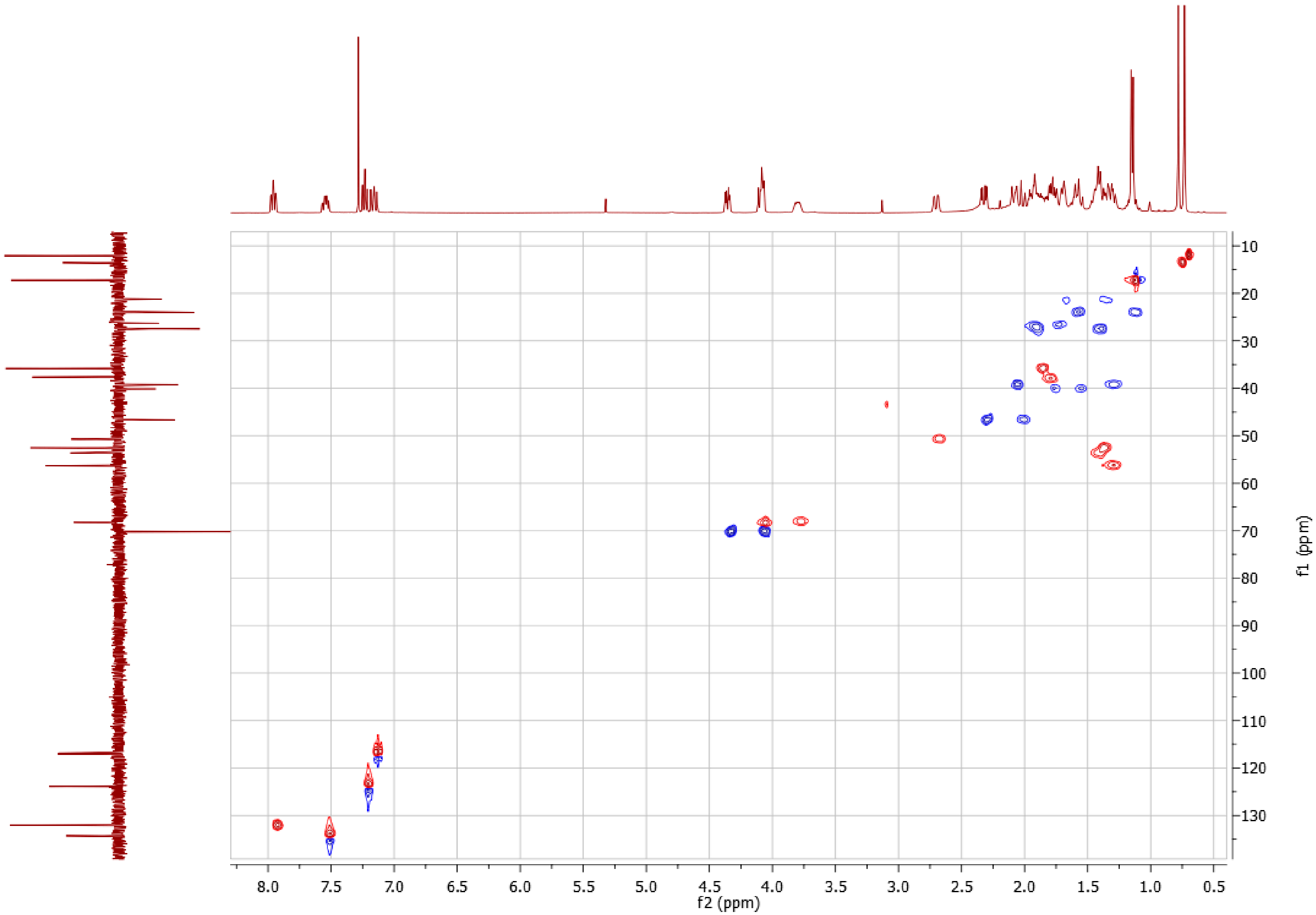

Figure S64. 2D HSQC NMR spectrum of 2α,3α-dihydroxy-5α-cholan-6-oxo-23,24-dinor-22-(2-Fluoro)-benzoate-22-yl (**10**)

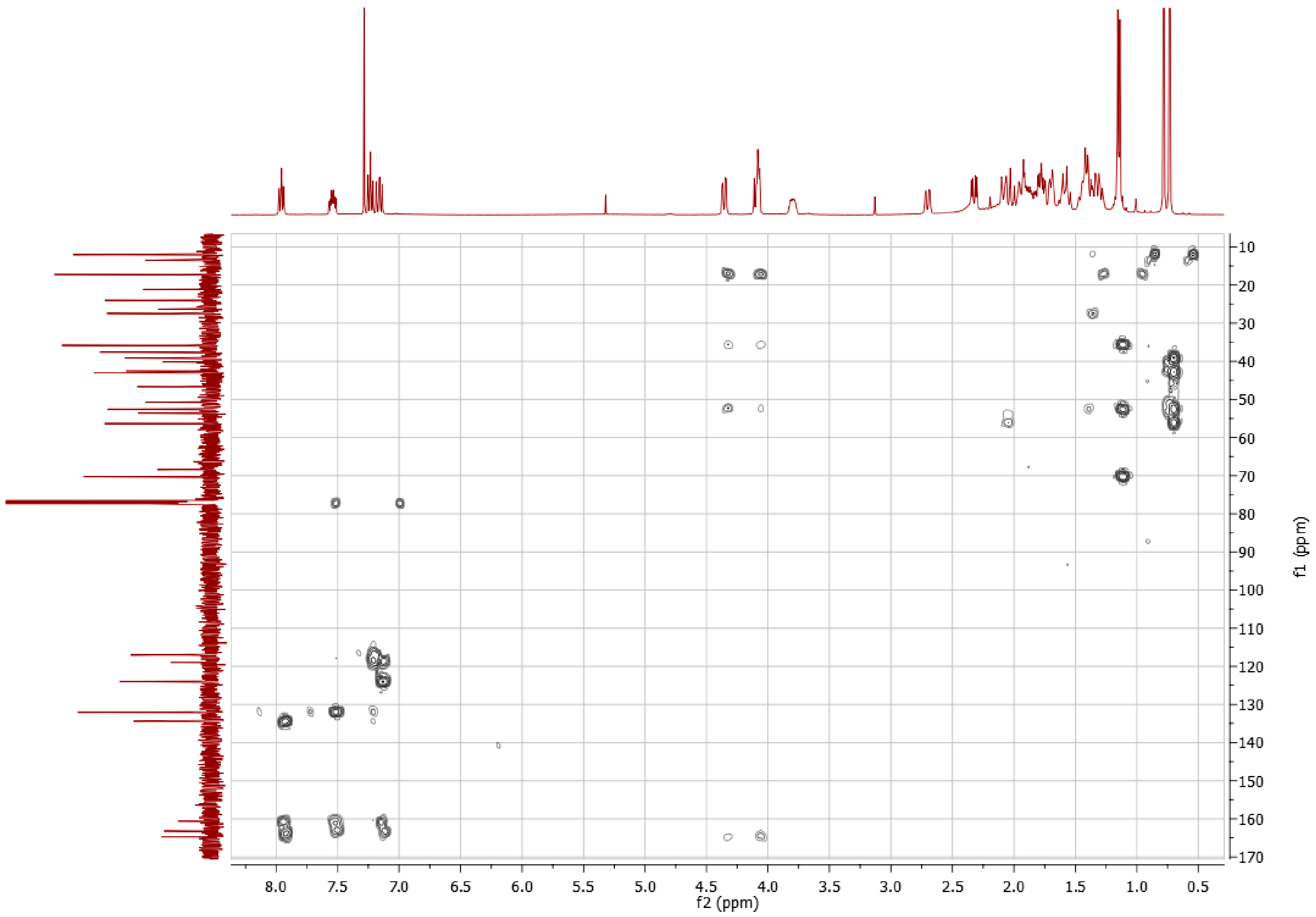

Figure S65. 2D HMBC NMR spectrum of 2α,3α-dihydroxy-5α-cholan-6-oxo-23,24-dinor-22-(2-Fluoro)-benzoate-22-yl (**10**)

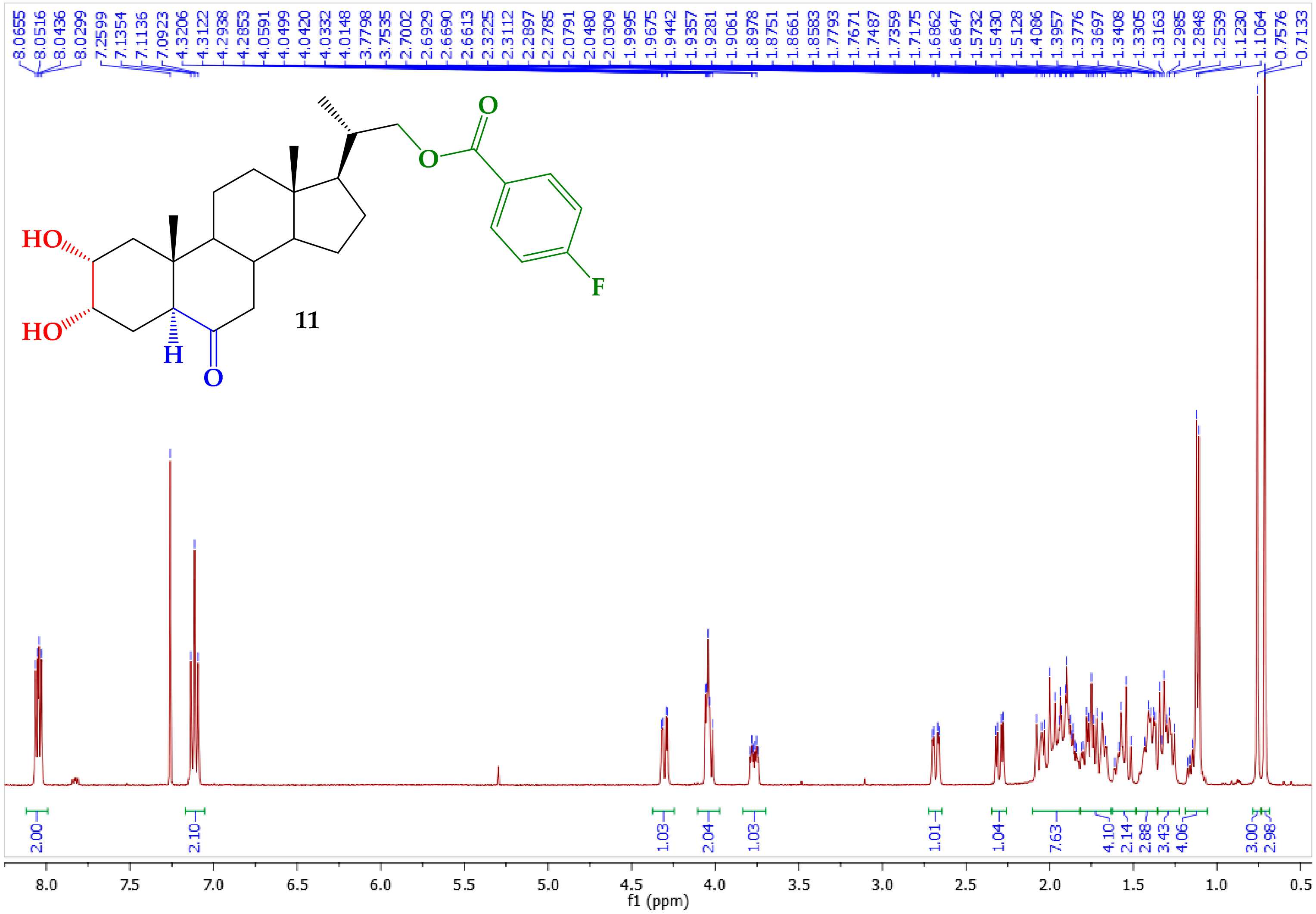

Figure S66. ^{1}H NMR spectrum of 2α,3α-dihydroxy-5α-cholan-6-oxo-23,24-dinor-22-(4-Fluoro)-benzoate-22-yl (**11**)

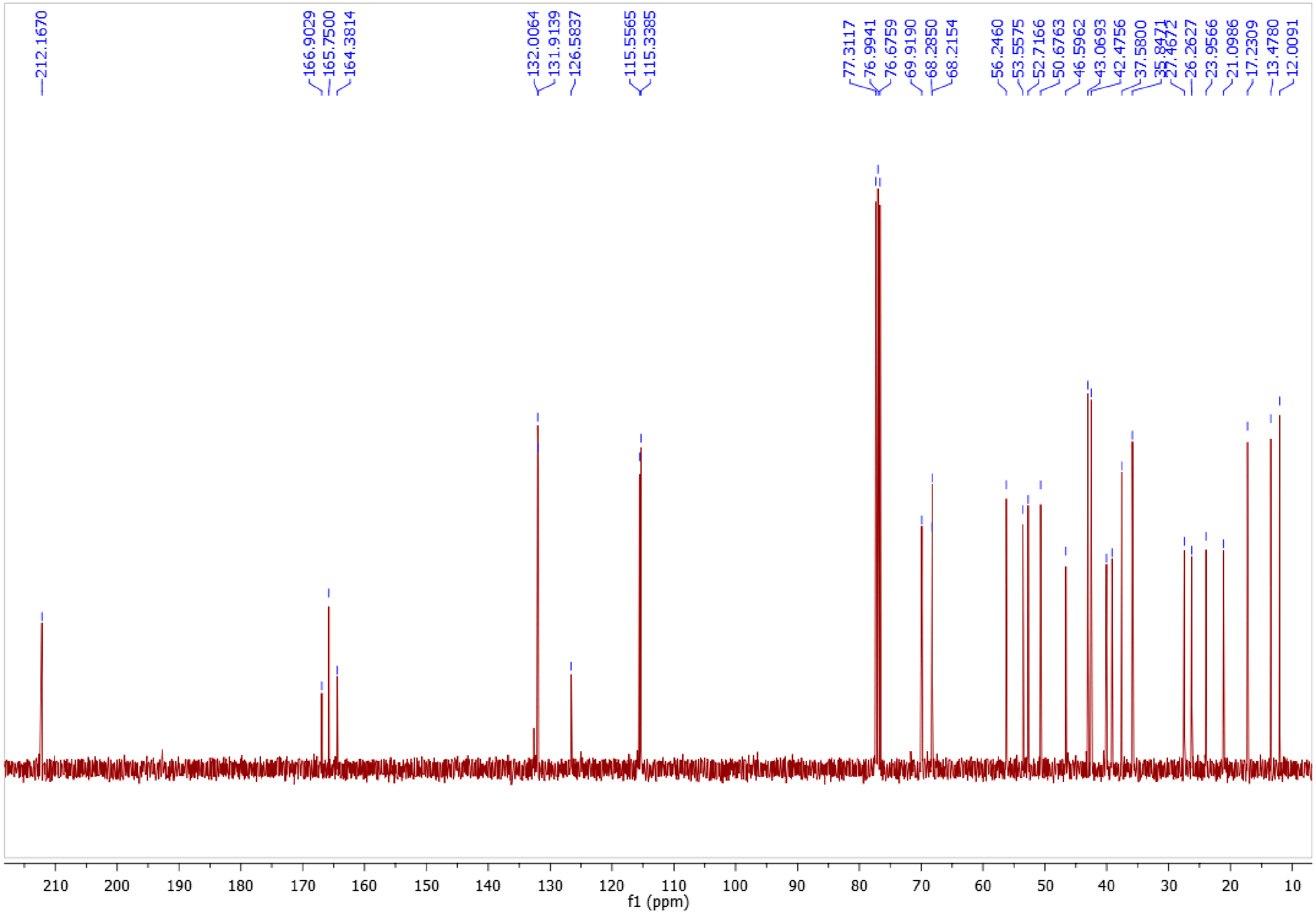

Figure S67. ^{13}C NMR spectrum of 2*α*,3*α*-dihydroxy-5*α*-cholan-6-oxo-23,24-dinor-22-(4-Fluoro)-benzoate-22-yl (**11**)

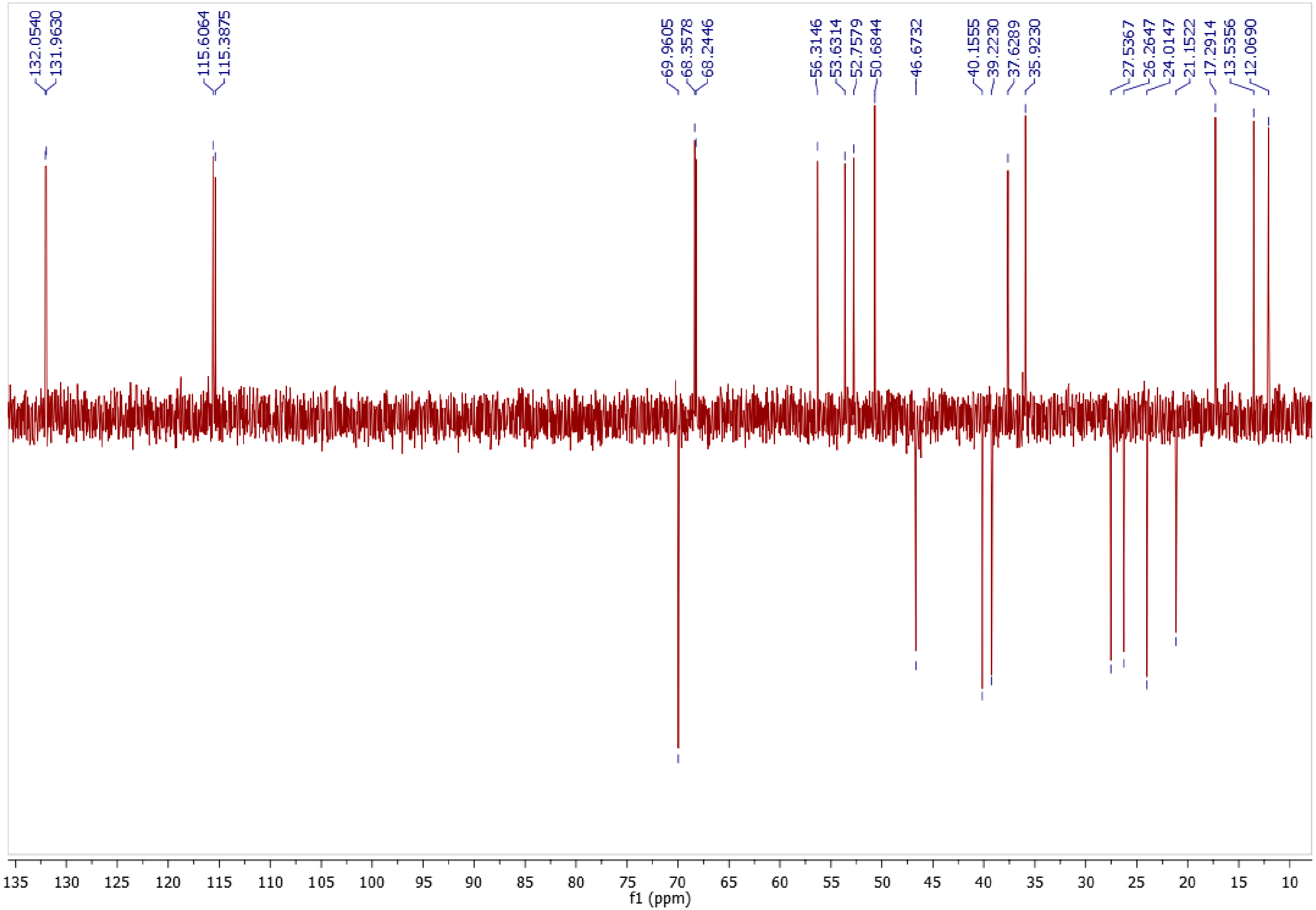

Figure S68. ^{13}C DEPT-135 NMR spectrum of 2*α*,3*α*-dihydroxy-5*α*-cholan-6-oxo-23,24-dinor-22-(4-Fluoro)-benzoate-22-yl (**11**)

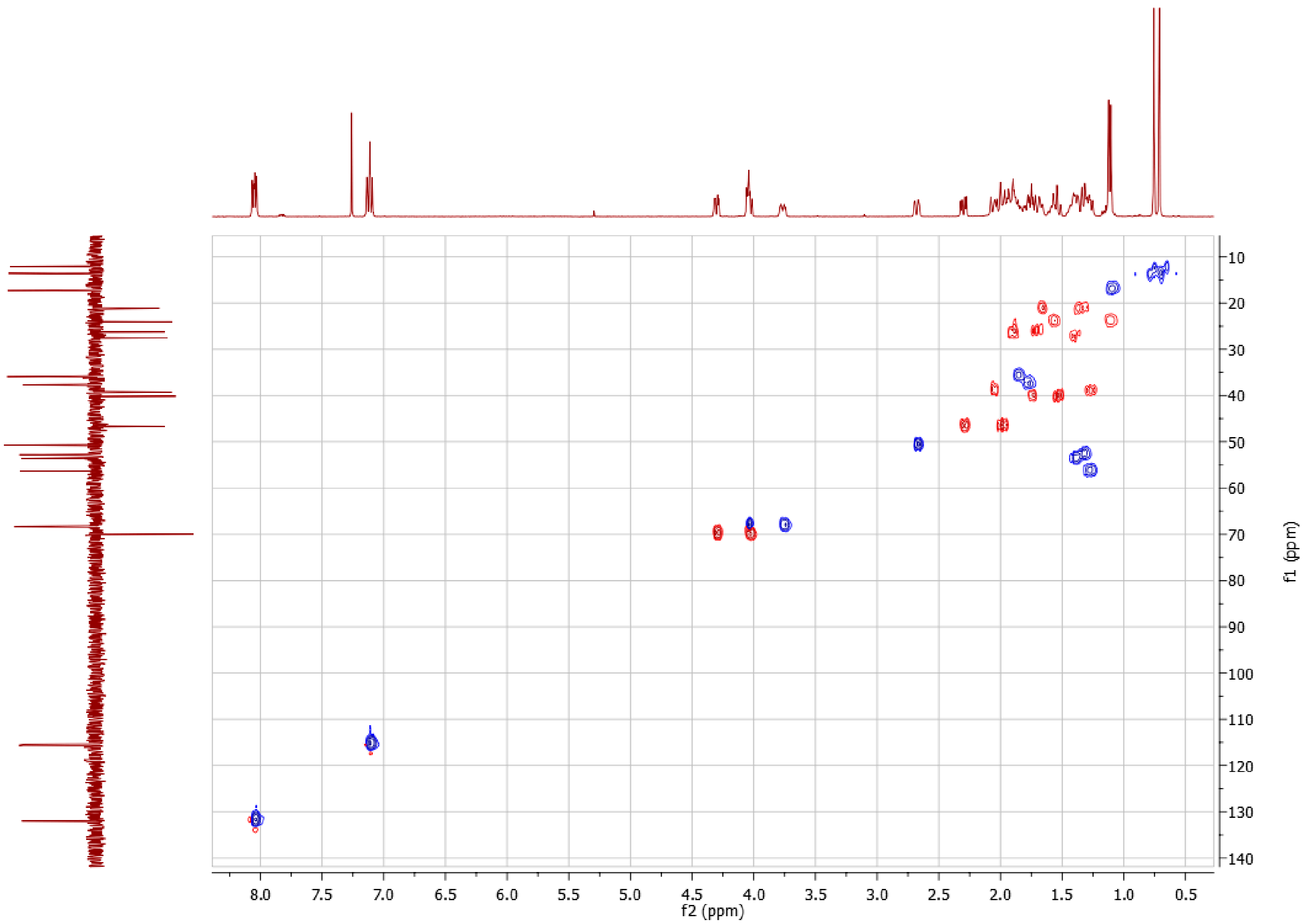

Figure S69. 2D HSQC NMR spectrum of 2α,3α-dihydroxy-5α-cholan-6-oxo-23,24-dinor-22-(4-Fluoro)-benzoate-22-yl (**11**)

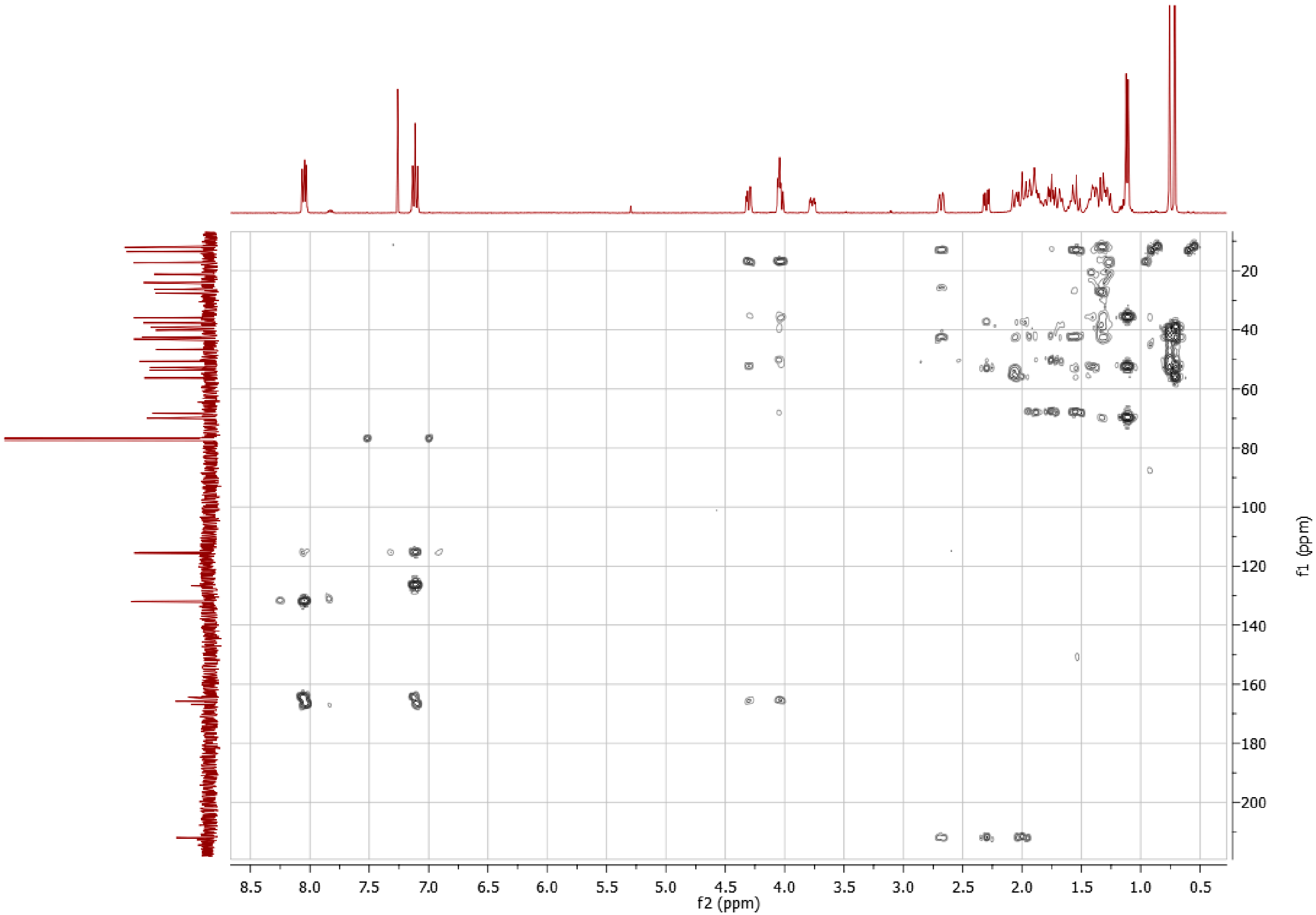

Figure S70. 2D HMBC NMR spectrum of 2α,3α-dihydroxy-5α-cholan-6-oxo-23,24-dinor-22-(4-Fluoro)-benzoate-22-yl (**11**)

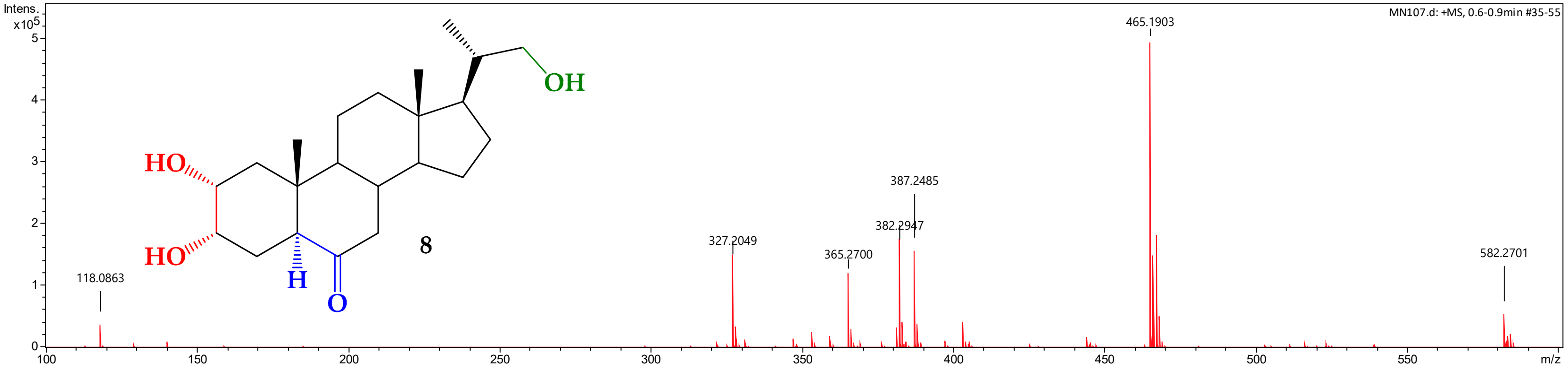

Figure S71. HRSM spectrum of 2α,3α,22-thrihydroxy-5α-cholan-23,24-dinor-6-one (**8**)

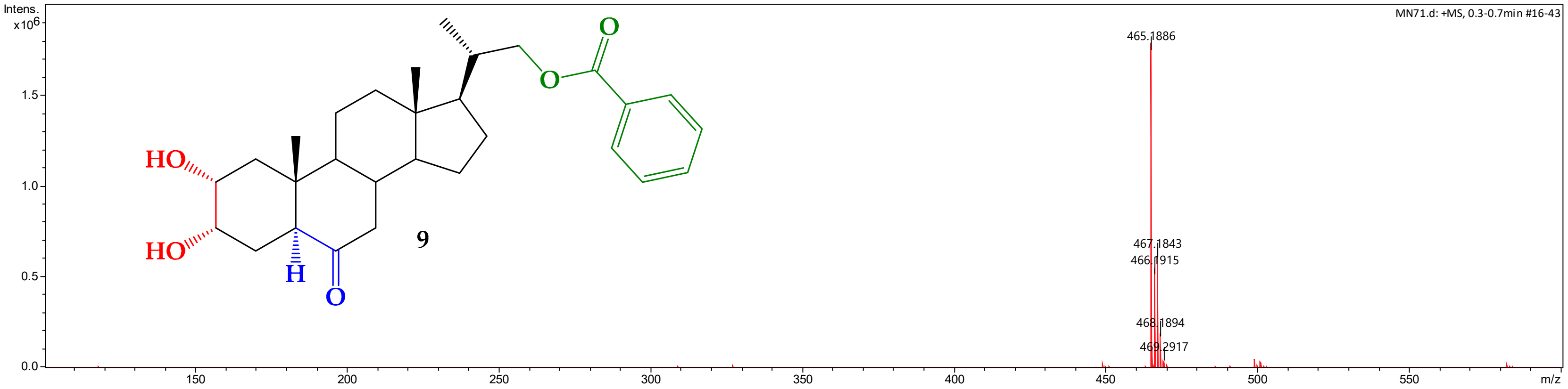

Figure S72. HRSM spectrum of 2α,3α-dihydroxy-5α-cholan-6-oxo-23,24-dinor-22-benzoate-22-yl (**9**)

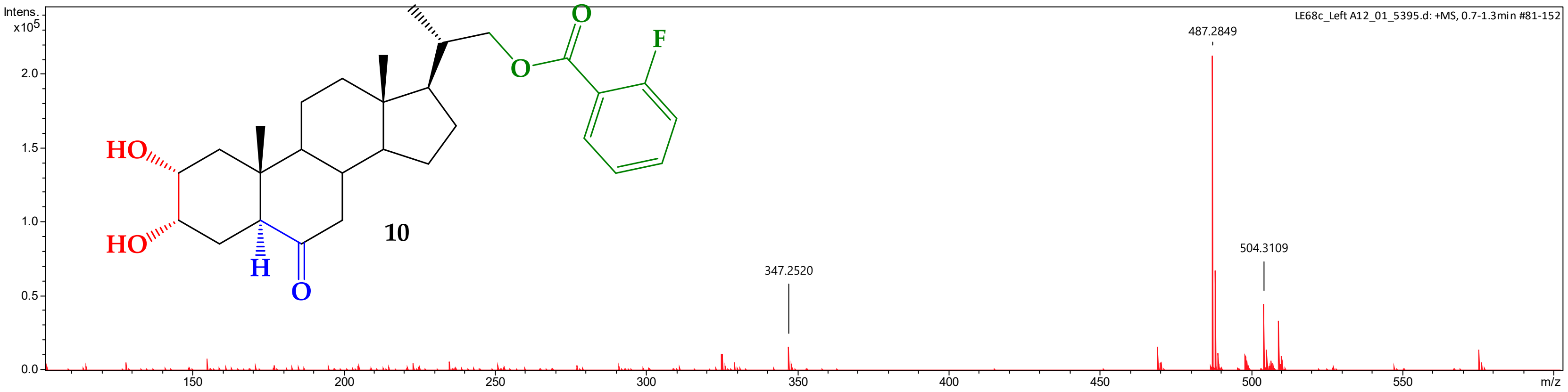

Figure S73. HRSM spectrum of 2α,3α-dihydroxy-5α-cholan-6-oxo-23,24-dinor-22-(2-Fluoro)-benzoate-22-yl (**10**)

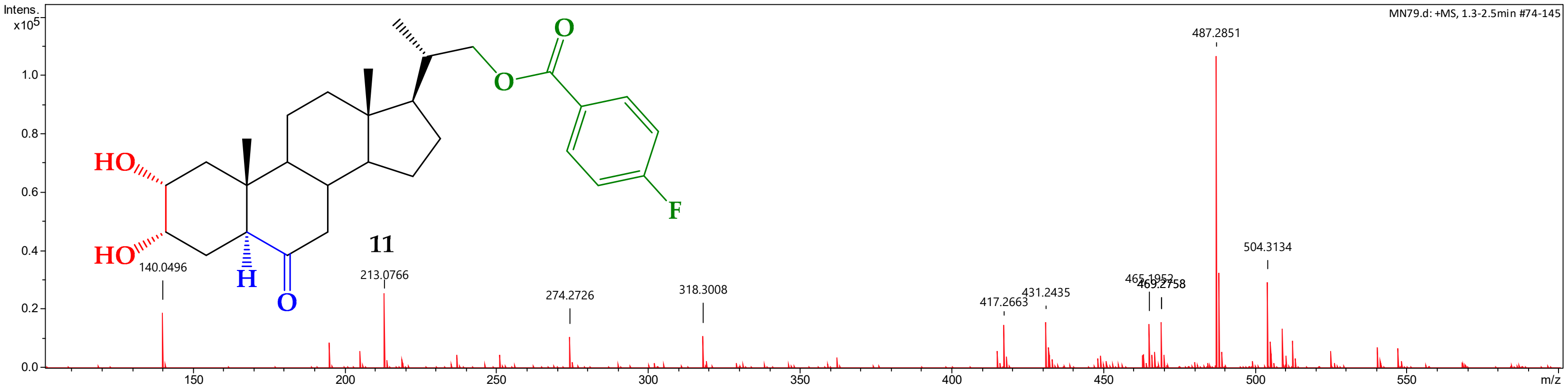

Figure S74. HRSM spectrum of 2α,3α-dihydroxy-5α-cholan-6-oxo-23,24-dinor-22-(4-Fluoro)-benzoate-22-yl (**11**)

	RLIT (Angle Opening, Degrees)	
**Compound**	**1x10^{-7} M**	**1x10^{-6} M**
	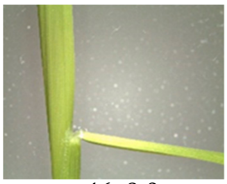 46±8.0	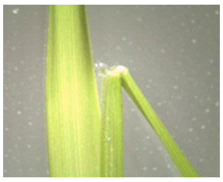 89±4.9
	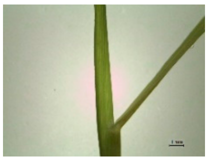 26±6.1	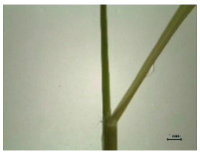 16±4.1
	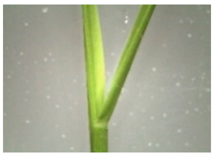 21±2.1	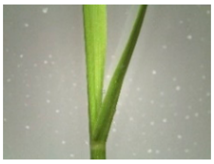 10±2.6
	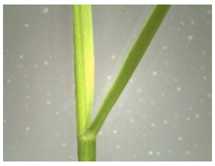 42±0.0	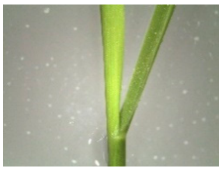 24±2.7
	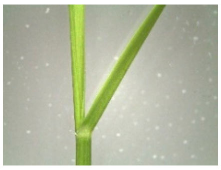 33±2.0	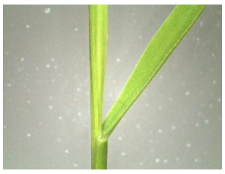 36±2.0
**Negative control (H_2O)**		3±2.6

Figure S75. Rice-lamina assays using the second leaf lamina joints (Angle Opening, Degrees) of excised leaf segments treated with BRs analogs (**8**-**10** and **19**) at different concentrations: 1 x 10^{-7}, and 1 x 10^{-6} M. Brassinolide was used as positive control at the same concentrations.

a) Brassinolide (**1**)

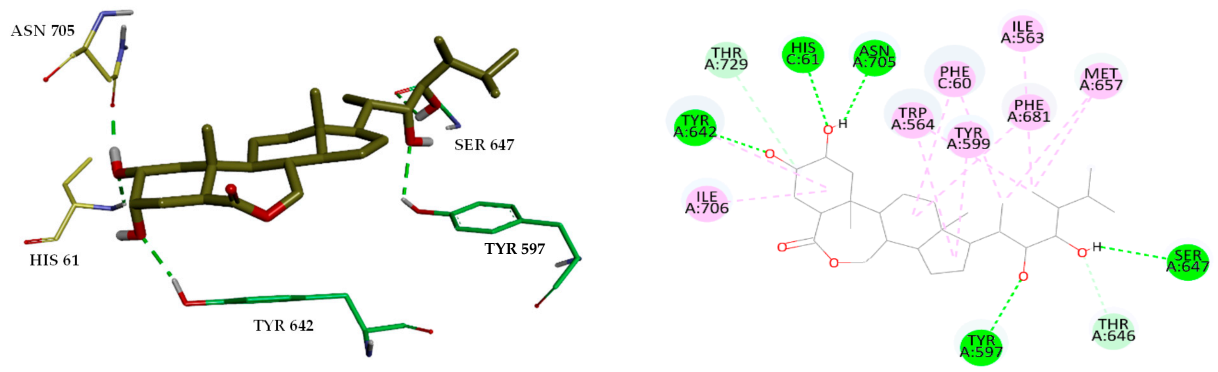

b) Compound **9** ***Rice Lamina Inclination (Angle Opening, Degrees)***

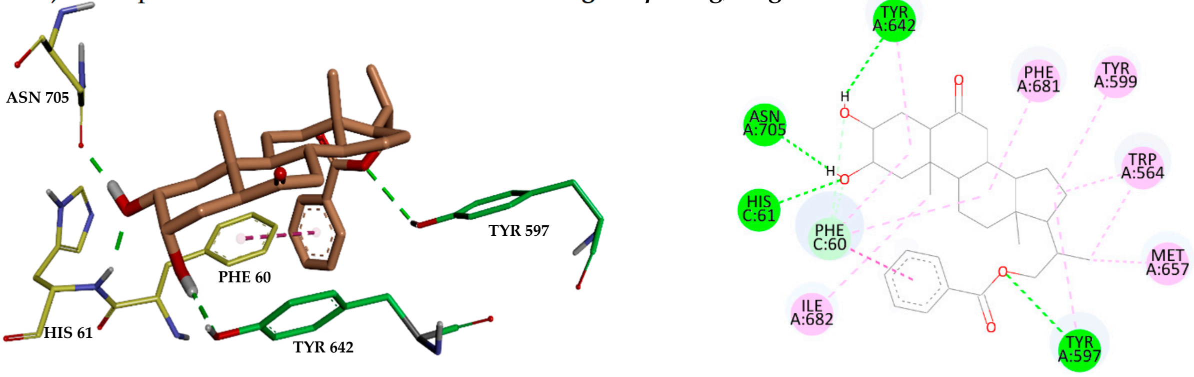

c) Compound **10**

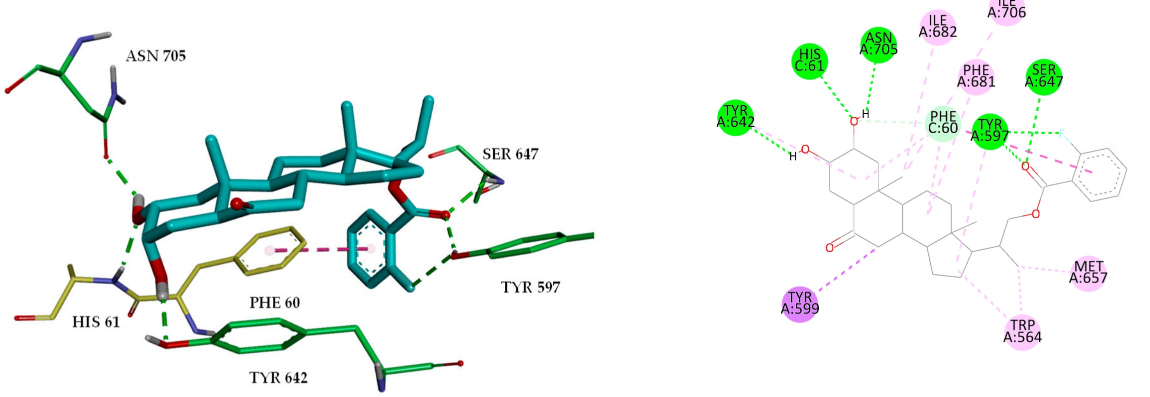

d) Compound **11**

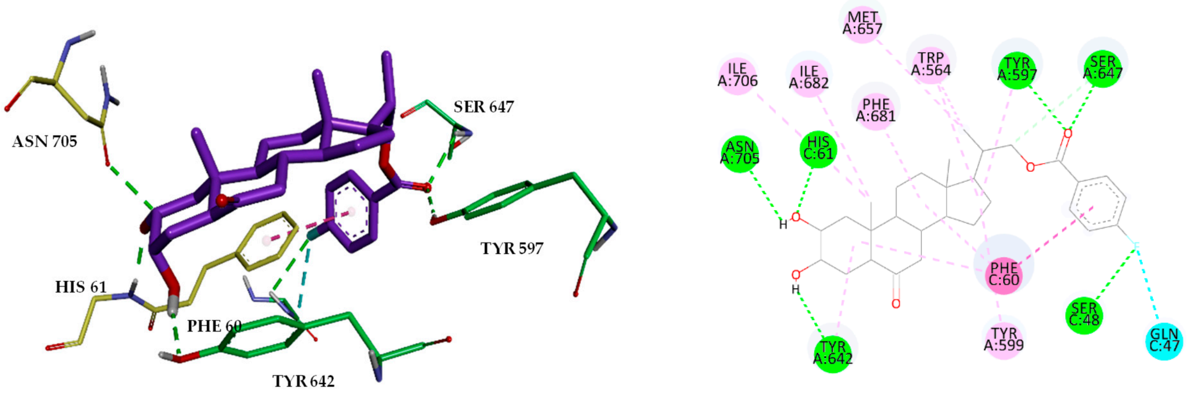

e) Compound **8**

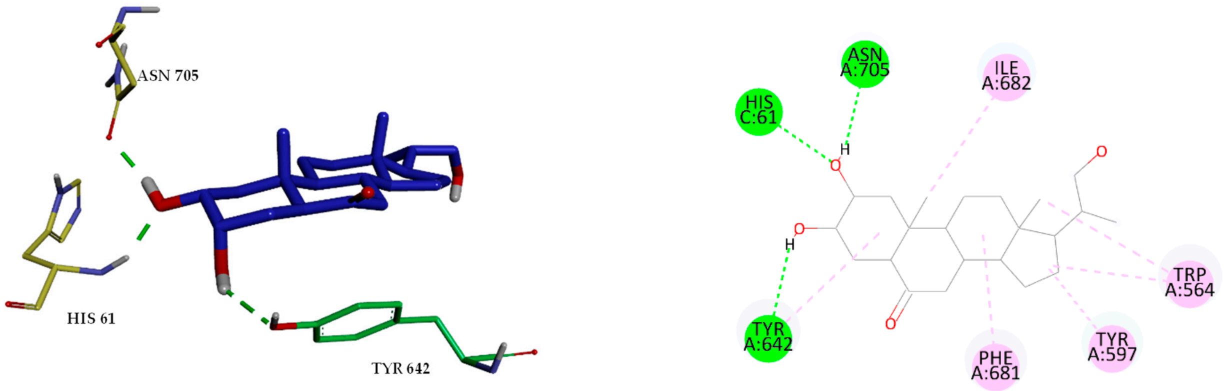

Figure S76. Protein-ligand interactions with a) brassinolide (**1**); b) Compound **8**; c) Compound **9**; d) Compound **10**, and e) Compound **19**. Hydrogen bonds are represented in green segmented lines. π-π stacking are represented in dark pink segmented lines. Hydrophobic interactions are represented in pink segmented lines. Visualization of the docked poses was performed using Discovery Studio Visualizer (BIOVIA, San Diego, CA, USA).

Table S1. Pose analysis of docked brassinolide (**1**) and synthetic analogs (**8-10** and **19**). ΔE_b: Binding Energy in kcal/mol.

Code	ΔE_b	Structure
**1**	-12.6	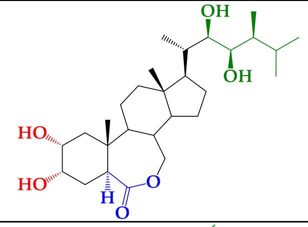
8	-11.9	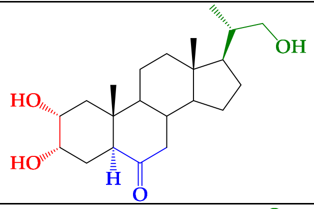
9	-12.9	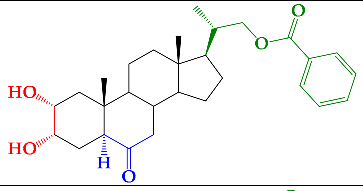
10	-13.2	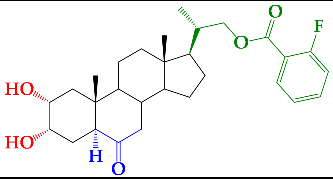
11	-13.1	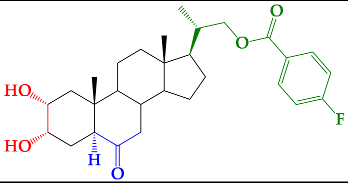

Table S2. Docked compounds-heterodimer protein contacts of brassinolide (**1**) and synthetic analogs (**8**-**10** and **19**).

Compound	**Protein Contacts**	
	Hydrogen bonds	Non-polar interactions
**1**	Tyr642<br>Asn705<br>His61<br>Tyr597<br>Ser647	Ile706, Tyr599, Phe681, Met657, Thr646, Trp564, Ile563, Phe60, Tyr642, Thr729
8	Tyr642<br>Asn705<br>His61	Tyr642, Trp564, Tyr597, Phe681, Ile682
9	Tyr642<br>Asn705<br>His61<br>Tyr597	Tyr642, Tyr599, Trp564, Tyr597, Met657, Phe681, Ile682, Phe60
10	Tyr642<br>Asn705<br>His61<br>Tyr597<br>Ser647	Tyr642, Tyr599, Trp564, Tyr597, Met657, Phe681, Ile682, Phe60, Ile706
11	Tyr642<br>Asn705<br>His61<br>Tyr597<br>Ser647<br>Ser48	Tyr642, Tyr599, Trp564, Tyr597, Met657, Phe681, Ile682, Phe60, Ile706, Ser48, Gln47
